# CRISPR-Cas3 induces broad and unidirectional genome editing in human cells

**DOI:** 10.1038/s41467-019-13226-x

**Published:** 2019-12-06

**Authors:** Hiroyuki Morisaka, Kazuto Yoshimi, Yuya Okuzaki, Peter Gee, Yayoi Kunihiro, Ekasit Sonpho, Huaigeng Xu, Noriko Sasakawa, Yuki Naito, Shinichiro Nakada, Takashi Yamamoto, Shigetoshi Sano, Akitsu Hotta, Junji Takeda, Tomoji Mashimo

**Affiliations:** 1 Department of Genome Biology, Graduate School of Medicine, Osaka University, Osaka, 565-0871 Japan; 2 Department of Dermatology, Kochi Medical School, Kochi University, Kochi, 783-8505 Japan; 3 Genome Editing Research and Development Center, Graduate School of Medicine, Osaka University, Osaka, 565-0871 Japan; 4 Institute of Experimental Animal Sciences, Graduate School of Medicine, Osaka University, Osaka, 565-0871 Japan; 5 Center for iPS Cell Research and Application (CiRA), Department of Clinical Application, Kyoto University, Kyoto, 606-8507 Japan; 6 Department of Biology, Faculty of Science, Mahidol University, Bangkok, 10400 Thailand; 7 Database Center for Life Science, Mishima, 411-8540 Japan; 8 National Institute of Genetics, Mishima, 411-8540 Japan; 9 Institute for Advanced Co-Creation Studies, Osaka University, Osaka, 565-0871 Japan; 10 Department of Mathematical and Life Sciences, Graduate School of Science, Hiroshima University, Higashi-Hiroshima, 739-8526 Japan; 11 Research Institute for Microbial Diseases, Osaka University, Osaka, 565-0871 Japan; 12 Present Address: Division of Animal Genetics, Laboratory Animal Research Center, Institute of Medical Science, The University of Tokyo, Tokyo, 108-8639 Japan

**Keywords:** Biologics, CRISPR-Cas systems

## Abstract

Although single-component Class 2 CRISPR systems, such as type II Cas9 or type V Cas12a (Cpf1), are widely used for genome editing in eukaryotic cells, the application of multi-component Class 1 CRISPR has been less developed. Here we demonstrate that type I-E CRISPR mediates distinct DNA cleavage activity in human cells. Notably, Cas3, which possesses helicase and nuclease activity, predominantly triggered several thousand base pair deletions upstream of the 5′-ARG protospacer adjacent motif (PAM), without prominent off-target activity. This Cas3-mediated directional and broad DNA degradation can be used to introduce functional gene knockouts and knock-ins. As an example of potential therapeutic applications, we show Cas3-mediated exon-skipping of the Duchenne muscular dystrophy (*DMD*) gene in patient-induced pluripotent stem cells (iPSCs). These findings broaden our understanding of the Class 1 CRISPR system, which may serve as a unique genome editing tool in eukaryotic cells distinct from the Class 2 CRISPR system.

## Abstract (web summary)

Class 1 CRISPR systems are not as developed for genome editing as Class 2 systems are. Here the authors show that Cas3 can be used to generate functional knockouts and knock-ins, as well as Cas3-mediated exon-skipping in DMD cells.

## Introduction

The clustered regularly interspaced short palindromic repeats (CRISPR)-associated (Cas) system performs adaptive immunity in prokaryotes. It is taxonomically grouped into Class 1 and Class 2, each subdivided into three types (type I, III, IV and type II, V, VI, respectively). After the first publication of sequence-specific DNA cleavage in vitro by a Class 2 type II Cas9 endonuclease^1^, several studies demonstrated that Cas9 and type V Cas12a (formerly named Cpf1) are highly effective tools for genetic engineering in various organisms, including human cells^2–9^. Recently, type VI Cas13a (formerly termed C2c2), an RNA-guided RNA ribonuclease, was used for targeted RNA degradation^10^ and RNA-based manipulations^11^. More recently, type V Cas14 was used for single-stranded DNA (ssDNA) detection and cleavage^12^. However, these CRISPR editing systems are based on a single-subunit effector grouped in the Class 2 system. On the other hand, the application of the multiple-subunit effector complex of the Class 1 system for genome engineering has not been demonstrated. In the revision stage of this manuscript, it was reported that *Thermobifida fusca* type I CRISPR-Cas generated long-range genome deletions in human embryonic stem cells^13^.

**Fig. 1:**
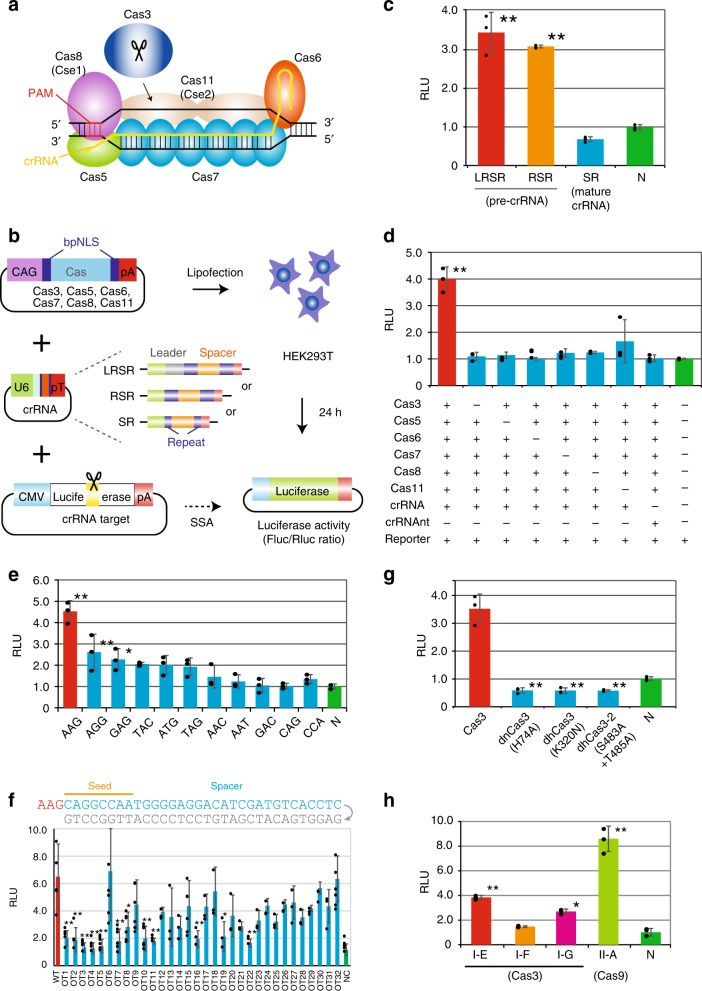
CRISPR-Cas3 system mediates DNA cleavage in human cells. **a** Type I-E CRISPR effector is composed of crRNA, Cas3, and a large Cascade complex, which contains Cas5, Cas6, multiple Cas7, Cas8 (Cse1) recognizing the PAM, and two Cas11 (Cse2). **b** Schematic of the single strand annealing (SSA) assay used to evaluate DNA cleavage and annealing activity. After the transfection of 293T cells with individual Cas, crRNA, and reporter plasmids, dual luciferase activities (Firefly (Fluc) as a reporter and *Renilla* (Rluc) as the internal control) were sequentially measured (see Supplementary Fig. 2a). **c** Efficiencies of two plasmid sequences of pre-crRNA, pLRSR, which includes a leader, repeats and a single spacer, and pRSR, which includes repeats and a spacer, both transcribe pre-crRNA, and plasmids of mat-crRNA, pSR (see Supplementary Fig. 3b). Data are presented as mean ± SD. RLU relative light units. *P < 0.05, **P < 0.01, ANOVA with post-hoc Tukey test. **d** A series of SSA assays lacking specified components of the Cascade-Cas3 effector complex. Lipofection of the pre-crRNA and six Cas (3, 5–8, and 11)-coding plasmids was performed in 293T cells. crRNAnt nontarget crRNA. **e** Effect of PAM sequences on Cas3-mediated DNA cleavage activity in 293T cells (see Supplementary Table 1). **f** Effect of a single mismatch (gray) for 32-nt spacer sequences on Cas3-mediated DNA cleavage activity (see Supplementary Table 1). **g** Effect of Cas3 mutants in the HD nuclease domain (H74A) or in SF2-helicase domain motif 1 (K320N) or motif III (S483/T485A)^31^. **h** Comparison of DNA cleavage activity between Class 1 *E. coli* type I-E*, S. putrefaciens* type I-F*, P. furiosus* type I-G (Cas3), and Class 2 *S.pyogenes* type II-A (Cas9) (see Supplementary Table 1 and Supplementary Fig. 4). Source data are in the Source Data file.

The Class 1 system represents about 90% of CRISPR-Cas loci and is more widely present than Class II in both bacteria and archaea^14,15^. Within the Class I system, type I is most widespread and functions as a CRISPR RNA (crRNA)-bound multiprotein complex, termed Cas complex for antiviral defense (Cascade), and as a Cas3 endonuclease, which is recruited upon target binding by Cascade to cleave foreign DNA^16–21^. Among the seven subtypes identified to date (I-A to G), type I-E of *Escherichia coli* is the most biochemically characterized subtype. Type I-E Cascade is composed of five proteins with different stoichiometry (Fig. 1a). Cas6 processes mature crRNA (mat-crRNA) from precursor RNA (pre-crRNA) and holds the 3′ hairpin of crRNA. Cas5 binds the 5′ handle, and Cas7 forms the backbone along the crRNA. Cas11 (formerly named Cse2) forms the belly of Cascade and stabilizes the crRNA and target strand DNA loop (R-loop) structure. Cas8 (Cse1) recognizes protospacer-adjacent motif (PAM) sequences and recruits Cas3 to the authenticated target^22^ (Supplementary Fig. 1). Finally, once activated, Cas3 processively degrades the target DNA. Although the type I-E CRISPR system was reported to induce the degradation of plasmid DNA in vitro^23,24^ as well as transcriptional silencing in *E. coli*^25–27^, it is unclear whether it mediates DNA cleavage for genome editing in eukaryotic cells, such as human cells. In this study, we demonstrate that the type I-E CRISPR, *E. coli* Cascade, Cas3, and pre-crRNA, but not mature crRNA, possesses robust and efficient cleavage activity against plasmid DNA and endogenous genomic DNA in human cells. The CRISPR-Cas3 system introduces a long range and unidirectional genomic DNA deletion upstream of the PAM without prominent off-target activity. In contrast to the CRISPR-Cas9 system, this distinctive feature of CRISPR-Cas3-mediated genome editing might broaden the application of genome editing by facilitating efficient gene knockouts and/or knock-ins, as well as future therapeutic applications.

## Results

### Type I-E CRISPR exhibits endonuclease activity in human cells

To assess the DNA cleavage activity of the type I CRISPR-Cas system in human cells, we used a luciferase-based single-strand annealing (SSA) recombination assay^28^, in which a split luciferase sequence recombines into a translationally active form after the CRISPR-Cas system causes a double-strand break and SSA (Fig. 1b). Either a short 91-bp or a long 3.8-kbp sequence including a 32-nt spacer was integrated between the split luciferase sequence (pGL4-SSA:Addgene #42962), and the 5′-AAG-PAM was used as previously reported in *E. coli*^16–21^ (Supplementary Fig. 2a). Human codon-optimized Cas3, Cas5, Cas6, Cas7, Cas8, and Cas11 from *E. coli* with bipartite SV40 nuclear localization signals (bpNLS) at the N- and C-termini^29,30^ were individually cloned downstream of the CAG promoter (Fig. 1b). The luciferase activity of Firefly (Fluc) reporter and *Renilla* (Rluc) internal control were measured 24 h after the lipofection of Cascade, Cas3, crRNA, and reporter plasmids into 293T cells.

First, we tested type I CRISPR with pre-crRNA, which includes a 32-nt spacer sequence and two 29-nt repeats with or without an AT-rich leader (LRSR or RSR, respectively), or mat-crRNA (SR), which includes 8 nt of the 5′ handle and 21 nt of the 3′ hairpin with the spacer sequences (Supplementary Fig. 3). Surprisingly, Cas genes with the pre-crRNA (LRSR and RSR) exhibited significant DNA cleavage activity, while the mat-crRNA (SR) showed no activity (Fig. 1c). The CRISPR-Cas3 system showed comparable SSA activity with both short and long pGL4-SSA plasmids, but CRISPR-Cas9 showed higher activity with the short plasmid (Supplementary Fig. 2b). To confirm whether all six Cas effectors with the pre-crRNA were needed for DNA cleavage, we removed each factor individually in the SSA assay (Fig. 1d). The absence of any Cas effector resulted in a complete loss of activity. Multiple Cas effectors without pre-crRNA or with pre-crRNA targeting different spacer sequences (crRNAnt) also showed no activity. These observations indicated that CRISPR-Cas3 effectors and the crRNA complex specifically bound to targeted sequences and cleaved DNA in a coordinated manner.

To assess the targeting specificity of the CRISPR-Cas3 system, we investigated the effect of various PAM sequences on cleavage activity (Fig. 1e and Supplementary Table 1). The SSA assay demonstrated a range of DNA cleavage activities for different PAM sequences, among which the 5′-AAG PAM had the highest activity in human cells, and AGG, GAG, TAC, ATG, and TAG had lower but detectable activity, consistent with previously reported biochemical results^31–33^. Next, we evaluated the mismatch effect of each 32-nt spacer sequence on DNA cleavage activity (Fig. 1f and Supplementary Table 1). As expected, any single mismatch within the seed region (positions 1–8)^32,34,35^ markedly reduced SSA activity, whereas a mismatch after position 24 had little effect on activity. Furthermore, a mismatch at position 6 had no effect on SSA activity because of base-pairing disruption between the crRNA and spacer DNA strand by a thumb element of Cas7 (Fig. 1a), which was previously demonstrated in vitro^36,37^ and by crystal structure characterization^32,35,38^.

In vitro characterization of the catalytic features of Cas3 protein^31,32,39^ indicated that the N-terminal HD-nuclease domain cleaved a single-stranded region of the DNA substrate, followed by progressive ATP-dependent unwinding of the target DNA in the 3′ to 5′ direction by a C-terminal SF2-helicase domain. To test whether these Cas3 domains were required for DNA cleavage, we assessed three Cas3 protein mutants^31^, HD domain H74A mutant (dnCas3), SF2 domain motif I K320N mutant (dhCas3), and SF2 motif III S483A/T485A double mutant (dh2Cas3) (Fig. 1g). All three mutations of Cas3 protein abolished the SSA recombination activity, indicating Cas3 catalyzed the degradation of target DNA through its HD-nuclease and SF2-helicase domains.

Seven type I subtypes (A–G) use highly diverse, multi-subunit surveillance Cascade complexes composed of three Cas effectors for the type I-B, -C, -D, -F systems, four effectors for the type I-A and I-G systems, and five effectors for the type I-E system. Of these, type I-F^40,41^ and type I-G^42^ degrade targeted plasmid DNA in bacteria; therefore, we cloned all components of the Cas effectors, Cas3 and Cas5-7 of *Shewanella putrefaciens* (type I-F) and Cas5-8 of *Methanosarcina barkeri* (type I-G) with human-codon optimization (Supplementary Fig. 4 and Supplementary Table 1). The SSA assay using these type I CRISPR-Cas systems and type II-A *Streptococcus pyogenes* Cas9 indicated that the type I-G CRISPR system possessed DNA cleavage activity, but that the type I-E and type II-A systems demonstrated higher activity in 293T cells (Fig. 1h).

### CRISPR-Cas3 mediates a broad pattern of DNA degradation

**Fig. 2:**
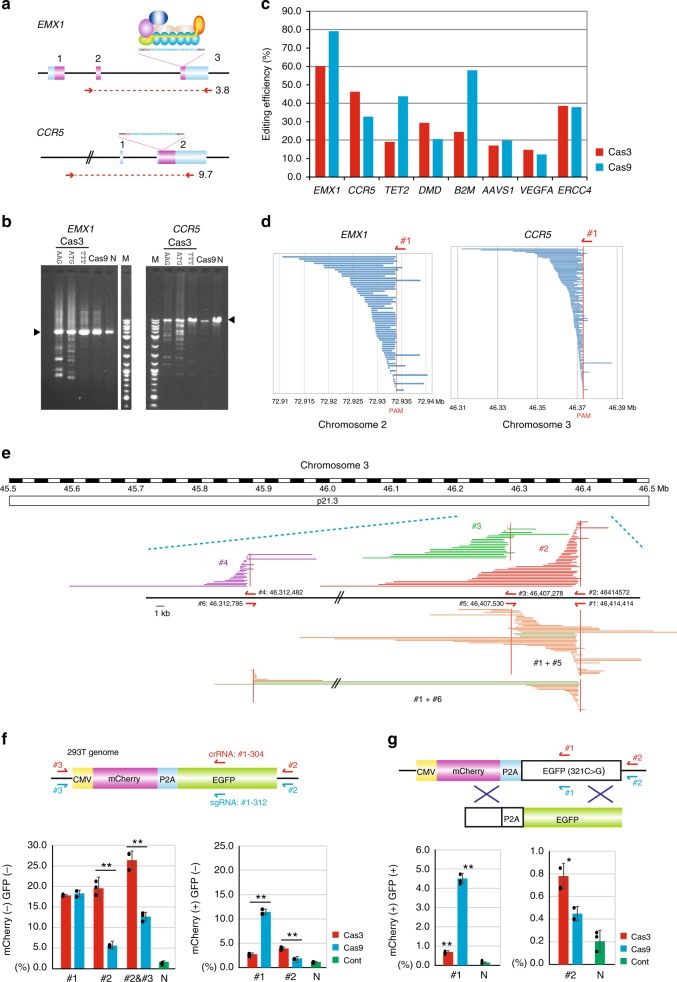
CRISPR-Cas3 facilitates a large deletion at endogenous targeted loci in 293T cells. **a** Schematic of the CRISPR-Cas3 system targeting human *EMX1* and *CCR5* loci. Primer sets (red arrows) used for a 3.7 kb PCR product of *EMX1* and a 9.7 kb PCR product of *CCR5* are shown. **b** Electrophoresis of the PCR products. CRISPR-Cas3 targeting AAG PAM or ATG PAM, but not TTT PAM, mediates deletions (see Supplementary Fig. 5a). **c** Comparison of the editing efficiency between Cas3 (red) and Cas9 (blue) via NGS of the PCR amplicons at various target sites (see Supplementary Table 3). **d** Cas3-mediated DNA deletion patterns via microarray-based capture sequencing at *EMX1* and *CCR5* loci in 293T cells. The deletions (blue bars) are aligned at the starting point of the distal end. **e** Microarray-based capture sequencing with Cas3/crRNAs (#1–6) targeting a 1-Mb region at the *CCR5* loci (see Supplementary Fig. 11 for *EMX1* loci). Cas3-mediated DNA deletion patterns are aligned with human genome assembly hg38. Dual crRNA (#1 and #5 or #6) are used for sticking in the interval region. **f** A surrogate reporter assay using mCherry-2A-EGFP plasmids to characterize Cas3-mediated knockouts in **f** or knock-ins in **g**. crRNA #1 (red arrows)/sgRNA (blue arrows) were designed at EGFP-coding sequences, and crRNAs/sgRNAs #2 and #3 were outside the coding sequences (see Supplementary Table 9). Data are presented as mean ± SD. *P < 0.05, **P < 0.01, *t* tests with Bonferroni corrections. Percentage of CRISPR-mediated knockout cells for long-range deletions (mCherry(−)GFP(−)) and for small indel mutations (mCherry(+)GFP(−)). **g** CRISPR-mediated gene knock-in assay by flow cytometry. Cas3- or Cas9-mediated nucleotide substitution by dsDNA-dependent HDR restores GFP expression (see Supplementary Table 11) in 293T cells. Percentage of CRISPR**-**mediated knock-in cells (mCherry(+)GFP(+)). Source data are in the Source Data file.

Results from the SSA assays using the type I-E CRISPR system enabled us to assess targeting for endogenous genes in human cells. We selected crRNA target sites for the *EMX1* and *CCR5* genes (Fig. 2a and Supplementary Fig. 5a). Lipofection of pre-crRNA and six Cas (3, 5–8, and 11)-coding plasmids into 293T cells resulted in a broad pattern of Cas3-mediated DNA degradation of two PCR products: 3.7 kb for *EMX1* and 9.7 kb for *CCR5* (Fig. 2b). In contrast to targeting with 5′ AAG or 5′ ATG, 5′ TTT-PAM did not induce DNA degradation. Degradation was also not observed at the targeted *EMX1* and *CCR5* regions when using the mat-crRNA plasmid (Supplementary Fig. 3b). Despite the limited number of dominant deletion products observed in Fig. 2b, independent repetitive PCR with distinct Taq polymerases indicated different banding patterns or smeared PCR products (Supplementary Fig. 6a), suggesting biased PCR amplification of a small number of initial products. Next-generation sequencing (NGS) of the PCR amplicons revealed a bulk deletion of several kb upstream of AAG-PAM or ATG-PAM (55.1–88.9% editing efficiency), but not of TTT-PAM. (0.0%–3.8%) (Supplementary Fig. 5b and Supplementary Table 2). Small insertion/deletion (indel) mutations were induced by Cas9 with 43.2–50.0% editing efficiency at the targeted site (Supplementary Table 2), but we could not observed any mutation less than 100 bp mediated by Cas3 (Supplementary Fig. 7). Although large deletions may induce genome instability or unspecific toxicity in human cells, we observed normal cell proliferation and viability, and no obvious cell apoptosis after the transfection of CRISPR-Cas3 or -Cas9 (Supplementary Fig. 6b).

To accurately evaluate the general efficiency of the CRISPR-Cas3 system, we targeted eight endogenous genes and compared the efficiency with the Cas9 system at the same loci (Fig. 2c). NGS of the PCR amplicons for each gene revealed Cas3-induced large deletions with 7.3–60.3% editing efficiency (mean 26.6%), whereas Cas9 induced small indels with 15.3–78.2% editing efficiency (mean 47.0%) (Supplementary Table 3). Because Illumina short-read sequencing is often problematic for complex and repeat-rich genomic regions, as observed in Supplementary Fig. 5b, we sequenced PCR amplicons with a MinION Nanopore long-read sequencer for Cas3-mediated *EMX1* and *DMD* targets (Supplementary Fig. 8). We reconfirmed that Cas3-mediated broad DNA degradation was induced upstream of the crRNA target site, in contrast to the Cas9-mediated large deletion induced around the sgRNA target site as recently reported^43^.

### Characterization of Cas3-mediated unidirectional deletions

Considering the limitations of PCR analysis, including limited amplicon size and a strong bias in favor of smaller product sizes, which could increase the estimated editing efficiency, we sought comprehensive and unbiased sequencing methods to detect long-range Cas3-mediated mutations. Because whole genome sequencing (WGS) with 90× coverage depth has a limited detection threshold (Supplementary Fig. 9), we conducted custom array-based capture sequencing with 2000–2300× coverage of a 1-Mb region of the *EMX1* and *CCR5* genes with Cas3-crRNA #1 and Cas9-sgRNA #1 (Fig. 2d and Supplementary Fig. 10a). We identified a wide variety of Cas3-mediated large deletions through a long stretch of the targeted region upstream of the PAM (Fig. 2d) and also identified Cas9-mediated long-range deletions of 35.8 kb for *EMX1* and 23.5 kb for *CCR5*^43^ (Supplementary Fig. 10a). The editing efficiency of the Cas3-mediated large deletions was 16.1% for *EMX1* and 4.6% for *CCR5*, whereas the editing efficiency of the Cas9 small indels was 23.4% for *EMX1* and 23.7% for *CCR5* (Supplementary Table 4). The strong helicase and nuclease activities of Cas3 induced excessive DNA degradation, mostly upstream of the PAM/target site. Many Cas3-mediated deletions started within less than 500 bp from the PAM site, with efficiencies of 63.2% for *EMX1* and 60.0% for *CCR5*, and on rare occasions started downstream of the PAM sites, although no defined deletion endpoints or hotspots around the target site could be detected (Supplementary Fig. 10b). Similarly, there were no defined endpoints in the nanopore long-read sequencing analysis with a mean starting position of 217 bp and the 90% percentile of 421 bp (Supplementary Fig. 10c). Finally, a distribution map of the deletion sizes indicated that 56.5% and 49.1% of deletions were <3 kb and 86.4% and 81.6% were <10 kb for *EMX1* and for *CCR5*, respectively (Supplementary Fig. 10d).

To further characterize Cas3-mediated deletions, we designed five additional crRNAs (#2–6) at the 1-Mb region of the *EMX1* and *CCR5* loci (Fig. 2e and Supplementary Fig. 11). The editing efficiency of the CRISPR-Cas3 system ranged from 4.4–38.2% for *EMX1* and 8.0–16.6% for *CCR5* (Supplementary Table 4). The mean size of the Cas3-mediated deletions was 3.2–6.3 kb for *EMX1* and 4.0–7.6 kb for *CCR5*, and the maximum sizes were 77.7 kb and 51.3 kb, respectively (Supplementary Table 4). Length distributions of the Cas3-mediated deletions on the distal end were not defined for crRNAs #1–6 (Fig. 2e). In addition, distinct crRNAs that pointed in the same direction (#3 and #4, 10 and 100 kb from #2, respectively) indicated various distribution patterns of deletion sizes without hotspots (Fig. 2e and Supplementary Fig. 11). Interestingly, Cas3 with two crRNAs that pointed in different directions (10 kb between #1 and #5 and 100 kb between #1 and #6) induced various deletion patterns, including defined deletions between #1 and #5 or #6 (green bars in Fig. 2e). These observations indicated that the CRISPR-Cas3 system mediates a broad range of DNA degradation upstream of the authenticated target site in human cells, which is distinct from Cas9-mediated small indels. This difference was also confirmed by the SSA assay (Supplementary Fig. 2b).

### Quantification of cell surface proteins

Given the difficulty of using sequencing approaches to determine editing efficiency, we used an *mCherry-2A-EGFP* reporter system expressing mCherry and GFP proteins from a single copy integrated into the genome of 293T cells^44^ (Fig. 2f). We designed crRNA (#1-304) and sgRNA (#1-312) that target GFP-coding sequences, transfected them into 293T cells, and measured the GFP and mCherry expression by flow cytometry. The depletion of GFP and mCherry double fluorescence (GFP(−)mCherry(−)) was comparable between Cas3 (17.9%) and Cas9 (18.3%). However, Cas9 induced 11.5% small indels that were associated with GFP(−)mCherry(+), which was significantly higher than the 2.7% induced by Cas3 (Fig. 2f). On the other hand, crRNA #2, which was designed outside the GFP sequences, induced GFP(−)mCherry(−) with 19.5% efficiency, while Cas9 with sgRNA #2 mediated only 5.7% efficiency (Fig. 2f). The Cas3-mediated deletions were confirmed by TOPO cloning and Sanger sequencing in 293T cells lacking both GFP and mCherry fluorescence (Supplementary Fig. 12a).

The editing efficiency of Cas3 compared with Cas9 was further characterized using the X-linked endogenous gene, *SLC35A2*, which encodes the UDP-galactose transporter. Disruption of SLC35A2 resulted in modification of N-glycan and was detected by lectin binding with Alexa Fluor 647-conjugated *Griffonia simplicifolia* lectin II (Supplementary Fig. 12b). Six days after Cascade/Cas3 or Cas9 plasmid transfection, we detected lectin II binding on 293T cells. Cascade/Cas3 induced 4.7–7.7% lectin II binding efficiency, while Cas9 induced 26.1–46.9% efficiency. Note that Cascade/Cas3 and Cas9 target sites were outside and within exons of *SLC35A2*, respectively (Supplementary Fig. 12b). This experimental design underestimated the gene disruption by Cascade/Cas3, because some deletions outside exons by Cascade/Cas3 did not affect *SLC35A2* function.

Because long-range gene deletions can be induced by multiplexing CRISPR-Cas9 with two sgRNAs that flank a genomic region^45^, we also tested Cas9 with two sgRNAs, #2 and #3, for GFP(−)mCherry(−) in the reporter system (Fig. 2f). Notably, Cas3 with crRNA #2 and #3 resulted in a higher efficiency of GFP(−)mCherry(−) (26.4%) than did Cas9 with two sgRNAs (12.7%). In addition, Cas9, but not Cas3, induced unintended genomic inversions between the two cleavage sites, which were detected by PCR amplification using boundary primer sets (Supplementary Fig. 13a)^46,47^. Finally, using a split puromycin resistance assay, we confirmed that Cas3 induced long-range deletions with higher efficiency for an ~7 kb wider genome region than Cas9 did (Supplementary Fig. 13b).

For further comparison of the editing efficiency between the CRISPR-Cas3 and -Cas9 systems, we targeted the *beta2-microglobulin (B2M)* gene to suppress the surface expression of all class I human leukocyte antigen (HLA) molecules^48^. We designed more than ten crRNAs and ten sgRNAs along the endogenous *B2M* locus (Supplementary Fig. 14a). After transfection of the CRISPR-Cas3 plasmids into 293T cells, we measured the cell surface HLA expression by immunofluorescence staining. Knockout (KO) efficiencies of the B2M/HLA class I protein expression were 3.9–15.1% by CRISPR-Cas3 and 3.1–30.6% by CRISPR-Cas9. Interestingly, exonic indels by Cas9 with sgRNA #3, #4, or #7 (blue in Supplementary Fig. 14b) had a strong KO effect for B2M protein, but intronic sgRNAs had little effect. Moreover, single crRNA with Cas3 (red) had a higher KO efficiency than dual sgRNAs with Cas9 (yellow).

### Functional gene knock-ins via HDR

To examine whether the CRISPR-Cas3 system can be used for knock-in (KI), we used another mCherry-2A-EGFP reporter system that expressed mCherry protein, but not EGFP protein, because of a nonsense mutation (321C>G)^44^. In this system, KI was measured by the recovery of green fluorescence with a double-stranded DNA (dsDNA) donor vector including wild-type EGFP sequences (Fig. 2g). Cas9 with sgRNA #1-312 was designed to be near the 321C>G mutation site and induced 4.5% KI efficiency, while Cas3 induced less than 1% KI. In contrast, Cas3 with crRNA #2 was designed to be distal from the targeting site and showed higher KI efficiency than Cas9 (Fig. 2g). Single nucleotide substitutions with dsDNA via HDR mediated by CRISPR-Cas3 and -Cas9 were confirmed by HiDi PCR and sequencing in edited 293T cells (Supplementary Fig. 15a). In addition, a ~1 kb functional gene was successfully targeted at the *EMX1* locus by CRISPR-Cas3 with a long ssDNA (lssDNA) donor^49^, which consisted of an antibiotic selection cassette (hEF1α-Puro) and 1 kb homology arms. Cas3-mediated KI was confirmed by PCR amplification and subsequent sequencing of the edited 293T cells (Supplementary Fig. 15b).

### No prominent off-target effects were observed

**Fig. 3:**
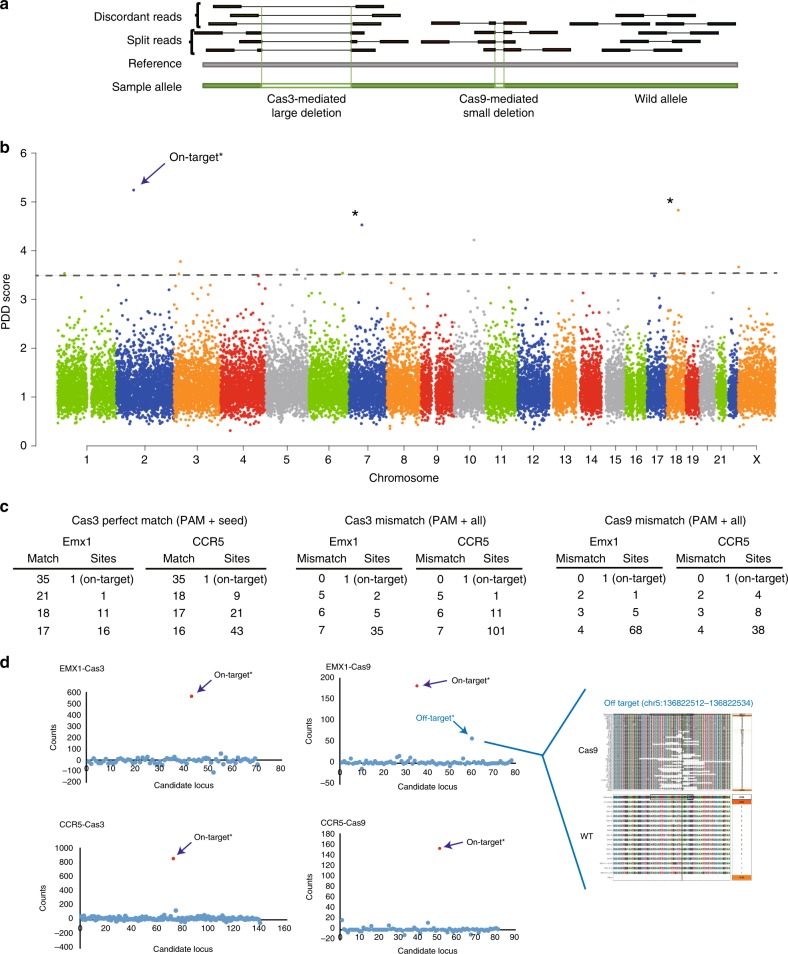
Off-target analysis of the CRISPR-Cas3 system. **a** Schematic of discordant read pairs and splitting reads by paired-end NGS. Cas3-mediated large mutations were detected by discordant and split read analyses, but Cas9-mediated small mutations were only detected by splitting read analysis. **b** WGS of 293T cells mediated by the CRISPR-Cas3 system targeting *EMX1* on chromosome 2. *P < 0.05, Repetitive Grubbs test. **c** POT sites detected by GGGenome searches. Number of consecutive perfect matches or 32-nt partial matches flanking PAM sequences (AAG, TAG, AAC, GAG, AGG, and ATG) are shown. Every sixth position from the 5′ end of the PAM sequences was regarded as a match. **d** Microarray-based capture sequencing for POT sites selected by GGGenome in **c**. An off-target site of Cas9 targeting the *EMX1* loci showed a high score and a variety of indel mutations. Source data are in the Source Data file.

Whether the type I CRISPR-Cas3 system induces unwanted off-target mutations at nontargeted genomic regions, as seen with the type II CRISPR-Cas9 system, is of great concern^50^. Therefore, we conducted a comprehensive WGS analysis (2 × 150 bp) with 90× coverage in Cas3-mediated 293T cells. To detect structural variations of >1 kb DNA deletions through the human genome (hg38), discordant read pairs and split reads were extracted by SAMtools and LUMPY programs, respectively (Fig. 3a). At the on-target *EMX1* site (chr2: 72,900,001–73,000,000), we observed a top potential DNA degradation (PDD) score of 5.24 in the genome, which was equivalent to 16.1% editing efficiency as estimated by capture sequencing (Fig. 3b). We detected ten genomic regions with >3.5 PDD scores (>10.7% editing efficiency) as potential off-target (POT) sites, but most of the mapped pair-reads were classified as less than 1 kb in distance, in which reads were mapped on SINE repeat elements (Supplementary Fig. 9). PCR analysis of the eleven sites indicated that only the on-target site showed a DNA degradation pattern (Supplementary Fig. 16). Thus, these POT mutations were not mediated by the CRISPR-Cas3 system.

Because WGS analysis did not identify rare mutations (less than 10% editing efficiency) induced by Cas3-mediated off-target effects, we performed capture sequencing of POT sites in the human genome using the GGGenome computer program (https://GGGenome.dbcls.jp/) with two approaches (Fig. 3c). First, we searched for consecutive perfect matches from the 5′ end of the spacer sequences with previously reported PAM sequences: AAG, TAG, AAC, GAG, AGG, and ATG^31,32,36,51^. Every sixth position from the 5′ end of the spacer sequences was regarded as a match, because these nucleotides are not involved in target recognition^32,35–38^. Twenty-eight sites with consecutive 17–21-nt matches for the *EMX1* sequence and 73 sites with consecutive 16–18-nt matches for the *CCR5* sequence were identified as POT sites (Fig. 3c and Supplementary Tables 5 and 6). Next, 32-nt from the 5′ end of the spacer sequences were searched allowing up to 7-nt mismatches, with every sixth position regarded as a match. The search identified 42 and 113 POT sites within 7-nt mismatches for the *EMX1* and *CCR5* sequences, respectively (Supplementary Tables 7 and 8). In contrast, 74 and 50 POT sites within four mismatches were identified for Cas9 recognition (Fig. 3c).

Custom microarray-based capture sequencing for each 10 kb around the POT sites (Fig. 3c and Supplementary Tables 5–8) provided 2200–2700× coverage of the genome sequencing. Split read analysis indicated significantly higher PDD scores for Cas3- and Cas9-mediated on-target loci for *EMX1* and *CCR5* (Fig. 3d). POT sites for *EMX1*-Cas3 and *CCR5*-Cas3 targeting did not have any significant PDD scores, whereas *EMX1*-Cas9 targeting showed a significant score for a POT site harboring a 3-bp mismatch with the spacer sequences. Indeed, we found various Cas9-mediated indel mutations at the off-target site (Fig. 3d). Taken together, WGS and capture-based deep sequencing for POT sites demonstrated high on-target specificity of the CRISPR-Cas3 system, which is comparable or lower than the off-target activity observed for the CRISPR-Cas9 system. However, further evaluation of the off-target effects with more sensitive methods at more target sites in different types of mammalian cells should be investigated.

### Potential therapeutic application of the CRISPR-Cas3 system

To further demonstrate Cas3 nuclease activity in different types of cells, such as induced pluripotent stem cells (iPSCs), we again targeted the *B2M* gene. Because of the lower transfection efficiency of iPSCs, we prepared several types of plasmids that combined multiple Cas proteins with P2A or T2A peptides and selection markers, such as Puromycin resistance, Hygromycin resistance, or mCherry gene (Supplementary Fig. 17a). Two days after the transfection, only the iPSCs were exposed to 50 μg mL^−1^ hygromycin and 0.5 μg ml^−1^ puromycin for 24 h. We then measured cell surface HLA expression by immunofluorescence staining (Supplementary Figs. 17b and 18a). The CRISPR-Cas3 system abrogated 8.6–14% of HLA expression in 293T cells and 1.2–5.9% in iPSCs by biallelic disruption of *B2M*. The *B2M* deletion in iPSCs was confirmed by genomic PCR and Sanger sequencing (Supplementary Fig. 18b). These results indicated that the CRISPR-Cas3 system is efficient at inducing large deletions in human iPSCs.

**Fig. 4:**
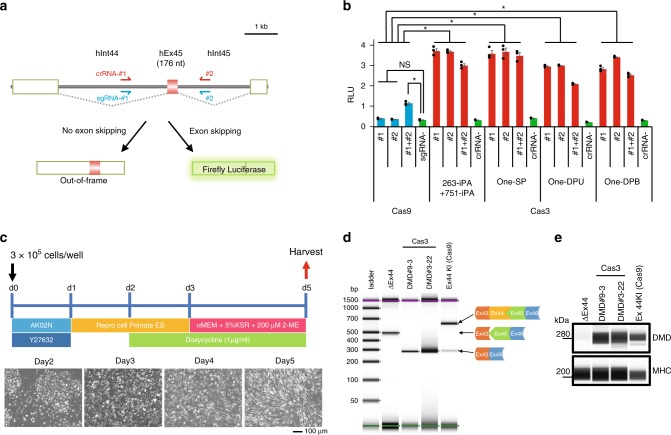
Cas3-mediated *DMD* exon skipping in induced pluripotent stem cells. **a** Schematic of Cas3 (red)- or Cas9 (blue)-mediated *DMD* exon skipping by the luciferase reporter assay. **b** Efficiency of the *DMD* exon skipping in 293 T cells by polycistronic plasmid vectors expressing CRISPR-Cas3 (see Supplementary Fig. 17a). Data are shown as mean ± s.e.m *P < 0.05, ANOVA with post-hoc Tukey test. **c** Scheme of skeletal muscle cell differentiation from iPSCs by doxycycline (Dox)-induced *MYOD1* expression. bar indicates 100 µm. **d** RT-PCR analysis of the *DMD* exon 45 skipping in skeletal muscle cells differentiated from subclones of DMD patient-derived iPSCs treated with Cas3 (#3-22 and #9-3). The iPSC line Ex44 KI is a control line previously generated by the insertion of Exon 44 in front of Exon 45 using SpCas9^52^. **e** The restored DMD protein detected by using Wes ProteinSimple in skeletal muscle cells differentiated from the exon skipping subclones of DMD-iPSCs. The iPSC line Ex44 KI was used as a positive control. Source data are in the Source Data file.

Finally, we sought to use the CRISPR-Cas3 system for therapeutically relevant genome editing by inducing exon skipping in the *dystrophin* (*DMD*) gene. To assess the efficiency of *DMD* exon skipping quantitatively, we used a Firefly luciferase-based exon skipping reporter split by a 4-kb DNA fragment containing intron 44, exon 45, and intron 45 of human *DMD* (Fig. 4a). We tested CRISPR-Cas3 with either crRNA #1 or #2 at intron 44 or intron 45, or CRISPR-Cas9 with sgRNAs #1 and #2 for skipping exon 45 in 293T cells. As expected, single-cut CRISPR-Cas3 showed significantly higher levels of exon skipping than CRISPR-Cas9 with the two sgRNAs (Fig. 4b). Finally, DMD-iPSCs (clone ID: CiRA00111) isolated from a Duchenne muscular dystrophy patient harboring a deletion of exon 44^52^ were targeted for exon 45 skipping by the CRISPR-Cas3 system. After CRISPR-Cas3 treatment and subcloning, several genome-edited iPSC clones were isolated with a deletion at the *DMD* exon 45 locus and detected by PCR genotyping and sequencing (Supplementary Fig. 18c). The genomic correction efficiency of *Dystrophin* in DMD-iPSCs was 7.8% (4 clones out of 51) for crRNA DMD#9 and 14.6% (7 clones out of 48) for DMD#3. No integration of the expression vector was confirmed by flow cytometry for mCherry and EGFP in Cas3-treated bulk iPSCs (Supplementary Fig. 18d) and by qPCR in those clones (Supplementary Fig. 18e). Among the several genome-edited clones, two iPSC clones, DMD#3-22 and DMD#9-3, were differentiated into skeletal muscle cells by doxycycline (Dox)-inducible *MYOD1* expression (Fig. 4c), as previously reported^53^. RT-PCR (Fig. 4d) and western blotting analyses (Fig. 4e) of the differentiated skeletal muscle cells demonstrated successful restoration of the DMD protein by the skipping of exon 45 mediated by the CRISPR-Cas3 system.

## Discussion

In this study, we successfully adapted one of the most evolutionally prevalent Class I type I-E CRISPR-Cas systems for various genome editing applications in human cells. Although the type I system has been studied in prokaryotes and in vitro, little is known about its characteristic and activity in mammalian cells. We demonstrated that Cascade components, Cas3, and 90-nt pre-crRNA, but not 61-nt mat-crRNA, were essential for cleaving plasmid DNA and targeting genomic DNA (Fig. 1c). Codon-optimized Cas effector components with a CAG promoter and bpNLS^29,30^ were used to increase the nuclear concentration in mammalian cells. However, it is not clear why Cas effectors with 61-nt long mat-crRNA showed little or no activity, even though mat-crRNA enhances cleavage activity in vitro^23,24,54^. In the type I system, the processing of pre-crRNA into its mature form with Cascade might be important for functional activity in vivo (Supplementary Fig. 1). During this process, Cas6 endoribonuclease cleaves repeat sequences of the pre-crRNA at specific positions and hold the repeat-derived 3′ handle of the mat-crRNA, which plays a key role in stabilizing the crRNA interaction with Cascade^55^. Several studies reported the in vivo reconstruction of the type I CRISPR system in *E. coli*^25–27^, and a recent paper used pre-crRNA in a eukaryote model, *Saccharomyces cerevisiae*^56^, which support our findings using pre-crRNA in human cells.

While this manuscript was under revisions, another study utilizing Thermobifida fusca derived type I-E CRISPR-Cas3 system also reported unidirectional long-range genomic DNA deletion^13^. The authors found *T. fusca* Cas3 could mediate similar deletion patterns, ranging from a few hundred base pairs to hundreds of kilobases, oriented to the 5′-side of the crRNA target site. The major difference in the methodology used compared with our study was that they utilized ribonucleoprotein (RNP) Cascade complex with mat-crRNA purified from *E. coli*, while we utilized plasmid DNA vectors with pre-crRNA. The transient nature of RNP may lower the risk of off-target mutagenesis, however it could be difficult to purify the RNP complex with crRNA from *E. coli* for every crRNA target^13^. Our plasmid-based system is easily programmable for either individual or multiple target sites as swapping the target sequence or multiplexing of pre-crRNAs is straight forward. With the *T. fusca* Cas3 system, a side-by-side comparison with the Cas9 system to evaluate the risk of off-target mutagenesis is yet to be investigated^13^.

It has been shown that large genomic deletion can also be induced by CRISPR-Cas9 with two sgRNAs that flank the region to be deleted. We demonstrated that *E. coli* Cas3 with just a single crRNA induced large deletions more efficiently than Cas9 (Fig. 2). Cas9 with two sgRNAs may also increase the chance of undesirable off-target mutations as well as unintended reverse mutations between the cutting sites, as shown in Supplementary Fig. 13a. We also demonstrated that Cas3 achieved more efficient genome editing including gene KO and KI than Cas9 from a distance of a few dozen to a few hundred bp upstream of the targeted site, while Cas9 enforced more efficient genome editing at the target site (Fig. 2f, g). Thus, Cas3-mediated genome editing may be useful when targeting specific sequences that are difficult to design onsite sgRNAs, such as sites far from PAM sequences, repetitive sequences, or transposon elements^57,58^.

Regarding target specificity, we used the WGS analysis as an initial screening step, alongside with in silico analysis for listing up the POT sites to be investigated by the target-capture sequencing with higher sequencing depth. Although WGS with 90× coverage alone is not enough to detect rare mutations in the bulk cell population due to the level of sensitivity, custom microarray-based capture sequencing of over 100 POT regions listed by in silico analysis provided more than 2200× coverage of the sequencing depth. By the capture sequencing, we identified various Cas9-mediated indel mutations at the POT site (Fig. 3d), while no prominent off-target mutations were observed with the CRISPR-Cas3 system. Indeed, the longer length of the 27 nt crRNA for target binding in the Cas3 system than the 20 nt gRNA in the Cas9 system might allow stricter target recognition, although we did detect some SSA activity at sites with mismatches in Fig. 1f. In the type I-E CRISPR-Cas system, the Cascade surveillance complex with crRNA initiates R-loop formation, which then recruits Cas3 nuclease/helicase for processively degrading the target DNA^31,32,54,59–62^. Recent cryo-electron microscopy analysis demonstrated that full R-loop formation triggered processive Cas3 DNA cleavage^61^, suggesting more stringent controls for interference. In addition, single molecule studies indicated that Cas3 dissociates from Cascade in a stochastic fashion and travels alone for kb distances^62,63^. Fluorescence resonance energy transfer analysis revealed that Cas3 remains tightly anchored to the Cascade complex while repeatedly reeling in the target DNA^54^. These studies of type I-E CRISPR demonstrate in vitro DNA degradation and replicate the Cas3 cleavage to short DNA fragments with a mean size of 30–100 nt, which is preferential for primed acquisition. However, understanding how Cas3 progressively degrades a long range of plasmid DNA in vivo remains elusive. Our study using type I-E CRISPR in human cells revealed a broad range of DNA degradation from 100 bp–100 kb to deletions of dozens of kb at the target site. Interestingly, the spacer sequences and PAM sequences remain in the absence of small indel mutations. This phenomenon suggests that the CRISPR-Cas3 system repeats DNA degradation several times during the cell culture, which expands the deletion size upstream of the target site.

Unpredictable large Cas3-induced deletions may require caution in genome editing—for example, in gene-dense regions or near regulatory elements. However, we and others observed unintentional deletions of thousands of bp around the target site when mediated by Cas9 (Supplementary Fig. 8b and Kosicki et al.^43^). Although normal cell proliferation and viability were observed after transfection with Cas3 or Cas9 in our experiments (Supplementary Fig. 6b), these large and heterogeneous deletions may induce genome instability and unspecific toxicity in mammalian cells. We believe that Cas3-mediated unidirectional DNA degradation can prevent unpredicted deletions, at least in one direction. In addition, two crRNAs flanking the target region confined the Cas3-mediated deletion size within the targeted region (Fig. 2e). However, controlling the size of the Cas3-mediated DNA deletions requires further work, as it is challenging to engineer the nuclease or helicase domain of Cas3 without abolishing the genome editing activity (Fig. 1d). Recently discovered anti-CRISPR proteins, such as AcrE1^64,65^, which inhibit Cas3 nuclease or helicase activities, may be another option to restrict excessive DNA degradation in mammalian cells.

Finally, we tested the therapeutic potential of Cas3 on DMD. DMD is an intractable genetic disorder caused by loss of the *DMD* gene, which is composed of 79 exons spanning 2 Mb of the genomic region on the X chromosome, where each exon is flanked by long (mean > 25 kb) introns. Based on the long intervals between exons, we hypothesized that DMD exon skipping would make a good proof-of-concept for the long-range deletions by the Cas3 system as potential therapeutic applications. However, several important challenges for the CRISPR-Cas3 system remain: (1) overall it has a lower editing efficiency compared with the CRISPR-Cas9 system; (2) it is difficult to deliver multi-effector components to cells or tissues; and (3) there are concerns about the immunogenicity of several bacterial proteins. In summary, this is the first report utilizing the *E. coli* type I-E CRISPR system as a genome editing tool in mammalian cells. This finding broadens the potential applications of other Class 1 CRISPR systems as new genome editing technologies, such as epigenome editing, base-editing, multi-color fluorescent protein imaging, or tagging any multiple domains at the defined genomic locus. Long-range deletion is also ideal to fight against infectious viruses or pathogenic bacteria, removing noncoding RNAs, miRNA clusters, or regulatory regions in mammalian cells, in which all of them are challenging with current Cas9 system.

## Methods

### Construction of Cas genes and crRNAs

Cas3 and Cascade components Cas5, Cas6, Cas7, Cas8, and Cas11 from *E. coli* K-12 with a bipartite nuclear localization signal (bpNLS)^29,30^ at each 5′ and 3′ end were designed and cloned by gene synthesis (Thermo Fisher Scientific) after being codon-optimized for mammalian cell expression. GeneArt GeneOptimizer (Thermo Fisher Scientific) was used for the codon optimization. These genes were subcloned downstream of the CAG promoter into the pPB-CAG.EBNXN plasmid (gift from the Sanger Institute; http://www.sanger.ac.uk/form/Sanger_CloneRequests) (Addgene ID: 134919, 134920). The Cas3 mutant expression plasmids H74A (dead nickase; dnCas3), K320N (dead helicase; dhCas3), and S483A and T485A double mutant (dead helicase version 2; dh2Cas3), were generated by self-ligation of the PCR product with PrimeSTAR MAX (Takara Bio).

To construct polycistronic Cas expression vectors for iPSCs, mammalian codon-optimized cDNAs of the Cas genes with bpNLS sequences were custom synthesized (GenScript), and three cDNAs were conjugated with P2A or T2A peptide sequences (one for Cas7, Cas5 and Cas8, and another for Cas11, Cas6 and Cas3). These polycistronic cassettes were cloned into *piggyBac* transposon vectors (pPV-EF1α-GW-iPA, pPV-EF1α-GW-iCA or pPV-EF1α-GW-iGA) by the Gateway LR reaction as shown in Supplementary Fig. 17a (Addgene ID: 134922, 134923, 134924, 134925).

For the crRNA expression plasmid, crRNA sequences including two BbsI restriction sites at the spacer region under the U6 promoter were synthesized (GenScript). All targeted sequences are listed in Supplementary Table 9. All crRNA expression plasmids were created by the insertion of 32-bp double-stranded oligonucleotides for the target sequences at the BbsI restriction site (see Supplementary Fig. 3a) (Addgene ID: 134921). Cas9-sgRNA expression plasmid pX330-U6-Chimeric_BB-CBh-hSpCas9 was obtained from Addgene. For the sgRNA design, CRISPRdirect (https://crispr.dbcls.jp/) was used to select unique target sites throughout the human genome^66^. The selected sequences were cloned into the sgRNA scaffold of pX330 according to the protocol of the Feng Zhang lab (http://www.genome-engineering.org/).

The Firefly luciferase reporter plasmid including two BsaI restriction sites was used for the SSA assay as previously reported^67^. Genomic regions at *EMX1* including the target sequence (Supplementary Table 9) were integrated into the BsaI restriction site. The *Renilla* luciferase reference vector was obtained from Promega as pRL-TK. All plasmids were prepared by Midiprep with the PureLink HiPure Plasmid Purification Kit (Thermo Fisher Scientific). Primer sequences for the cloning of all plasmids are listed in Supplementary Table 10.

### Evaluation of DNA cleavage activity

The SSA assay was carried out to detect DNA cleavage activity in mammalian cells as described previously^67^. 293T cells, kindly provided by Professor Takeshi Todo at Osaka University, were cultured in high-glucose Dulbecco’s modified Eagle’s medium (Nacalai Tesque) supplemented with 2 mM l-glutamine and 10% fetal bovine serum at 37 °C with 5% CO_2_. At 24 h after seeding 0.5 × 10^4^ cells in each well of a 96-well plate, 250 ng each of Cas3, Cas5, Cas6, Cas7, Cas8, Cas11, and crRNA expression vectors with 100 ng of SSA reporter plasmid and 60 ng of *Renilla* luciferase reference vector were transfected into 293T cells by using Lipofectamine 2000 and Opti-MEM (Thermo Fisher Scientific) according to the manufacturer’s protocol with minor modifications. At 24 h after transfection, dual luciferase assays were performed using the Dual-Glo luciferase assay system (Promega) according to the manufacturer’s protocol.

### Detection of large deletion mutations in human cells

At 24 h after seeding 5.0 × 10^4^ 293T cells in each well of a 24-well plate, 250 ng each of Cas3, Cas5, Cas6, Cas7, Cas8, Cas11, and crRNA expression vectors were transfected into 293T cells as described above. Two days after the transfection, whole DNA was extracted from collected cells using the Tissue XS Kit (Takara Bio) according to the manufacturer’s protocol. The targeted regions were amplified using Quick Taq HS DyeMix (Toyobo), KOD FX Neo (Toyobo) or Gflex (Takara Bio) and electrophoresed on an agarose gel or Agilent 2200 Tape Station (Agilent Technologies). To detect small indel mutations in the PCR products, T7 endonuclease I (New England Biolabs) or the SURVEYOR Mutation Detection Kit (Integrated DNA Technologies) was used in accordance with the manufacturer’s protocol. For TA cloning, the PCR products were purified using a DNA Clean Up Kit and subcloned into a pCR4Blunt-TOPO plasmid vector (Thermo Fisher Scientific) according to the manufacturer’s protocol. Sequencing analysis was performed using the BigDye Terminator Cycle Sequencing Kit and an ABI PRISM 3130 Genetic Analyzer (Thermo Fisher Scientific).

To detect the ratio of variant mutations, DNA libraries of the PCR amplicons were prepared with a Nextera XT DNA Library Prep Kit or TruSeq Nano DNA Library Prep Kit (Illumina), and amplicon sequencing was performed by using MiSeq (2 × 150 bp) according to the standard procedure at Macrogen. Raw reads from each sample were mapped with human genome assembly hg19 and hg38 by BWA-MEM, and the depth of coverage was extracted by SAMtools. The histogram at on-target regions was visualized by Integrative Genomics Viewer (IGV). The editing efficiency for Cas3-mediated large deletions was estimated by the rate of coverage at the edited locus (300 bp downstream of the AAG-PAM sequences) via NGS of the PCR amplicons. The coverage data was compensated by that of an unedited locus (100 or 300 bp upstream of the AAG-PAM sequences). The editing efficiency of Cas9-mediated small indel mutations around the PAM sequences was calculated by the ratio of the edited/unedited locus via CRISPResso2 (http://crispresso.pinellolab.partners.org/) according to the developers’ protocol^68^.

### Assay for toxicity of the Cas3-mediated large deletion

At 24 h after seeding 5.0 × 10^4^ 293T cells in each well of a 24-well plate, CRISPR-Cas3 or -Cas9 expression plasmids were transfected as described above. Two days after transfection, the total cell number was counted using the Countess Automated Cell Counter (Thermo Fisher Scientific). Subsequently, apoptosis assays were performed with the Annexin V-FITC Apoptosis Detection Kit (Nacalai Tesque) according to the manufacturer’s protocol. FITC-positive cells were detected by FACS Aria IIIu (Becton Dickinson).

### Detection of POT sites

The POT regions for type I-E CRISPR in the human genome assembly GRCh38/hg38 were searched using GGGenome (https://GGGenome.dbcls.jp/) rather than the widely used BLAST^69^, because BLAST may overlook some POT sites^66^. GGGenome quickly searches short nucleotide sequences using a suffix array and FM index on a solid-state drive. The potential PAM sequences were selected (AAG, TAG, AAC, GAG, AGG, and ATG) according to previous studies^31,32,36,51^. We detected lower mismatched regions to 32 bases of the on-target sequence except for multiples of six, because these positions cannot recognize the target site when using the GGGenome web tool. We also detected regions perfectly matched to the 5′ end of the target sequence from the PAM and listed them by the highest number of perfect-match bases. These regions are shown in Supplementary Tables 5–8.

### Deep sequencing for on- and off-target analysis

For WGS, genomic DNA was extracted from transfected 293T cells and sheared using a Covaris ultrasonicator. After the preparation of a DNA library with the TruSeq DNA PCR-Free LT Library Prep Kit (Illumina), genomic sequence analysis was performed by HiSeq X (2 × 150 bp) according to the standard procedure at Takara Bio. Raw reads from each sample were mapped with human genome assembly hg19 or hg38 and transfected-plasmid sequences by BWA-MEM and cleaned by the Trimmomatic program. Discordant read pairs and split reads were extracted by SAMtools and Lumpy-sv, respectively. The total number of discordant reads and split reads on each 100 kb region were counted using Bedtools. After removing regions with split or discordant read counts less than 20, the split score (SS) and discordant score (DS) were calculated as the ratio of split read counts and discordant read counts of the Type I-E sample to those of the control sample, respectively. Then, PDD scores at each locus were calculated by multiplying the SS by the DS. Outliers of the PDD score were detected by repeating the Grubbs test after logarithmic transformation in R. The read distribution at the high PDD locus was detected from the Sam files at the locus extracted by SAMtools.

To enrich the POT regions before sequencing, SureSelectXT custom DNA probes were designed under moderately stringent conditions by SureDesign and were generated by Agilent Technologies. Targeted regions were selected as follows. The probes around the on-target regions at *EMX1* and *CCR5* covered 800 kb upstream to 200 kb downstream of the PAM. Probes around the off-target regions of CRISPR/Cas3 covered 9 kb upstream to 1 kb downstream of the potential PAM. Probes of CRISPR/Cas9 covered 1 kb upstream and downstream of the potential PAM. After preparation of the DNA library with SureSelectXT reagents and the custom probe kit, genomic sequence analysis was performed by using HiSeq 2500 (2 × 150 bp) according to the standard procedure at Takara Bio. Discordant read pairs and split reads were also extracted using the same method as above. The total number of discordant reads or split reads on each 1 kb and 10 kb region were also counted by Bedtools. Off-target effects at the predicted sites were calculated by subtracting split read counts of control samples from those of CRISPR-transfected samples. Mutation patterns at the on-target locus were calculated by CollectInsertSizeMetrics in Picard-tools to detect the size and distribution of the large deletion. Distribution of the >1 kb large deletion was extracted at chr2: 72,133,910–73,133,910 (1 Mb) and chr3: 45,572,920–46,572,920 (1 Mb) for *EMX1* and *CCR5*, respectively. Distributions with a <1 kb deletion were extracted at chr2: 72,932,910–72,933,910 (1 kb) and chr3: 46,371,920–46,372,920 (1 kb) to remove the error reads on other regions. We also calculated a mean and maximum size of the Cas3- and Cas9-mediated deletions from the capture sequencing data. We extracted mapping reads which included a split position within 1 kb both sides of the target sites as the Cas3 or Cas9-madiated deletion.

For Oxford Nanopore sequencing, we PCR amplified the *EMX1* (3.8 kb) and *DMD* (5.4 kb) loci using Quick Taq HS DyeMix with primer sets (Supplementary Table 10) and attached DNA barcodes using a 1D PCR Barcoding Amplicons Kit (SQK-LSK108, Oxford Nanopore). Double-strand DNA molecules (1 μg) were end-repaired and dA tailed using a NEBNext End repair/dA-tailing Module (E7546, NEB), then cleaned by Agencourt AMPure XP beads (Beckman Coulter). Sequencing adapter Mix 1D (AMX1D) was ligated to 0.2 pmoles of the end-prepped DNA and cleaned with Agencourt AMPure XP beads. SpotON flow cell (FLO-MIN106) was set on a MinION and primed with 800 μl of priming buffer (Running Buffer with 384 μl Fuel Mix and 416 μl nuclease-free water) for 5 min. The sequencing library mix was prepared by mixing 35 μl of Running Buffer with Fuel Mix, 25.5 μl of the Library Loading Bead Kit, 2.5 μl of nuclease-free water and 12.0 μl of the DNA samples. The sequencing run was performed using MinKNOW software with real-time base-calling. We checked the sequencing quality using Poretools, and de-multiplexed with Porechop software. For mapping into the reference sequences, we used Minimap2 with the map-ont option or LAST with the last-train score matrix. Mapped reads were converted to BAM format and visualized with IGV software.

### Colony formation containing large CRISPR-mediated deletions

To select for 293T cells containing large CRISPR-mediated deletions, a two-part puromycin gene separated by an artificial large intron was constructed. Briefly, the puromycin gene was divided into the 5′-puro (1–411) and 3′-puro (412–600) portions by inserting an artificial intron containing the acceptor site of the human *BCL2* gene intron 1 and a donor site, resulting in a two-part puromycin gene, which resulted in puromycin-resistant 293T cells. The two-part puromycin gene is driven by the EF1α promoter and was cloned into a *piggyBac* transposon vector containing PGK-Neo-pA^70^, resulting in pPB-PGK-Neo-pA-EF1α-Puro (intron)-pA. Additional sequences containing 7.4 kb of splice acceptor sites from the adenovirus genome, polyA from bovine growth hormone, and the genome of human *EMX1* were cloned into a unique AscI site of the artificial intron, resulting in pPB-PGK-Neo-pA-EF1α-Puro (large intron)-pA. These additional sequences in the intron rendered the puromycin gene inactive.

pPB-PGK-Neo-pA-EF1α-Puro (large intron)-pA was stably integrated into 293T cells by *piggyBac* transposition, followed by G418 selection at 600 μgml^-1^. A Cascade expression vector (pT2-CAG-Cascade-IRES-Bsd) was subsequently and stably integrated into 293T cells carrying pPB-PGK-Neo-pA-EF1α-Puro (large intron)-pA by *Sleeping Beauty* transposition followed by blasticidin selection at 10 μg ml^−1^.

CAG-Cas3-pA and U6-crRNA targeting *EMX1* or pX330 targeting *EMX1* (2 μg each) were transfected into 2 × 10^5^ 293T cells, which were stably integrated with pPB-PGK-Neo-pA-EF1α-Puro (large intron)-pA and pT2-CAG-Cascade-IRES-Bsd using Lipofectamine 2000. At 48 h post transfection, positive selection was started with 0.5 μg ml^−1^ of puromycin and 400 μg ml^−1^ of G418. Approximately 10 days after the selection, the number of colonies was counted.

### Functional gene KO in 293T cells

Reporter 293T cells carrying mCherry-P2A-EGFP were used to detect functional gene KO as previously reported^44^. Briefly, the cells were cultured with 1 µgml^-1^ puromycin in the medium. After seeding in a 24-well plate, the CRISPR-Cas3 or -Cas9 expression plasmid mixture was transfected as described above. Four or five days after transfection, all cells were collected and analyzed using FACS Aria IIIu (Becton Dickinson) and FlowJo v10 (FlowJo LLC) at the Center for Medical Research and Education, Osaka University. mCherry-negative cells were sorted, and total DNA was extracted as described above. Inversion mutations in the genome were detected by PCR amplification and confirmed by sequence analysis. All primer sets for genotyping are listed in Supplementary Table 10.

Functional *SLC35A2* KO was performed as follows. Four microghrams of CAG-Cas3-pA and 2 μg of U6-crRNA recognizing *SLC35A2* or 4 μg of pX330 recognizing *SLC35A2* were transfected into 293T cells, which were integrated with pT2-CAG-Cascade-IRES-Bsd by using Lipofectamine 2000 (Thermo Fisher Scientific). Six days later, the transfected cells were analyzed as previously reported^71^. To analyze the glycosylation profiles of surface proteins, cells were stained by Alexa Fluor 488-conjugated and Alexa Fluor 647-conjugated lectins (1:100) in FACS buffer containing 1 mM CaCl2, 1 mM MnCl2, and 1 mM MgCl2 on ice for 15 min. After washing twice by FACS buffer, cells were analyzed by the BD FACS Canto II. Data were analyzed by the FlowJo software (FlowJo LLC). KO of the X-linked *SLC35A2* gene encoding UDP-galactose transporter resulted in lectin binding, as detected by Alexa Fluor 647-conjugated *G. simplicifolia* lectin II (GS-II, Thermo Fisher).

Functional *B2M* KO was performed as follows. 293T cells were seeded at 1.5 × 10^5^ cells per well of a 24-well plate. After 24 h, the cells were transfected with 1000 ng of one-DPB plasmid for the expressions of Cas3, Cas5, Cas6, Cas7, Cas8, and Cas11 and 1000 ng of crRNA plasmid or 1000 ng of pPV-EF1α-Cas9-iPA and 1000 ng of sgRNA expression plasmid (or 500 ng each for two sgRNAs) using Lipofectamine 2000 according to the supplier’s recommendations. After the transfection, the cells were maintained for 1 week without any selection. Then the cells were treated with interferon-γ for two days, stained with mouse anti-human HLA-A2 antibody (Becton Dickinson #740082) diluted 100 times in chilled 2% FBS/PBS (–) for 20 min, and analyzed with a BD FACS Aria II (Becton Dickinson).

### KI detection in 293T cells

Reporter 293T cells carrying a copy of mCherry-P2A-EGFP c321C>G were also used for the detection of nucleotide substitution as previously reported^44^. After seeding in a 24-well plate, the CRISPR-Cas3 or CRISPR-Cas9 expression-plasmid mixtures were transfected as described above. To induce nucleotide substitution, 500 ng of the GFP donor plasmids with 1 kb homology arms carrying the wild-type allele of GFP were co-transfected (Supplementary Table 11). Four or five days after the transfection, all cells were collected and analyzed using FACS Aria IIIu (Becton Dickinson). GFP-positive cells were sorted, and total DNA was extracted as described above. Nucleotide substitution in the genome was detected by PCR amplification with HiDi DNA polymerase (myPOLS Biotec) and confirmed by sequence analysis. All primer sets for the genotyping are listed in Supplementary Table 10.

For the KI of gene cassettes, 100 ng of donor long ssDNAs consisting of an EF1α promoter and a puromycin resistance gene with 1 kb homology arms at both ends of the *EMX1* loci were co-transfected with CRISPR-Cas3 expression plasmids as described above. At 24 h post transfection, the cells were harvested and transferred to a 10-cm dish. The following day, puromycin (1 µg ml^−1^) was added to the culture medium, and the cells were maintained for 11 days. To evaluate the correct integration by homology directed repair, junction PCR amplicons were designed for the *EMX1* loci. Then, 1 × 10^5^ cells were lysed in 30 µl lysis buffer with 3 mgml^-1^ proteinase K, and 10 µl of the lysate was used as a template for PCR.

### B2M KO in human iPSCs

The human iPSC line 1383D2 was reprogrammed from the peripheral blood cells of a healthy male donor (CTL-CP1 LP_53, Donor #40, Cellular Technology Ltd) by episomal vectors and was cultured under feeder-free, xeno-free StemFit AK02N media (Ajinomoto) with Laminin-511 E8 (Nippi) coating^72^. Before inoculating the cells, the plates were coated with 0.25–0.35 µg cm^−2^ of Laminin and incubated for 2 h to overnight in a 37 °C CO_2_ incubator. For passaging the cells, iPS cells were dissociated into single cells by 0.5 × TrypLE Select [1 × TrypLE Select (Thermo Fisher) diluted 1: 1 with 0.5 mM EDTA/PBS] for 10 min at 37 °C. 10 μM of Y-27632 (TOCRIS) was supplied to the culture medium on the day of and day after passaging. The iPSCs were seeded at 5 × 10^5^ cells per well of a 12-well plate. After 24 h, the cells were transfected with 1000 ng of CRISPR-Cas3 expression vector (or 500 ng each for two CRISPR-Cas3 expression vectors) and 500 ng of crRNA expression plasmids using Lipofectamine Stem (Thermo Fisher) according to the supplier’s protocol. After 24 h, the cells were transiently selected with 0.5 µg ml^−1^ puromycin and/or 50 µg ml^−1^ hygromycin for 24 h and subsequently cultured for 1 week. Then, the cells were treated with interferon-γ for 2 days, stained with mouse anti-human HLA-A2 antibody (Becton Dickinson #740082) diluted 50 times in chilled 2% FBS/PBS (−) for 20 min, and analyzed. The HLA-A2 negative population was sorted by using a BD FACS Aria II (Becton Dickinson).

### DMD exon skipping assay

In a 96-well plate format, 100 ng of pPV-EF1α-Cas7-Cas5-Cas8-iPA, pPV-EF1α-Cas11-Cas6-Cas3-iPA and crRNA expression plasmids were co-transfected with 100 ng of the luciferase-based exon skipping reporter plasmid pPV-EF1α-Luc2(V323I)-hDMD-Ex45[+](4 kb)-iPA and 20 ng of *Renilla* luciferase plasmid phRL-TK as a transfection control. For the SpCas9 control, 200 ng of pPV-EF1α-Cas9-iPA and 100 ng of sgRNA (or 50 ng each for two sgRNAs) expression plasmids were used. These plasmids were diluted in 25 µl of Opti-MEM I Reduced Serum Medium (Thermo Fisher Scientific), mixed with 1 µl of Lipofectamine 2000, diluted in 25 µl of Opti-MEM Reduced Serum Medium and incubated for 30 min at room temperature in each well of a 96-well plate. 293T cells were dissociated by trypsin-EDTA and seeded into each well at 6 × 10^4^ cells per 100 µl per well. After 48 h cultivation, luciferase activity was measured using a Dual-Glo Luciferase Assay Kit (Promega) and Envision 2104 (Perkin Elmer) plate reader following the manufacturer’s instructions. Firefly luciferase activity was normalized to *Renilla* luciferase activity.

### DMD exon 45-disrupted iPSCs and muscle cell differentiation

DMD-iPSCs (clone ID: CiRA00111) derived from a DMD patient with a deletion of exon 44^52^ were transduced with a Dox-inducible *MYOD1* expression vector for skeletal muscle differentiation^53^. To restore DMD protein expression by skipping exon 45, expression vectors of CRISPR-Cas3 genes and *DMD* exon 45-targeting crRNAs were transfected into the DMD-iPSCs. Briefly, one day before transfection, DMD-iPSCs were seeded at 5 × 10^5^ cells per well of a 12-well plate and transfected with 300 ng of pPV-EF1α-Cas7-Cas5-Cas8-iCA (IRES-mCherry-pA), pPV-EF1α-Cas11-Cas6-Cas3-iGA (IRES-EGFP-pA) and crRNA expression plasmids by using Lipofectamine Stem according to the manufacturer’s protocol. Cells were cultured for 3 days and the EGFP and mCherry double positive population was sorted for by BD FACS Aria II (Becton Dickinson) to enrich the transfected cells. After subcloning followed by PCR genotyping, genome-edited iPSC clones with *DMD* exon 45 deletion were isolated and used for further skeletal muscle cell differentiation experiments. To induce skeletal muscle differentiation, 3.0 × 10^5^ cells of iPSCs were seeded on Matrigel-coated plates with Y23632 supplied StemFit AK02N media. After 24 h, culture media were changed to Primate ES Cell Medium (Reprocell) and the cells were cultured for another 1 day. Then 1 μgml^-1^ of Dox (LKT laboratories) was added to induce *MYOD1* expression. From 3 days post seeding, culture media were changed to αMEM (Nacalai Tesque) with 5% of KSR (Thermo), 200 µM of 2-mercaptoethenol (Thermo), and 1 µgml^-1^ of Dox and the cells were cultured for further differentiation^73,74^.

### RT-PCR for detection of DMD exon skipping

Total RNA was purified from cells using a NucleoSpin RNA Kit (Takara Bio), and cDNA was synthesized using ReverTra Ace qPCR RT Master Mix (Toyobo). Then, corresponding *DMD* exons were amplified by PCR with PrimeSTAR GXL DNA Polymerase (Takara Bio), and the size of the PCR products was assessed by an Agilent 2200 Tape Station (Agilent Technologies). Primer sequences are listed in Supplementary Table 10.

### Detection of DMD protein by western blotting

Cells were lysed with RIPA buffer (Thermo Fisher Scientific) supplemented with cOmplete Protease Inhibitor Cocktail (Roche) and 5 mM EDTA. The total amount of lysate protein was quantified by the Pierce BCA Protein Assay Kit (Thermo Fisher Scientific). DMD and myosin heavy chain (MHC) were detected using a Wes™ Simple Western system (ProteinSimple) with 1.25 µg of sample loading. The primary antibodies used were anti-Dystrophin (Abcam, ab15277, diluted 50-fold) and anti-MHC (R&D Systems, MAB4470, diluted 25-fold). The secondary antibodies used were anti-rabbit IgG HRP-linked antibody (ProteinSimple, 042–206) and anti-mouse IgG HRP-linked antibody (ProteinSimple, 042-205), respectively.

### Reporting summary

Further information on research design is available in the Nature Research Reporting Summary linked to this article.

## Supplementary information

Supplementary Information

Reporting Summary

Peer Review File

## Data Availability

The data that support the findings of this study are available from the corresponding authors upon reasonable request. WGS data reported are available in the DDBJ Sequenced Read Archive under the accession numbers DRA008717 and DRA008718. The source data underlying Figs. 1, 2, 3, 4, and Supplementary Figs 10a,d, 11, 12a, 13b and 18e are provided as a Source Data file. The plasmids pCAG-All-in-one-hCascade (Addgene ID: 134919), pPB-CAG-hCas3 (ID: 134920), pBS-U6-crRNA-empty (ID: 134921), pPV-EF1a-2xNLS-Cas7-Cas5-Cse1-iCA (ID: 134922), pPV-EF1a-2xNLS-Cse2-Cas6-Cas3-iCA (ID: 134923), pPV-EF1a-2xNLS-Cas7-Cas5-Cse1-iPA (ID: 134924) and pPV-EF1a-2xNLS-Cse2-Cas6-Cas3-iPA (ID: 134925) are available from Addgene.

## Source Data

Source Data
