# Supplementary material for: CRISPR-Cas3 induces broad and unidirectional genome editing in human cells
Source: Nat Commun. 2019 Dec 6;10:5302. doi: 10.1038/s41467-019-13226-x (PMC6897959; doi:10.1038/s41467-019-13226-x)
Supplement: Supplementary file 1 — Supplementary Information [file 41467_2019_13226_MOESM1_ESM.pdf]

CRISPR-Cas3 induces broad and unidirectional genome editing in human cells

Morisaka et al.

Supplementary Information

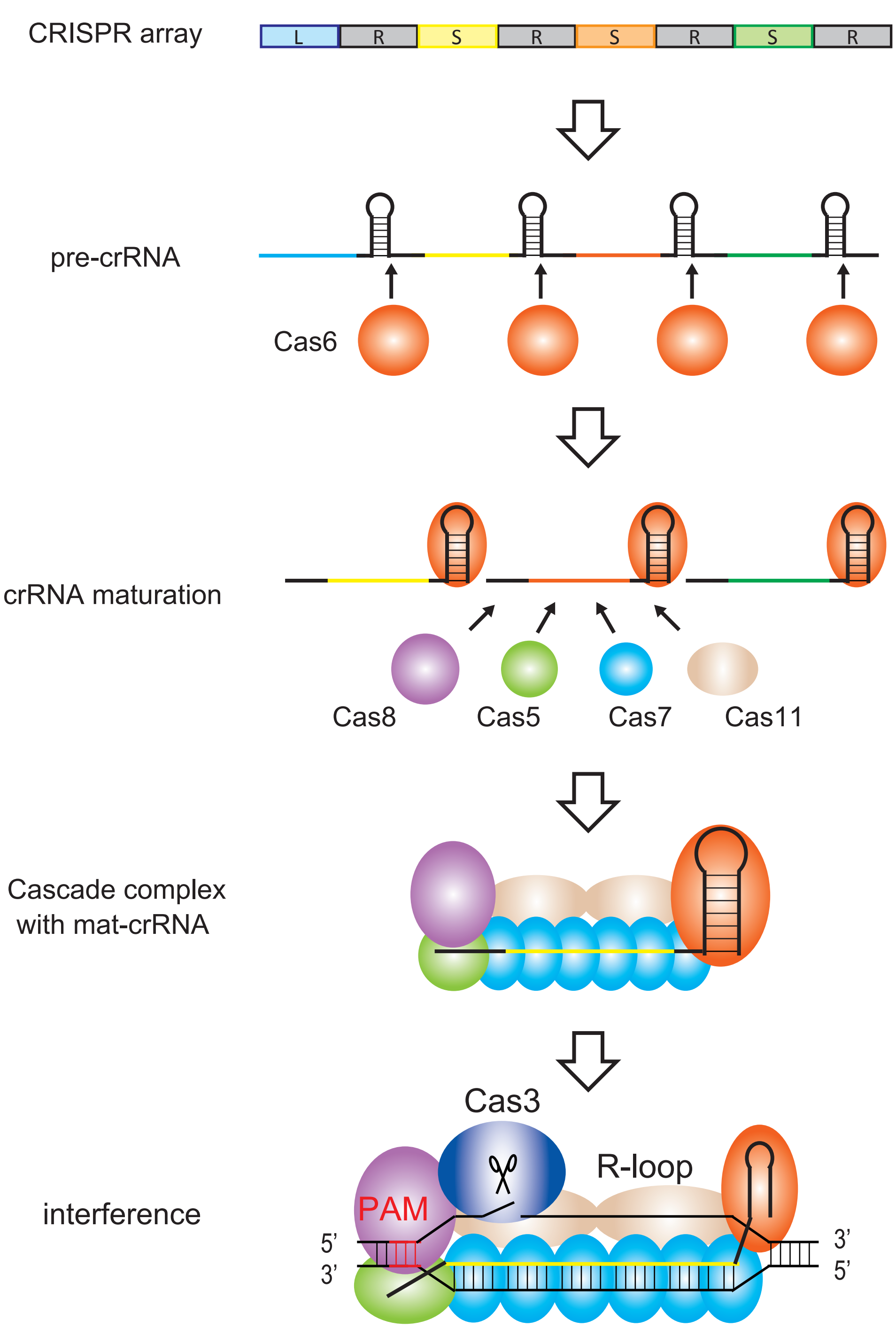

Supplementary Figure 1. crRNA maturation and interference pathways in the type I-E CRISPR-Cas system. A long single precursor crRNA (pre-crRNA) is transcribed with a leader (L: blue) that includes a repeat (R: black)-spacer (S: colors) from the CRISPR array. The pre-crRNA is cleaved by Cas6 endoribonuclease either alone or in association with other Cas proteins into mature crRNAs (mat-crRNAs), which included 8 nt of the 5 ′ handle and 21 nt of the 3 ′ hairpin with the spacer sequences (yellow). Cas6 holds the repeat-derived 3 ′ handle, Cas5 binds the 5 ′ handle, Cas7 forms the backbone, and Cas11 forms the belly of Cascade, which stabilizes the crRNA interaction with Cascade {Hochstrasser, 2015 #93}. In the type I system, the processing of pre-crRNA into mat-crRNA forms the complex with Cascade and is important for functional activity in vivo {Nam, 2012 #153}.

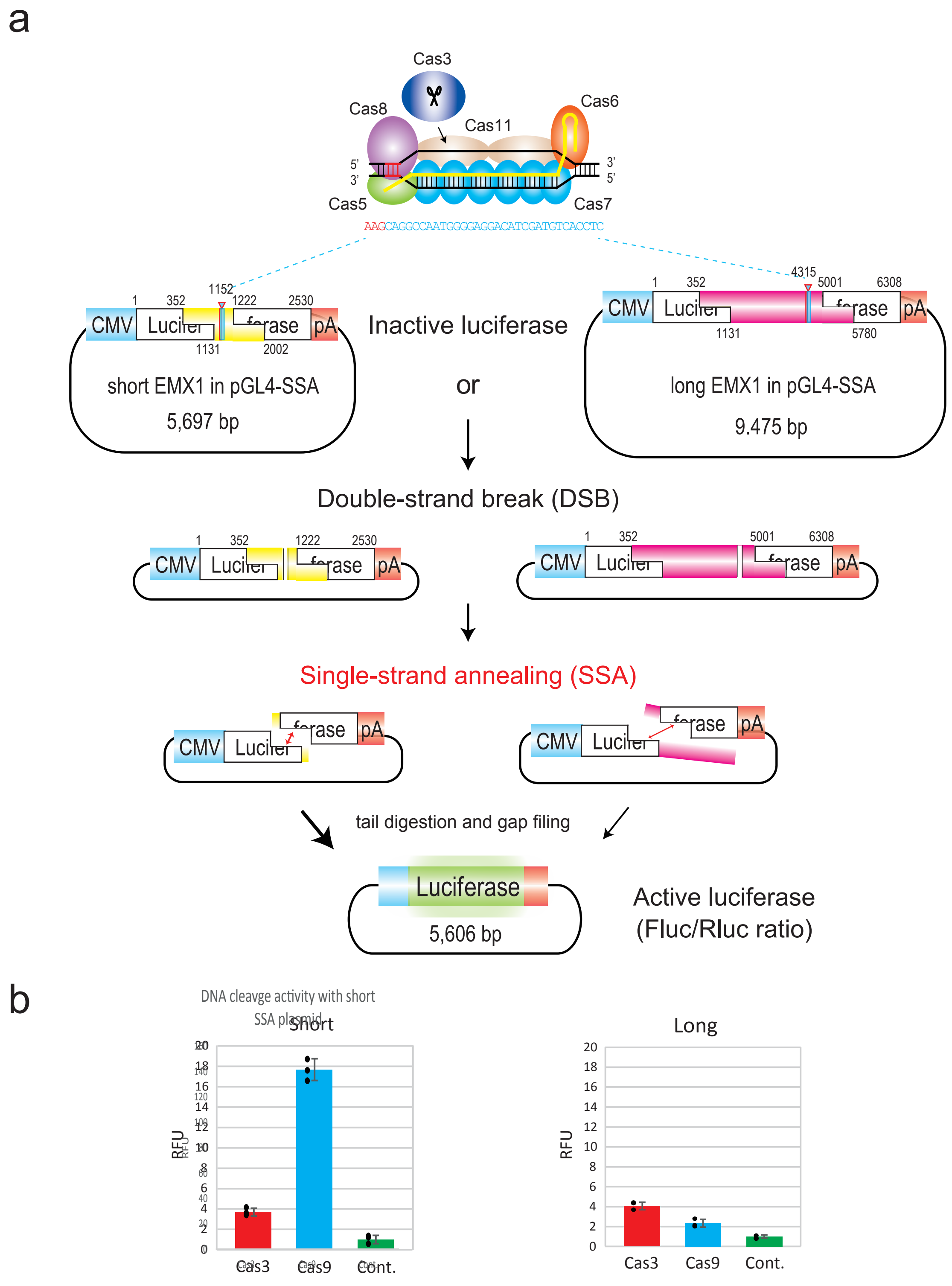

Supplementary Figure 2. The luciferase-based single-strand annealing (SSA) recombination assay. a, SSchematic of the SSA assay used to evaluate the activity of the type I CRISPR-Cas system in 293T cells. A short 91-bp interspaced sequence (yellow) or a long 3.8-kbp sequence (pink), including a 32 nt spacer (blue) with the 5 ′ AAG PAM (red), was integrated between the split luciferase sequence (pGL4-SSA:Addgene #42962). Introduction of a double-strand break (DSB) at the target site by the CRISPR-Cas3 or -Cas9 system produces single-strand DNAs. Then, complementary sequences anneal each other by SSA, followed by digestion of the single stranded tail and gap filling, resulting in active luciferase gene. **b,** Both short and long pGL4-SSA plasmids indicate SSA activity mediated with type I CRISPR (Cas3) but not with type II CRISPR (Cas9) for long pGL4-SSA plasmids. Data are presented as mean ± SD.

a

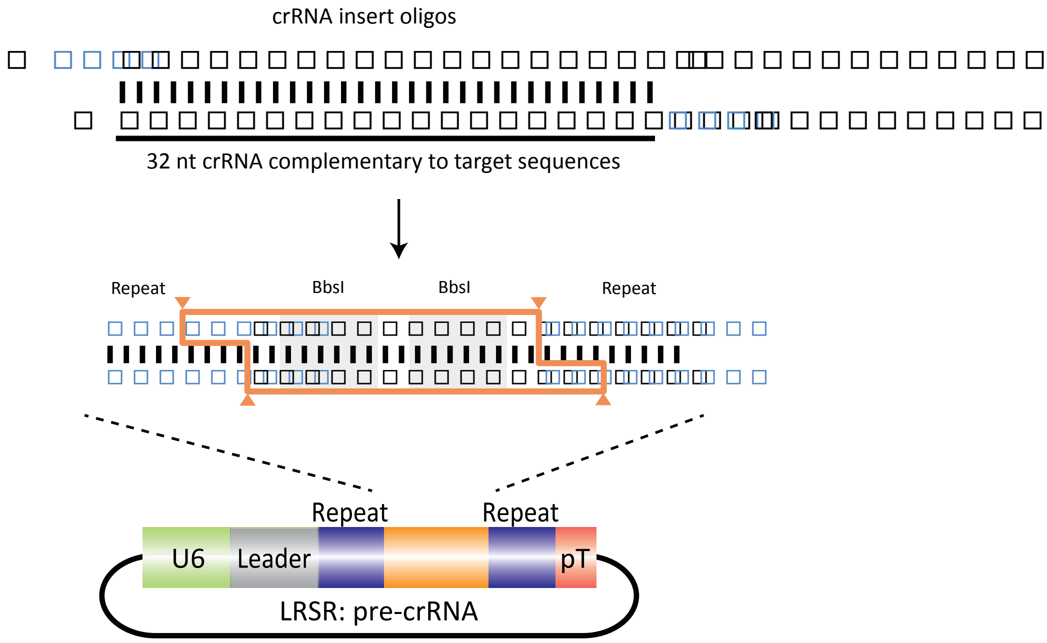

b

>LRSR: pre-crRNA

TTCCCATGATTCCTTCATATTTGCATATACGATACAAGGCTGTTAGAGAGATAATTGGAATT
AATTTGACTGTAAACACAAAGATATTAGTACAAAATACGTGACGTAGAAAGTAATAATTTC
TTGGGTAGTTTGCAGTTTTAAAATTATGTTTTAAAATGGACTATCATATGCTTACCGTAACT
TGAAAGTATTTCGATTTCTTGGCTTTATATATCTTGTGGAAAGGACGTGGATGTGTTGTTTG
TGTGATACTATAAAGTTGGTAGATTGTGACTGGCTTAAAAAATCATTAATTAATAATAGGTT
ATGTTTAGAGTGTTCCCCGCGCCAGCGGGGATAAACCGCAGGCCAATGGGGAGGACATC
GATGTCACCTCGTGTTCCCCGCGCCAGCGGGGATAAACCGTTTTTT

>RSR: pre-crRNA

TTCCCATGATTCCTTCATATTTGCATATACGATACAAGGCTGTTAGAGAGATAATTGGAATT
AATTTGACTGTAAACACAAAGATATTAGTACAAAATACGTGACGTAGAAAGTAATAATTTC
TTGGGTAGTTTGCAGTTTTAAAATTATGTTTTAAAATGGACTATCATATGCTTACCGTAACT
TGAAAGTATTTCGATTTCTTGGCTTTATATATCTTGTGGAAAGGACGGTGTTCCCCGCGCC
AGCGGGGATAAACCGCAGGCCAATGGGGAGGACATCGATGTCACCTCGTGTTCCCCGCG
CCAGCGGGGATAAACCGTTTTTT

>SR: mature-crRNA

TTCCCATGATTCCTTCATATTTGCATATACGATACAAGGCTGTTAGAGAGATAATTGGAATT
AATTTGACTGTAAACACAAAGATATTAGTACAAAATACGTGACGTAGAAAGTAATAATTTC
TTGGGTAGTTTGCAGTTTTAAAATTATGTTTTAAAATGGACTATCATATGCTTACCGTAACT
TGAAAGTATTTCGATTTCTTGGCTTTATATATCTTGTGGAAAGGACGGATAAACCGCAGGC
CAATGGGGAGGACATCGATGTCACCTCGTGTTCCCCGCGCCAGCGGGGTTTTTT

Supplementary Figure 3. Plasmids containing pre-crRNA and mat-crRNA. a, Construct of an expression plasmid for the LRSR pre-crRNA. Annealed oligonucleotides with each ligation adapter at the 5′ end (Top: 5′ -ACCG-3′ , Bottom: 5′ -ACAC-3′) are inserted between two BbsI sites downstream of the U6 promoter. The human U6 promoter expresses a leader-repeat-spacer-repeat array from the LRSR pre-crRNA plasmids. **b, P**lasmid sequences of the pre-crRNA (pLRSR and pRSR) and mat-crRNA (pSR). Green: U6 promoter, Red: leader sequences, Blue: repeat sequences, Orange: spacer sequences targeting the *EMX1* locus. Degradation was mediated by CRISPR-Cas3 with the pre-crRNA plasmids (LRSR or RSR), but not with the mat-crRNA (SR).

Type I-E (*Escherichia coli*)

>Type I-E_bpNLS-Cas3-bpNLS (2,784 bp)

[illegible]

>Type I-E_bpNLS-Cse1-bpNLS (1,626 bp)

[illegible]

>Type I-E_bpNLS-Cse2-bpNLS (600 bp)

[illegible]

>Type I-E_bpNLS-Cas5-bpNLS (792 bp)

[illegible]

>Type I-E_bpNLS-Cas6-bpNLS (717 bp)

[illegible]

>Type I-E_bpNLS-Cas7-bpNLS (1,209 bp)

[illegible]

Type I-F (*Shewanella putrefaciens*)

>Type I-F_bpNLS-Cas2-3-bpNLS (3,054 bp)

[illegible]

>Type I-F_bpNLS-Cas5fv-bpNLS (1,065 bp)

[illegible]

>Type I-F_bpNLS-Cas6f-bpNLS (669 bp)

[illegible]

>Type I-F_bpNLS-Cas7fv-bpNLS (1,128 bp)

[illegible]

Type I-G (*Methanosarcina barkeri*)

>Type I-G_bpNLS-Cas3-bpNLS (2,343 bp)

[illegible]

>Type I-G_bpNLS-Cst1-bpNLS (1,488 bp)

[illegible]

>Type I-G_bpNLS-Cst2-bpNLS (1,119 bp)

[illegible]

>Type I-G_bpNLS-Cas5t-bpNLS (741 bp)

[illegible]

>Type I-G_bpNLS-Cas6-bpNLS (858 bp)

[illegible]

Type II-A (*Streptococcus pyogenes*)

>px330-Cas9 (4,272 bp)

[illegible]

Supplementary Figure 4. Plasmid sequences of the type I-E, type I-F, type I-G, and type II-A CRISPR-Cas system. Type I-E: Cas3, Cas5-8, Cas11 of *Escherichia coli,* type I-F: Cas3 and Cas5-7 of *Shewanella putrefaciens* and type I-G: Cas3 and Cas5-8 of *Methanosarcina barkeri,* type II-A: Cas9 of *Streptococcus pyogenes.* Target sequences of the CRISPR-Cas systems are shown in **Supplementary Table 1**.

a

EMX1

CTGAGTGTTGAGGCCCCAGTGGCTGCTCTGGGGGCCTCCTGAGTTTCTCATCTGTGCCCCTCCCTCC
CTGGCCCAGGTGAAGGTGTGGTTCCAGAACCGGAGGACAAAGTACAAACGGCAGAAGCTGGAGGA
GGAAGGGCCTGAGTCCGAGCAGAAGAAGAAGGGCTCCCATCACATCAACCGGTGGCGCATTGCCAC
GAAGCAGGCCAATGGGGAGGACATCGATGTCACCTCCAATGACTAGGGTGGGCAACCACAAACCCA
CGAGGGCAGAGTGCTGCTTGCTGCTGGCCAGGCCCCTGCGTGGGCCCAAGCTGGACTCTGGCCACT
CCCTGGCCAGGCTTTGGGGAGGCCTGGAGTCATGGCCCCACAGGGCTTGAAGCCCGGGGCCGCCAT
TGACAGAGGGACAAGCAATGGGCTGGCTGAGGCCTGGGACCACTTGGCCTTCTCCTCGGAGAGCCT
GCCTGCCTGGGC

CCR5

GACATCTATGTAGGCAATTAAAAACCTATTGATGTATAAAACAGTTTGCATTCATGGAGGGCAACTA
AATACATTCTAGGACTTTATAAAAGATCACTTTTTATTTATGCACAGGGTGGAACAAGATGGATTAT
CAAGTGTCAAGTCCAATCTATGACATCAATTATTATACATCGGAGCCCTGCCAAAAAATCAATGTGA
AGCAAATCGCAGCCCGCCTCCTGCCTCCGCTCTACTCACTGGTGTTCATCTTTGGTTTTGTGGGCAA
CATGCTGGTCATCCTCATCCTGATAAACTGCAAAAGGCTGAAGAGCATGACTGACATCTACCTGCTC
AACCTGGCCATCTCTGACCTGTTTTTCCTTCTTACTGTCCCCTTCTG

b

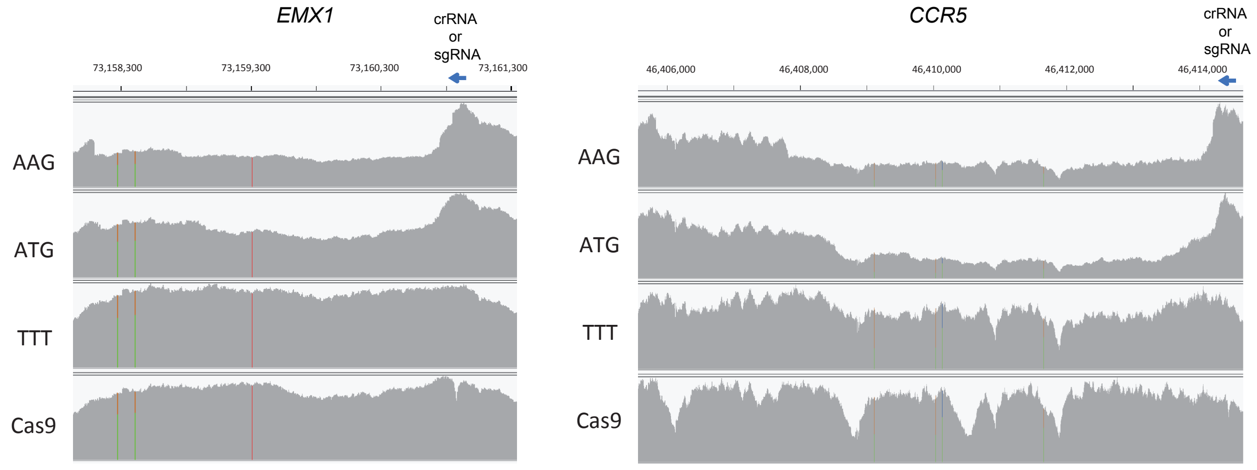

Supplementary Figure 5. CRISPR-Cas3 targeting for *EMX1* and *CCR5* loci. a, crRNAs were designed for various PAM sites (AAG (red), ATG (blue), and TTT (yellow)) for type I Cas3, and sgRNAs were designed for NGG (green) for type II Cas9. **b,** NGS sequencing of PCR amplicons for the 3.7 kb *EMX1* and 9.7 kb *CCR5* target region. Cas3-treated samples (AAG and ATG, but not TTT) showed a wider range of genomic deletions upstream of the target site of crRNA. Cas9-treated samples showed small indels at the target site of gRNA. Experimental observations verified that Illumina short-read sequencing is problematic for complex and repeat-rich genomic regions.

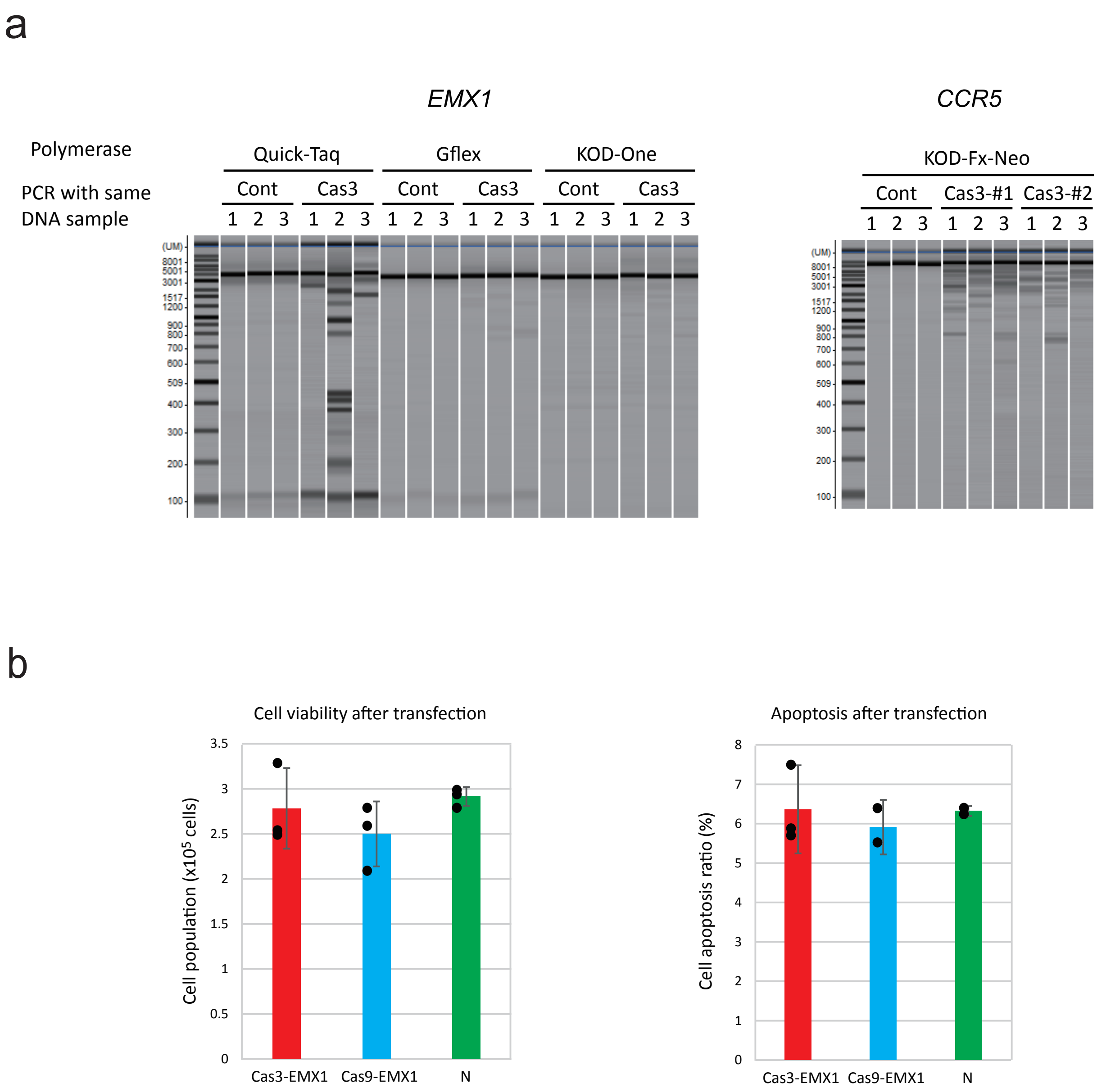

Supplementary Figure 6. Cas3-mediated DNA degradation for *EMX1* and *CCR5* loci. a, Repetitive PCR (No.1-3) with distinct Taq polymerases (Quick-Taq, Gflex, KOD-One, and KOD-Fx-Neo) indicates different banding patterns or smeared PCR products. **b,** Measurements of cell proliferation and apoptosis after transfection of the CRISPR-Cas systems. Data are presented as mean ± SD.

a

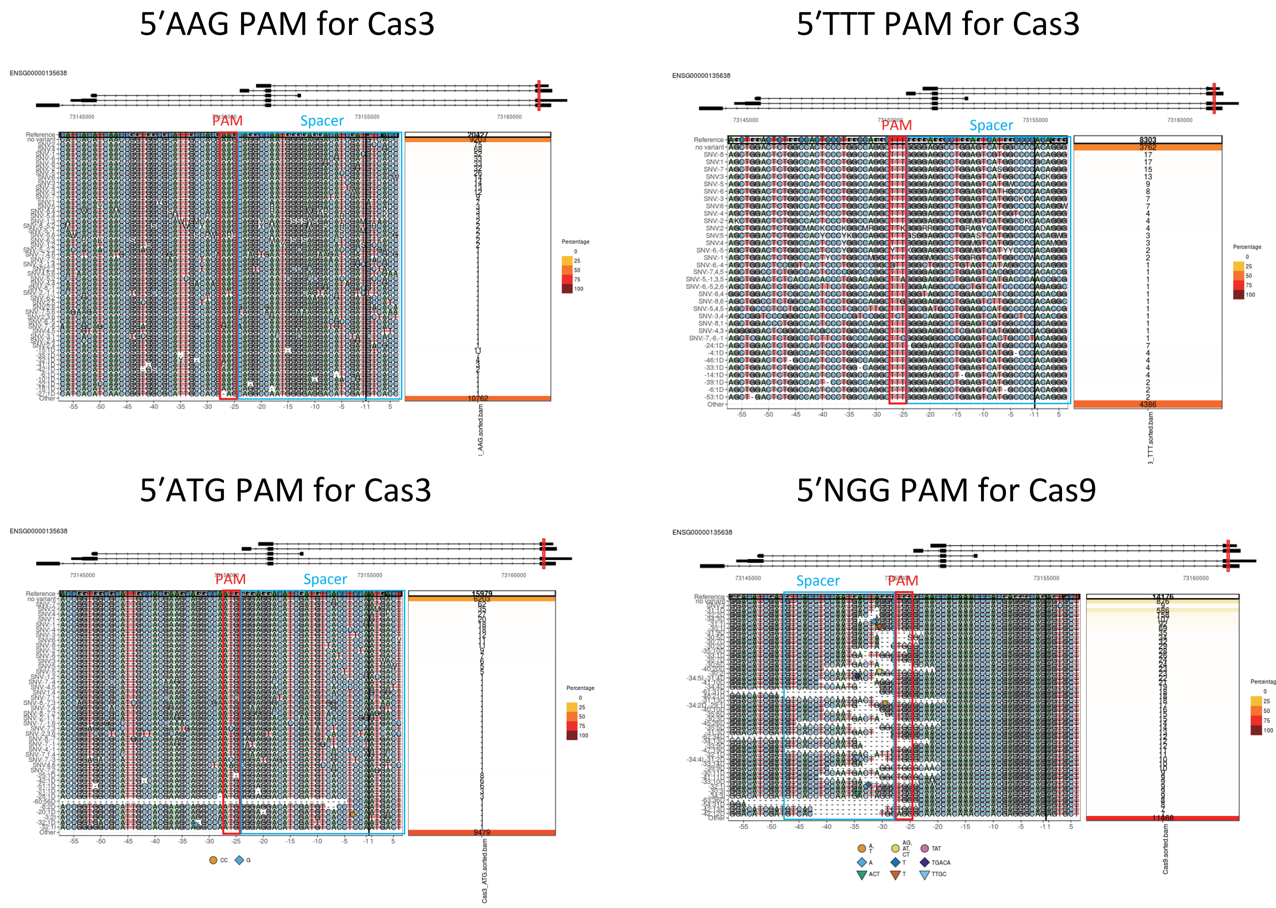

b

Targeted locus		PAM	Read counts of wild allele	Read counts of SNV detection	SNV/Wild ratio	Read counts of indel mutations	Indel/Wild ratio
EMX1	I-E (Cas3)	AAG	9203	430	0.047	32	0.0035
		ATG	6203	265	0.043	32	0.0052
		TTT	3762	126	0.033	29	0.0077
	Cas9	NGG	876	38	0.043	**1794**	**2.05**
	Cont.	-	6406	300	0.047	23	0.0036
CCR5	I-E (Cas3)	AAG	23341	271	0.012	11	0.00047
		ATG	22877	311	0.014	54	0.0024
		TTT	16995	248	0.015	14	0.00082
	Cas9	NGG	5137	74	0.014	**7426**	**1.45**
	Cont.	-	13915	137	0.010	12	0.00086

Supplementary Figure 7. NGS data of PCR amplicons analyzed by CrispRVariantsLite. a, To show small indel mutation patterns around PAM sequences at the *EMX1* locus, CrispRVariantsLite (imlspenticton.uzh.ch:3838/CrispRVariantsLite/) is used for sequence alignment. Various indels were observed with the CRISPR-Cas9 target (5′ NGG PAM), but no indels were observed with the CRISPR-Cas3 targets (5′AAG PAM, 5′ATG PAM, and 5′TTT PAM). **b,** Sequence read count data for wild-type alleles (wild), single nucleotide variants (SNV), and small indel mutations (indel) counted by CrispRVariantsLite. Cas9 induces significantly higher numbers of indel mutations than Cas3.

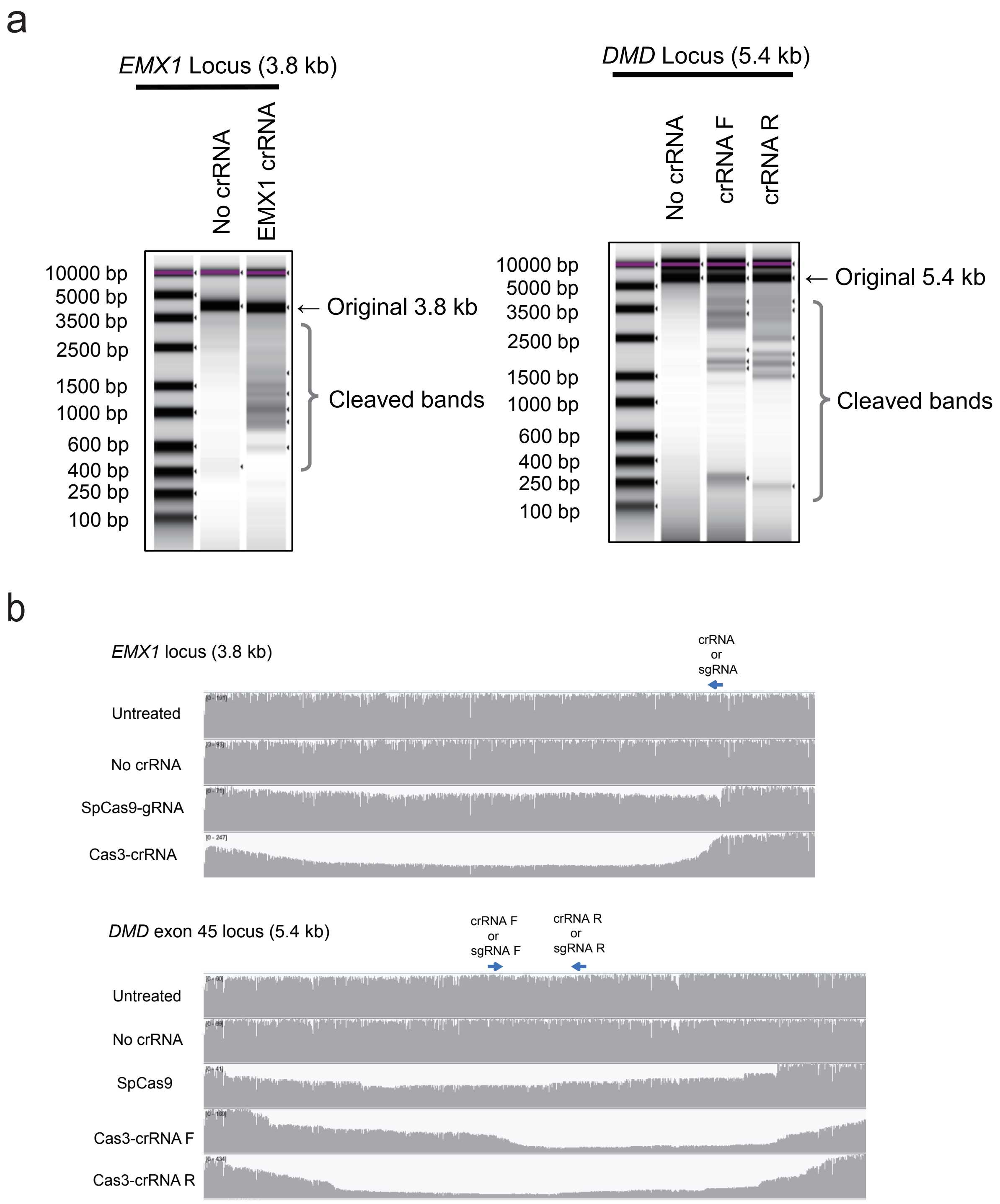

Supplementary Figure 8. Deletion analysis with the MinION Nanopore sequencer for Cas3-mediated *EMX1* and *DMD* genes in 293T cells. a, Electrophoresis of the PCR products at the *EMX1* and *DMD* target loci on an Agilent 2200 Tape Station. Smaller bands than the original PCR product (3.8 kb for *EMX1* and 5.4 kb for *DMD*) indicate broad DNA deletion by Cas3. **b,** The PCR products of the target regions were sequenced by a Nanopore long-read sequencer, and mapped read depths are indicated. Untreated PCR products show uniform coverage throughout the amplified region, whereas Cas3/crRNA-treated samples show a wide range of genomic deletions at the crRNA target site.

a

Region	Split reads 1-E	Split reads Cont	Discordant reads 1-E	Discordant reads Cont	PDD Score
chr1:29800001-29900000	73	31	81	54	3.53
chr2:72900001-73000000	77	32	122	56	5.24
chr3:22100001-22200000	60	25	116	79	3.52
chr3:28200001-28300000	48	24	68	36	3.78
chr5:131400001-131500000	40	22	149	75	3.61
chr6:143200001-143300000	54	28	112	61	3.54
chr7:54100001-54200000	58	22	91	53	4.53
chr10:84500001-84600000	57	22	166	102	4.22
chr18:48700001-48800000	65	28	52	25	4.83
chr18:74600001-74700000	31	21	67	28	3.53
chrX:2700001-2800000	62	29	108	63	3.67

b

		Distance between paired reads (nt)			
	translocation	<499	500-999	1000-9999	>10000
chr1:29800001-29900000	59	68	23	0	4
chr2:72900001-73000000	56	45	25	43	30
chr3:22100001-22200000	116	21	28	0	10
chr3:28200001-28300000	48	49	17	0	2
chr5:131400001-131500000	135	22	29	0	3
chr6:143200001-143300000	101	24	32	0	6
chr7:54100001-54200000	83	42	12	2	10
chr10:84500001-84600000	146	50	24	0	3
chr18:48700001-48800000	38	63	16	0	0
chr18:74600001-74700000	48	25	22	2	1
chrX:2700001-2800000	122	27	14	0	7

Supplementary Figure 9. Potential off-target (POT) regions identified by whole genome sequencing. a, Eleven chromosomal regions including the on-target region (yellow) were identified with potential DNA degradation (PDD) scores >3.5. **b,** Among the eleven regions, only the on-target region on chr 2 included many >1 kb DNA deletions.

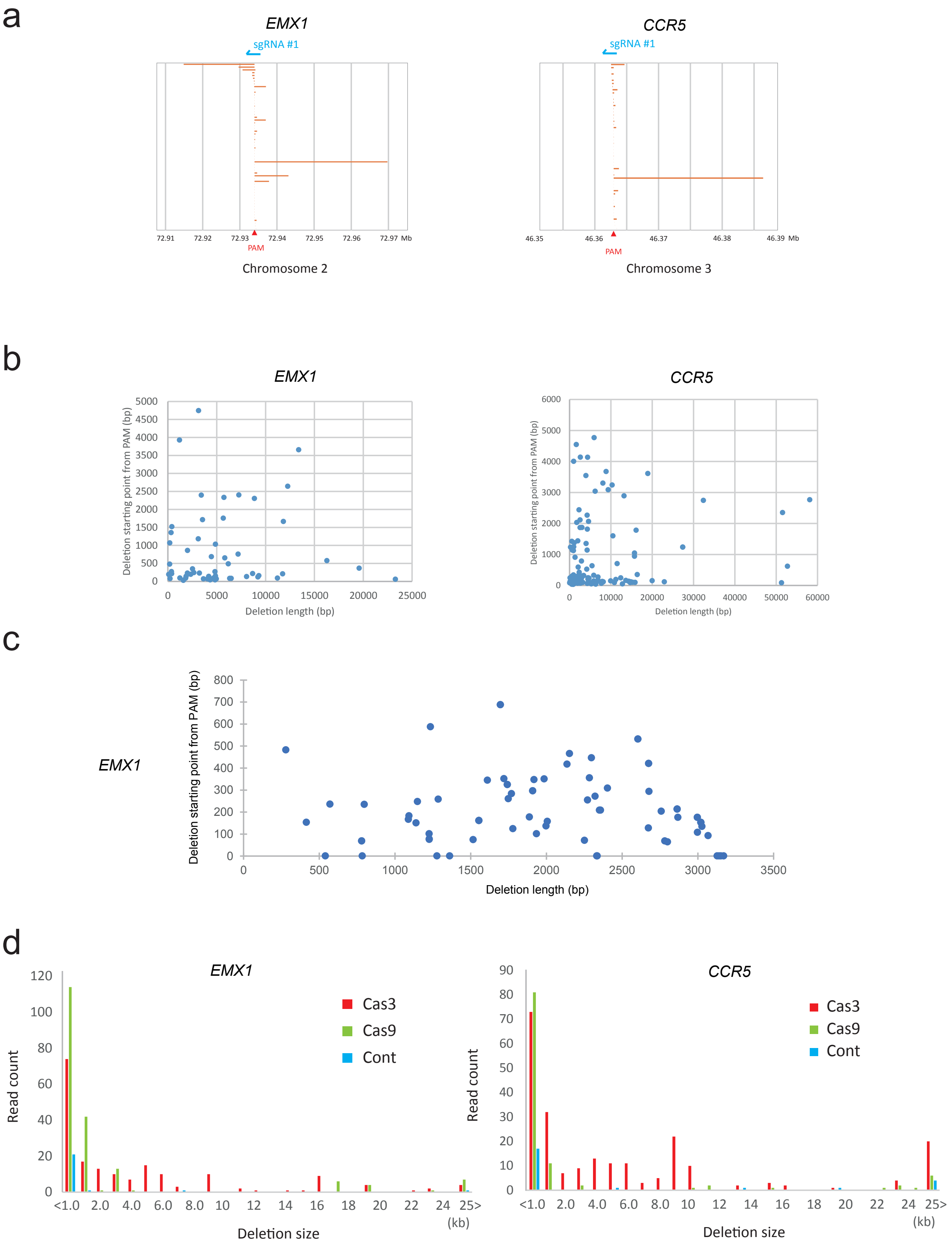

Supplementary Figure 10. Microarray-based capture sequencing of CRISPR-Cas targeting *EMX1* and *CCR5* loci. a, Cas9-mediated DNA deletion patterns via microarray-based capture sequencing at *EMX1* and *CCR5* loci in 293T cells. **b,** Cas3-mediated deletion lengths (X-axis) are plotted with deletion starting points (Y-axis) for *EMX1*- and *CCR5*-targeting in 293T cells. **c,** Cas3-mediated deletion patterns are plotted by Nanopore long-read sequencing of the PCR products at the *EMX1* target locus. **d,** Distribution patterns of the Cas3-mediated DNA deletion sizes counted by microarray-based capture sequencing for a 1-Mb region at the *EMX1* and *CCR5* loci. Discordant read pairs and split reads that were not correctly aligned with human genome assembly hg38 were counted.

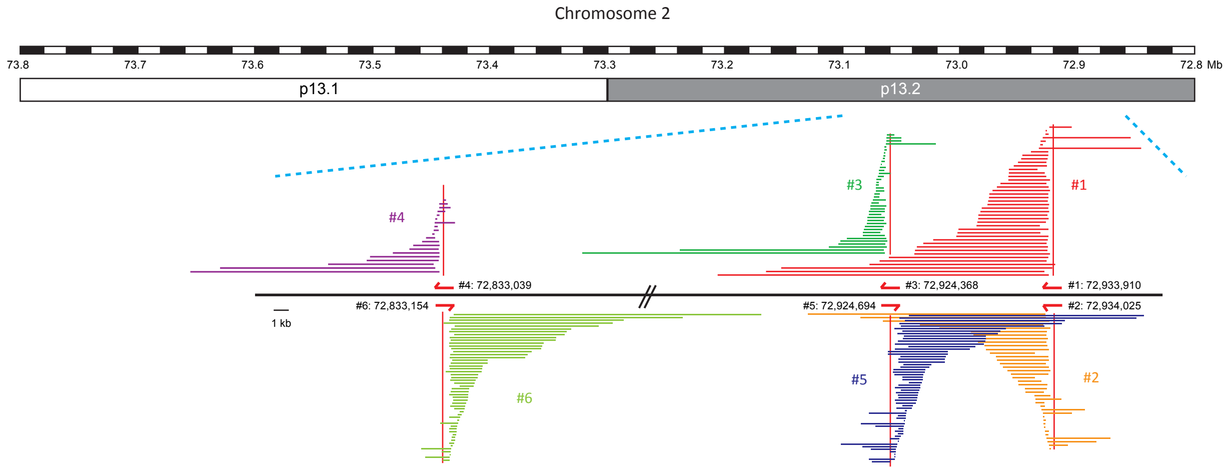

Supplementary Figure 11. Cas3-mediated DNA deletion patterns via microarray-based capture sequencing. Microarray-based capture sequencing with Cas3/crRNAs (#1–6) targeting a 1-Mb region at the *EMX1* loci. Cas3-mediated DNA deletion patterns are aligned with human genome assembly hg38.

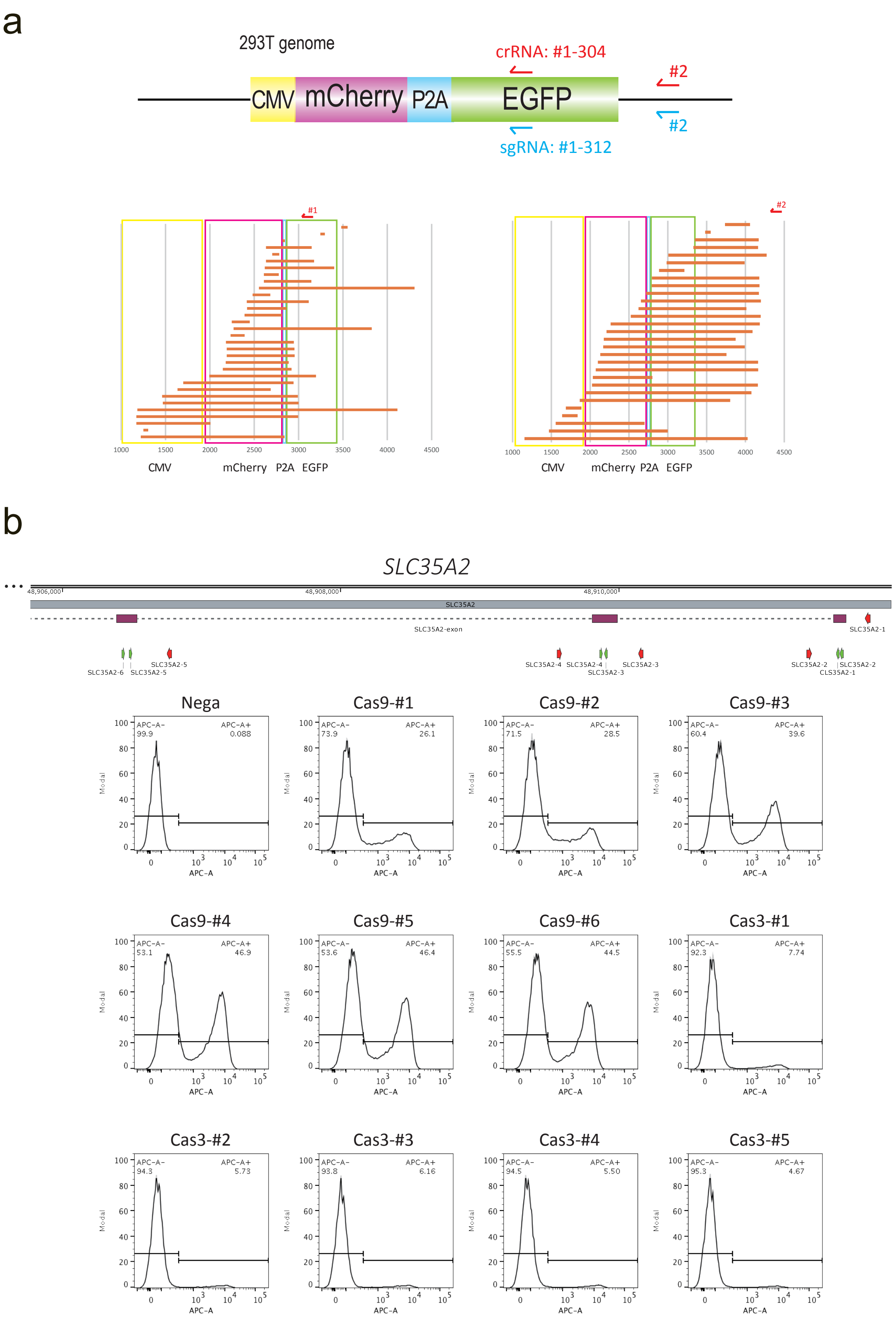

Supplementary Figure 12. Cas3-mediated knockout efficiency estimated by flow cytometry. a, Cas3-mediated deletions (orange bars) were confirmed by TOPO cloning and Sanger sequencing in 293T cells lacking GFP and mCherry double fluorescence. Many deletions disrupted the GFP and mCherry coding sequences, but few deletions disrupted the 5 ′ or 3 ′ UTR sequences, resulting in the loss of fluorescence. **b,** Knockout efficiencies of *SLC35A2* by Cascade/Cas3/crRNA (red arrows) and Cas9/gRNA (green arrows) were evaluated by flow cytometry with lectin II binding on 293T cells.

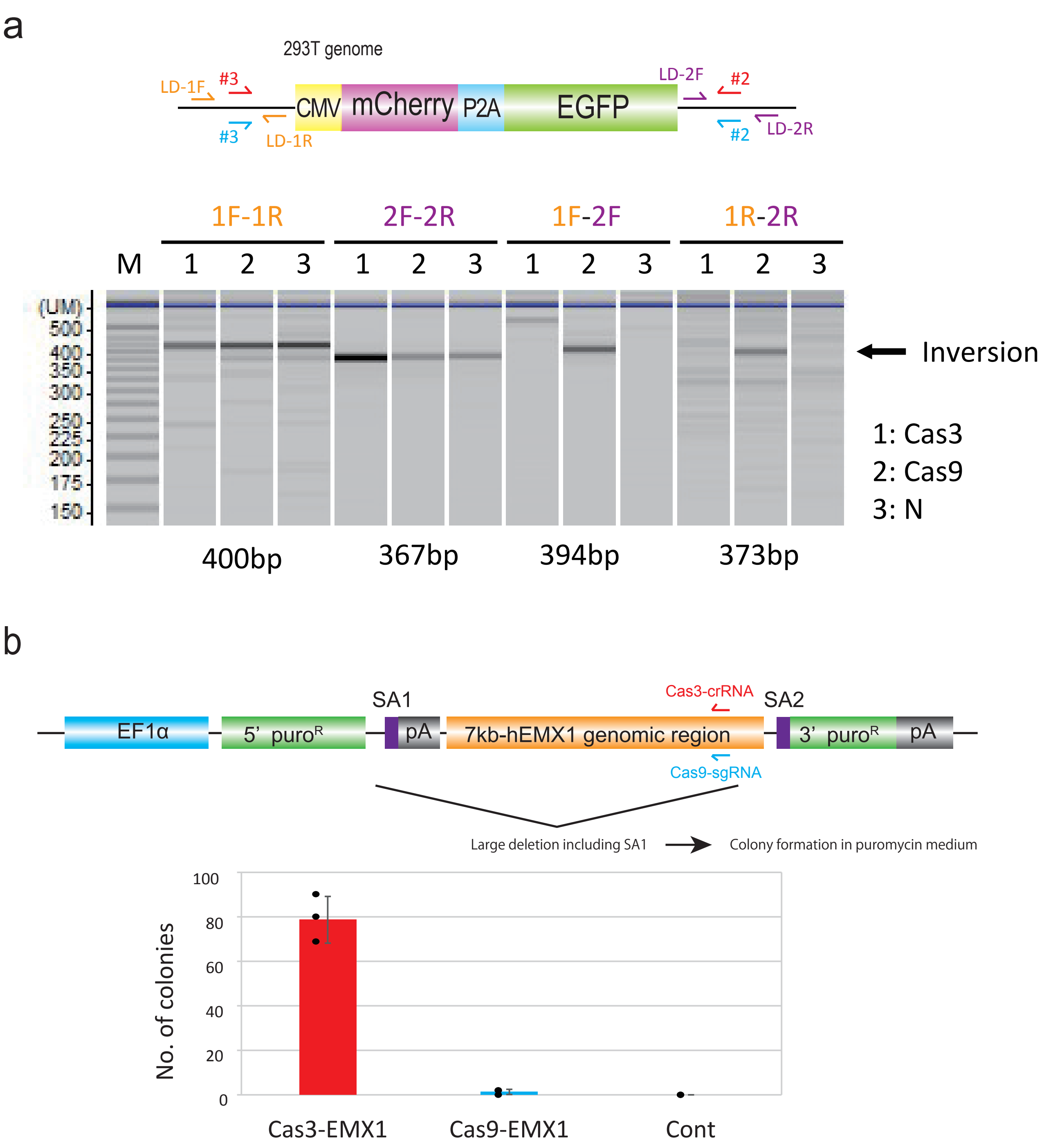

Supplementary Figure 13. Comparison of large deletions mediated by single crRNA-Cas3 and double gRNA-Cas9. a, PCR amplification using two boundary primer sets, 1F-2F or 1R-2R, indicates genomic inversions between the two cutting sites was induced by Cas9, but not by Cas3. **b,** A split puromycin resistance assay. CRISPR-induced large deletions in the 7.4 kb inserted sequences between the split puromycin gene recovered the resistance to puromycin selection, which increased the number of colonies. 293T cells (2 × 105) carrying Cascade genes were transfected with either Cas3 or Cas9 expression plasmids. The efficiency of the colony formation was approximately 0.04% with Cas3 expression plasmids. SA1: splicing acceptor derived from adenovirus, SA2: splicing acceptor derived from human *BCL2*. Data are presented as mean ± SD.

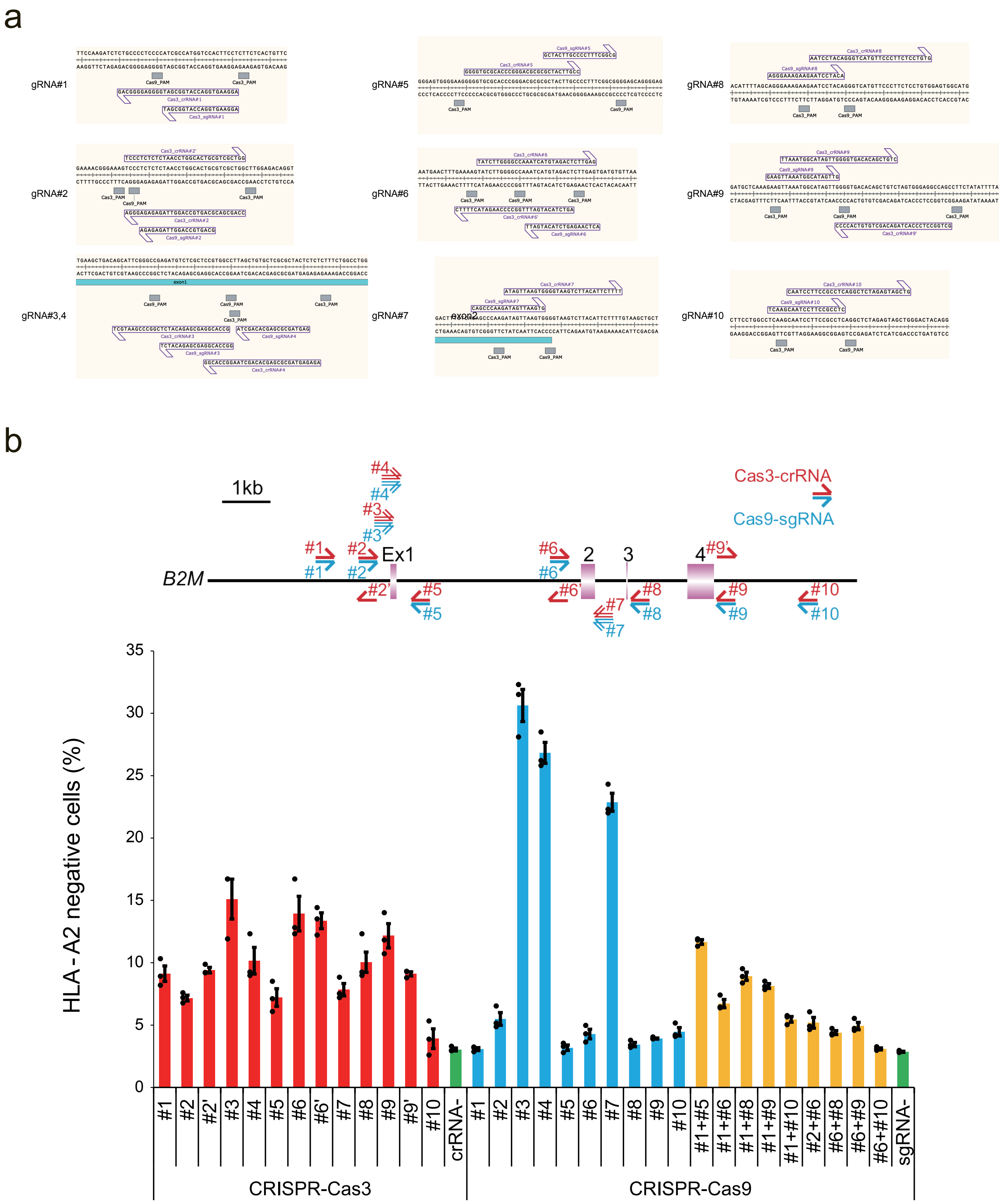

Supplementary Figure 14. Knockout efficiencies of cell surface B2M/HLA class I protein expression by CRISPR-Cas3 or -Cas9 in 293T cells. a, The sequence and position of each CRISPR-Cas3 crRNA and CRIPSR-Cas9 sgRNA for *B2M* KO in 293T cells. **b,** Schematic positions of the CRISPR-Cas3 crRNAs and CRIPSR-Cas9 sgRNAs targeting the *B2M* locus. The single line arrows indicate guide RNAs on untranslated regions, and the double line arrows indicate guide RNAs on *B2M*-translated regions. Comparison of *B2M* KO efficiencies between Cas3 (red), single Cas9 (blue), and dual Cas9 (yellow) evaluated by flow cytometry with anti-HLA-A2 antibody in 293T cells. Data are shown as mean ± s.e.m.

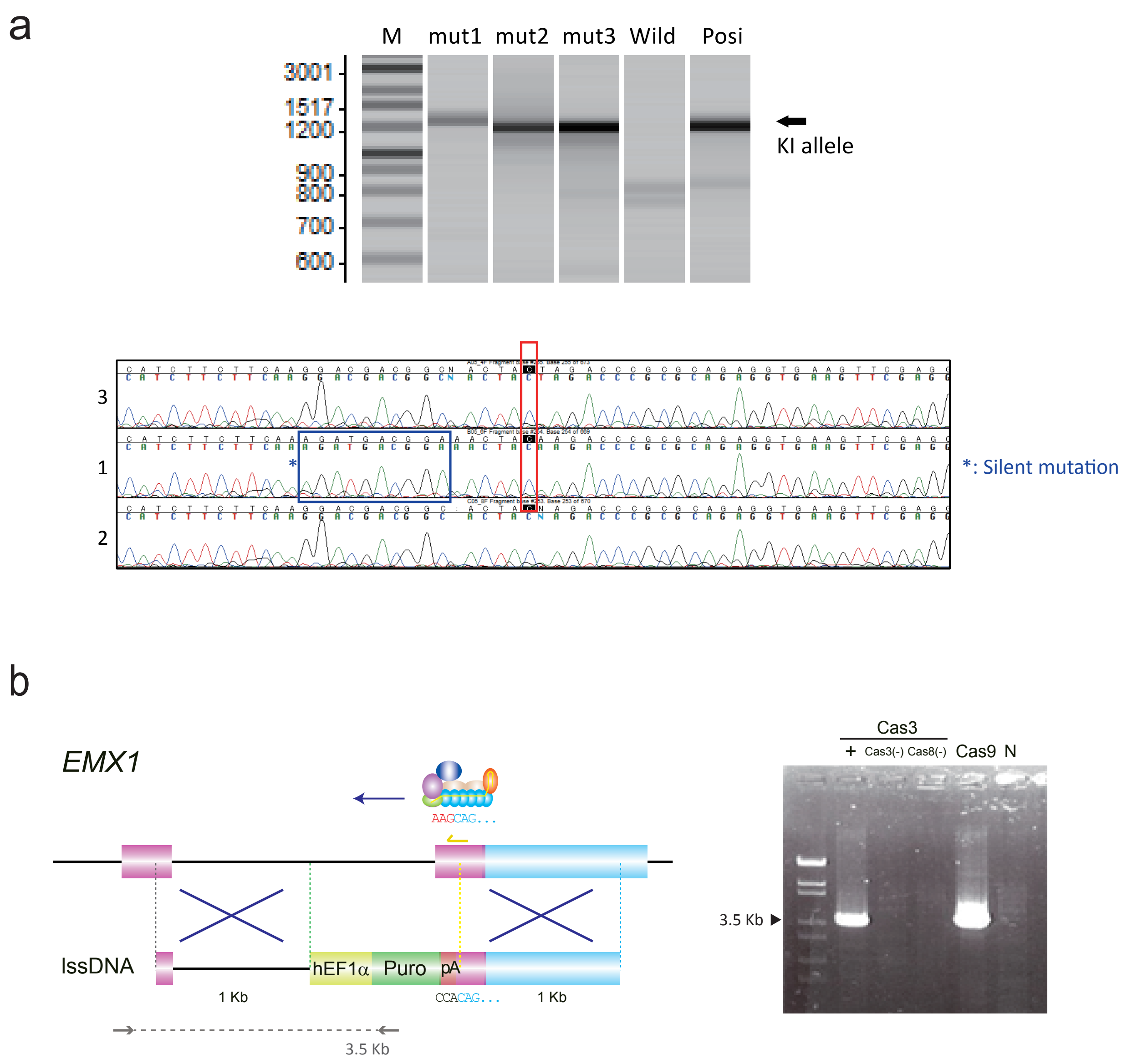

Supplementary Figure 15. CRISPR-Cas3 mediated functional gene knock-ins in 293T cells. a, HiDi PCR amplification and sequencing analysis confirmed a single nucleotide substitution by the CRISPR-Cas3 system with dsDNA in **Fig. 2g. b,** Schematic of Cas3-mediated functional gene knock-in of a puromycin gene (*Puro*) with lssDNA. A knock-in allele was amplified by PCR with primers; one designed inside the puromycin sequences, and the other designed outside the homology region.

a

PCR analysis at high PDD locus

Region	PDD score	detected regions		Size (bp)
chr1:29800001-29900000	3.53	chr1:29824973-29826193		1221
chr3:22100001-22200000	3.52	chr3:22156441-22162811		6371
chr3:28200001-28300000	3.78	chr3:28269935-28272593	chr3:28276152-28281177	7685
chr5:131400001-131500000	3.61	chr5:131447733-131466515	chr5:131470602-131481810	29992
chr6:143200001-143300000	3.54	chr6:143272976-143281354	chr6:143265274-143271955	15061
chr7:54100001-54200000	4.53	chr7:54177631-54185774	chr7:54192466-54195453	11132
chr10:84500001-84600000	4.22	chr10:84514726-84518845		4120
chr18:48700001-48800000	4.83	chr18:48788937-48796897		7961
chr18:74600001-74700000	3.53	chr18:74627712-74628851	chr18:74648217-74654711	7635
chrX:2700001-2800000	3.67	chrX:2703233-2712565		9333

b

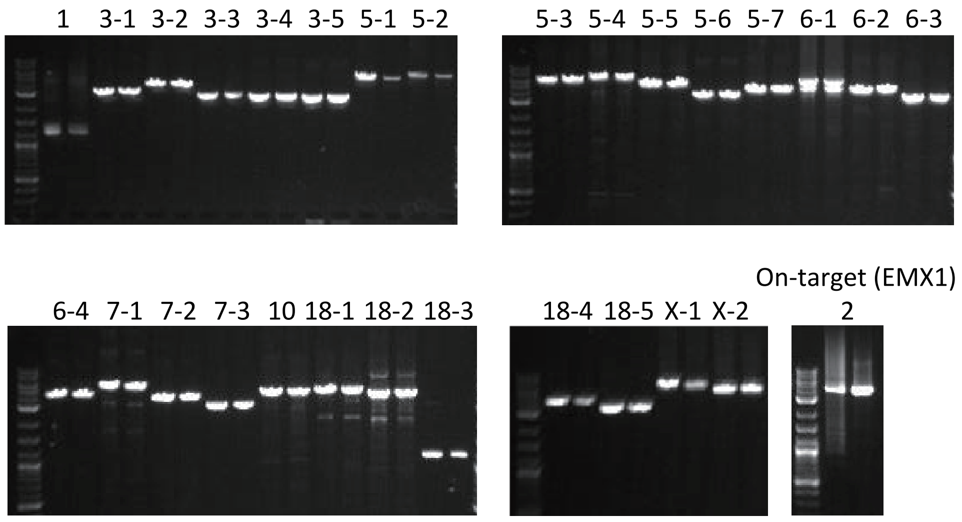

Supplementary Figure 16. PCR analysis for Cas3-mediated off-target effects at high POT loci. a, High potential DNA degradation (PDD)-score regions showing higher numbers of split and discordant reads were extracted. Primers designed to amplify the detected regions (see **Supplementary Table 10**) were used for PCR analysis. **b,** Cas-mediated off-target degradations were not detected by PCR. Left: CRISPR-Cas3 transfected; right: negative control. The first number of each primer set corresponds to the chromosome number shown in (**a**).

a

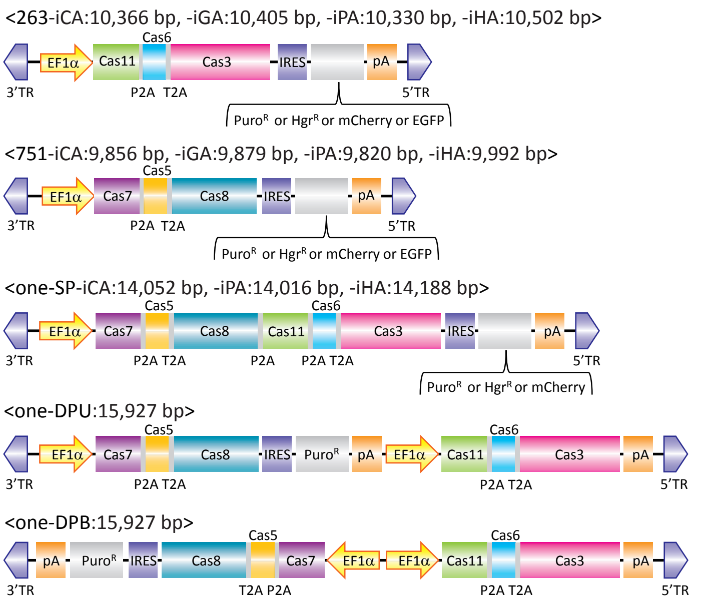

b

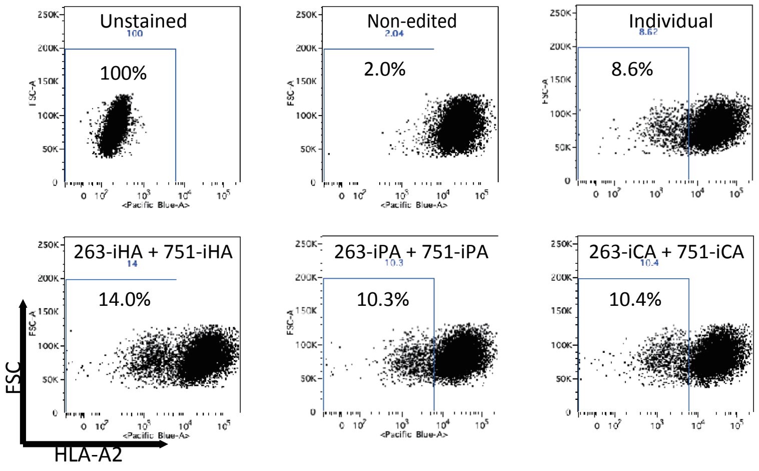

Supplementary Figure 17. Cas3-mediated *B2M* knockouts in human 293T cells. a, Polycistronic plasmids used for the expressions of Cas3, Cas5, Cas6, Cas7, Cas8, and Cas11 under an EF1α promoter with the selection markers puromycin (Puro), hygromycin B (Hgr), mCherry, or EGFP (iCA: IRES-mCherry-pA, iGA: IRES-EGFP-pA, iPA: IRES-Puro-pA, iHA: IRES-Hgr-pA). **b,** Knockout efficiencies of several CRISPR-Cas3 plasmids in (**a**) were evaluated by flow cytometry with anti-HLA-A2 antibody in 293T cells.

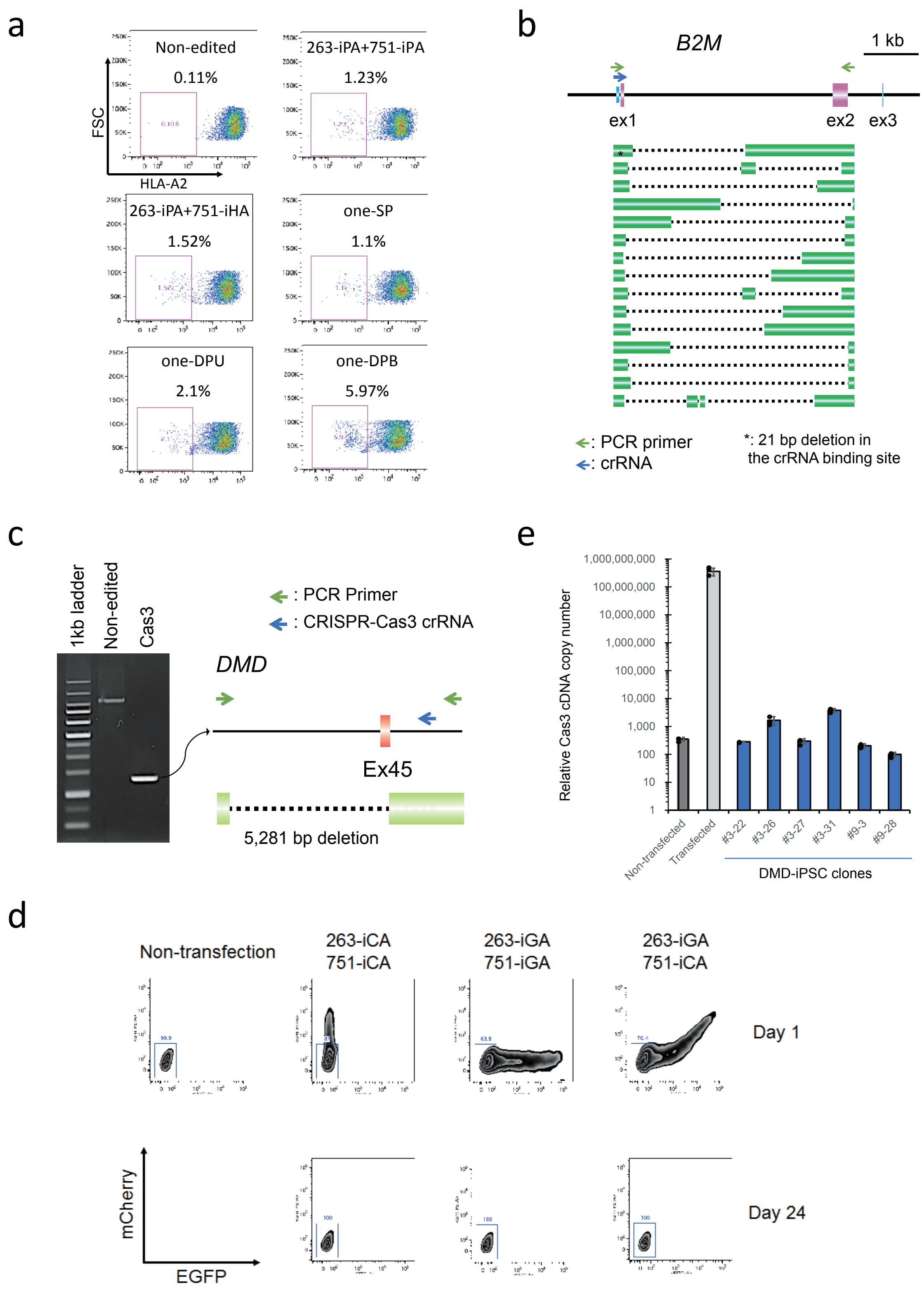

Supplementary Figure 18. Cas3-mediated *B2M* knockouts and *DMD* exon-skipping in iPSCs. **a,** Knockout efficiencies of cell surface B2M/HLA expression by polycistronic plasmids expressing CRISPR-Cas3 were evaluated by flow cytometry with anti-HLA-A2 antibody in iPSCs. **b,** Large deletion induced by Cas3 on the *B2M* gene target locus in the bulk iPS cells. Green bars indicate the sequenced regions, and black dotted lines indicate the deleted regions. **c,** A representative large deletion induced by the Cas3 system in DMD exon 45 in iPSCs. **d,** The expression from the plasmid vectors (iCA: IRES-mCherry-pA, iGA: IRES-EGFP-pA in **Supplementary Figure 17a**) was evaluated by flow cytometry after the transfection of iPSCs to show the transfection efficiencies of the Cas3/Cascade expression vectors. The expressions were diminished by day 24. **e,** Genomic integration of Cas3 cDNA was evaluated in corrected and isolated DMD-iPSC clones by using PCR and primers specific for Cas3 cDNA. Freshly transfected cells were used as a positive control. Data are shown as mean ± s.e.m.

Supplementary Table 1. Targets for SSA assay with luciferase-expressing plasmids.

Name	PAM (5' to 3'	Sequence (5' to 3')
Specificity of PAM		
SSA-hEMX1-AAG	AAG	CAGGCCAATGGGGAGGACATCGATGTCACCTC
SSA-hEMX1-AGG	AGG	CAGGCCAATGGGGAGGACATCGATGTCACCTC
SSA-hEMX1-GAG	GAG	CAGGCCAATGGGGAGGACATCGATGTCACCTC
SSA-hEMX1-TAC	TAC	CAGGCCAATGGGGAGGACATCGATGTCACCTC
SSA-hEMX1-ATG	ATG	CAGGCCAATGGGGAGGACATCGATGTCACCTC
SSA-hEMX1-TAG	TAG	CAGGCCAATGGGGAGGACATCGATGTCACCTC
SSA-hEMX1-AAC	AAC	CAGGCCAATGGGGAGGACATCGATGTCACCTC
SSA-hEMX1-AAT	AAT	CAGGCCAATGGGGAGGACATCGATGTCACCTC
SSA-hEMX1-GAC	GAC	CAGGCCAATGGGGAGGACATCGATGTCACCTC
SSA-hEMX1-CAG	CAG	CAGGCCAATGGGGAGGACATCGATGTCACCTC
SSA-hEMX1-CCA	CCA	CAGGCCAATGGGGAGGACATCGATGTCACCTC
Specificity of the seed sequence		
SSA-hEMX1-Mu1	AAG	GAGGCCAATGGGGAGGACATCGATGTCACCTC
SSA-hEMX1-Mu2	AAG	CTGGCCAATGGGGAGGACATCGATGTCACCTC
SSA-hEMX1-Mu3	AAG	CACGCCAATGGGGAGGACATCGATGTCACCTC
SSA-hEMX1-Mu4	AAG	CAGCCCAATGGGGAGGACATCGATGTCACCTC
SSA-hEMX1-Mu5	AAG	CAGGGCAATGGGGAGGACATCGATGTCACCTC
SSA-hEMX1-Mu6	AAG	CAGGCGAATGGGGAGGACATCGATGTCACCTC
SSA-hEMX1-Mu7	AAG	CAGGCCTATGGGGAGGACATCGATGTCACCTC
SSA-hEMX1-Mu8	AAG	CAGGCCATTGGGGAGGACATCGATGTCACCTC
SSA-hEMX1-Mu9	AAG	CAGGCCAAAGGGGAGGACATCGATGTCACCTC
SSA-hEMX1-Mu10	AAG	CAGGCCAATCGGGAGGACATCGATGTCACCTC
SSA-hEMX1-Mu11	AAG	CAGGCCAATGCGGAGGACATCGATGTCACCTC
SSA-hEMX1-Mu12	AAG	CAGGCCAATGGCGAGGACATCGATGTCACCTC
SSA-hEMX1-Mu13	AAG	CAGGCCAATGGGCAGGACATCGATGTCACCTC
SSA-hEMX1-Mu14	AAG	CAGGCCAATGGGGTGGACATCGATGTCACCTC
SSA-hEMX1-Mu15	AAG	CAGGCCAATGGGGACGACATCGATGTCACCTC
SSA-hEMX1-Mu16	AAG	CAGGCCAATGGGGAGCACATCGATGTCACCTC
SSA-hEMX1-Mu17	AAG	CAGGCCAATGGGGAGGTCATCGATGTCACCTC
SSA-hEMX1-Mu18	AAG	CAGGCCAATGGGGAGGAGATCGATGTCACCTC
SSA-hEMX1-Mu19	AAG	CAGGCCAATGGGGAGGACTTCGATGTCACCTC
SSA-hEMX1-Mu20	AAG	CAGGCCAATGGGGAGGACAACGATGTCACCTC
SSA-hEMX1-Mu21	AAG	CAGGCCAATGGGGAGGACATGGATGTCACCTC
SSA-hEMX1-Mu22	AAG	CAGGCCAATGGGGAGGACATCCATGTCACCTC
SSA-hEMX1-Mu23	AAG	CAGGCCAATGGGGAGGACATCGTTGTCACCTC
SSA-hEMX1-Mu24	AAG	CAGGCCAATGGGGAGGACATCGAAGTCACCTC
SSA-hEMX1-Mu25	AAG	CAGGCCAATGGGGAGGACATCGATCTCACCTC
SSA-hEMX1-Mu26	AAG	CAGGCCAATGGGGAGGACATCGATGACACCTC
SSA-hEMX1-Mu27	AAG	CAGGCCAATGGGGAGGACATCGATGTGACCTC
SSA-hEMX1-Mu28	AAG	CAGGCCAATGGGGAGGACATCGATGTCTCCTC
SSA-hEMX1-Mu29	AAG	CAGGCCAATGGGGAGGACATCGATGTCAGCTC
SSA-hEMX1-Mu30	AAG	CAGGCCAATGGGGAGGACATCGATGTCACGTC
SSA-hEMX1-Mu31	AAG	CAGGCCAATGGGGAGGACATCGATGTCACCAC
SSA-hEMX1-Mu32	AAG	CAGGCCAATGGGGAGGACATCGATGTCACCTG
Subtypes of CRISPR-Cas		
SSA-I-E-GFP	AAG	CACTGCACGCCGTAGGTGAAGGTGGTCACGAG
SSA-I-F-GFP	CCC	ACCCTCGTGACCACCTTCACCTACGGCGTGCA
SSA-I-G-GFP	CTG	CACGCCGTAGGTGAAGGTGGTCACGAGGGTGGCCAG
SSA-II-A-GFP	CCG	TAGGTGAAGGTGGTCACGAG

SSA; single-strand annealing. PAM; protospacer adjacent motif.

Supplementary Table 2. NGS of PCR amplicons at the *EMX1* and *CCR5* locus.

Target locus		PAM	position (hg19)	Total reads	Average of coverage depth	Coverage ratio for 1kb region upstream PAM (%)	Coverage ratio for 3kb region upstream PAM (%)	Minimum coverage position from PAM	Estimated editing efficiency (%)
EMX1 (Chr2:73158033-73161550)	I-E (Cas3)	AAG	73161039	22727159	6460.2	61.6	71.2	337	88.9
		ATG	73161049	30815123	8759.3	63.1	76.1	2396	71.2
		TTT	73161182	21250745	6040.6	102.2	100.6	3143	3.8
	Cas9	NGG	73161086	30088191	8552.6	97.5	101.1	4	50.0
	Cont.	-	73161039	36986487	10513.5	101.8	100.0	2518	-
CCR5 (Chr3:46405333-46414741)	I-E (Cas3)	AAG	46414411	232754572	24737.4	87.9	78.8	5554*	55.1
		ATG	46414394	187886639	19968.8	102.1	82.5	3471	55.9
		TTT	46414520	224798494	23891.9	106.4	104.0	5663*	0.0
	Cas9	NGG	46414443	225185984	23933.0	100.1	102.4	5586*	43.2
	Cont.	-	46414411	199277518	21179.5	102.3	101.8	5554*	-

*Minimum coverage position at CCR5 region is detected at the same repetitive region.

Supplementary Table 3. Editing efficiency by NGS of PCR amplicons at several target genes.

Target locus	Chr	PAM locus of Cas3 (hg38)	PAM locus of Cas9 (hg38)	Cas3 KO efficiency (%)	Cas9 KO efficiency (%)
EMX1	*2*	72931705	72931701	60.3	78.2
CCR5	*3*	46364243	46364232	46.3	53.0
TET2	*4*	105234108	105234101	19.0	49.7
DMD	*X*	31967651	31967638	29.4	28.2
B2M	*15*	44711563	44711559	24.4	76.2
AAVS1	*19*	55115800	55115805	7.3	15.3
VEGFA	*6*	43774458	43774434	14.7	36.5
ERCC4	*16*	13937859	13937890	11.5	38.7
Average				26.6	47.0

Supplementary Table 4. Microarray-based capture sequencing around the target site.

Target	target locus beside PAM(hg38)	Average of coverage depth	Average size of the deletion (bp)	Maximum size of the deletion from split read (bp)	KO efficiency (%)
EMX1-1	72933913	2220.1	6450.8	23293	16.1
EMX1-2	72934025	1673.4	2963.4	13856	4.4
EMX1-3	72924368	1716.3	3464.4	77682	5.6
EMX1-4	72833039	1315.2	6269.4	37488	38.2
EMX1-Cas9	72933956	2785.0	668.2	35785	23.4*
CCR5-1	46372923	2420.0	7088.1	51280	4.6
CCR5-2	46373082	1478.9	7613.1	34689	15.5
CCR5-3	46365788	1351.3	4713.8	14965	16.6
CCR5-4	46270992	1199.4	4001.6	28990	8.0
CCR5-Cas9	46372951	2339.7	542.5	23478	23.7*

* Calculated via CRISPResso2

Supplementary Table 5. List of potential off-target sites by perfect match detection with the on-target *EMX1* site.

<table>
<tr><th>chromosome</th><th>position</th><th>strand</th><th># of match</th><th>off-target sequence</th><th>alignment</th></tr>
<tr><td>chr2 (On-target)</td><td>72933910</td><td>+</td><td>35</td><td>AAGCAGGCCAATGGGGAGGACATCGATGTCACCTC</td><td>CAGGC*AATGG*GAGGA*ATCGA*GTCAC*TC
||||||||||||||||||||||||||||||||
AAGCAGGCCAATGGGGAGGACATCGATGTCACCTC</td></tr>
<tr><td>chr12</td><td>41368146</td><td>-</td><td>21</td><td>TTCCTCACCATTTGCCTGCCT</td><td>*TCCTC*CCATT*GCCTG
||||||||||||||||||
TTCCTCACCATTTGCCTGCCT</td></tr>
<tr><td>chr4</td><td>90689737</td><td>+</td><td>18</td><td>AACCAGGCCAATGGAGAG</td><td>CAGGC*AATGG*GAG
|||||||||||||||
AACCAGGCCAATGGAGAG</td></tr>
<tr><td>chr4</td><td>1.54E+08</td><td>+</td><td>18</td><td>ATGCAGGCAAATGGAGAG</td><td>CAGGC*AATGG*GAG
|||||||||||||||
ATGCAGGCAAATGGAGAG</td></tr>
<tr><td>chr6</td><td>1810751</td><td>+</td><td>18</td><td>AAGCAGGCCAATGGCGAG</td><td>CAGGC*AATGG*GAG
|||||||||||||||
AAGCAGGCCAATGGCGAG</td></tr>
<tr><td>chr11</td><td>1.14E+08</td><td>-</td><td>18</td><td>CTCTCCATTTGCCTGCTC</td><td>CTC*CCATT*GCCTG
|||||||||||||||
CTCTCCATTTGCCTGCTC</td></tr>
<tr><td>chr11</td><td>1.2E+08</td><td>-</td><td>18</td><td>CTCTCCATTTGCCTGCTC</td><td>CTC*CCATT*GCCTG
|||||||||||||||
CTCTCCATTTGCCTGCTC</td></tr>
<tr><td>chr12</td><td>1.16E+08</td><td>+</td><td>18</td><td>GAGCAGGCTAATGGGGAG</td><td>CAGGC*AATGG*GAG
|||||||||||||||
GAGCAGGCTAATGGGGAG</td></tr>
<tr><td>chr12</td><td>1.2E+08</td><td>-</td><td>18</td><td>CTCCCCATTTGCCTGGTT</td><td>CTC*CCATT*GCCTG
|||||||||||||||
CTCCCCATTTGCCTGGTT</td></tr>
<tr><td>chr15</td><td>74789038</td><td>-</td><td>18</td><td>CTCCCCATTAGCCTGCTC</td><td>CTC*CCATT*GCCTG
|||||||||||||||
CTCCCCATTAGCCTGCTC</td></tr>
<tr><td>chr17</td><td>21457647</td><td>-</td><td>18</td><td>CTCCCCATTCGCCTGCCT</td><td>CTC*CCATT*GCCTG
|||||||||||||||
CTCCCCATTCGCCTGCCT</td></tr>
<tr><td>chr18</td><td>41817452</td><td>+</td><td>18</td><td>AACCAGGCAAATGGGGAG</td><td>CAGGC*AATGG*GAG
|||||||||||||||
AACCAGGCAAATGGGGAG</td></tr>
<tr><td>chrX</td><td>83888730</td><td>+</td><td>18</td><td>AGGCAGGCCAATGGGGAG</td><td>CAGGC*AATGG*GAG
|||||||||||||||
AGGCAGGCCAATGGGGAG</td></tr>
<tr><td>chr1</td><td>36224887</td><td>-</td><td>17</td><td>TCCCCATTGGCCTGGTT</td><td>TC*CCATT*GCCTG
||||||||||||||
TCCCCATTGGCCTGGTT</td></tr>
<tr><td>chr1</td><td>1.83E+08</td><td>+</td><td>17</td><td>AACCAGGCCAATGGCGA</td><td>CAGGC*AATGG*GA
||||||||||||||
AACCAGGCCAATGGCGA</td></tr>
<tr><td>chr1</td><td>2.21E+08</td><td>-</td><td>17</td><td>TCACCATTTGCCTGCTT</td><td>TC*CCATT*GCCTG
||||||||||||||
TCACCATTTGCCTGCTT</td></tr>
<tr><td>chr2</td><td>75697626</td><td>+</td><td>17</td><td>TAGCAGGCTAATGGTGA</td><td>CAGGC*AATGG*GA
||||||||||||||
TAGCAGGCTAATGGTGA</td></tr>
<tr><td>chr6</td><td>11277872</td><td>-</td><td>17</td><td>TCTCCATTTGCCTGCCT</td><td>TC*CCATT*GCCTG
||||||||||||||
TCTCCATTTGCCTGCCT</td></tr>
<tr><td>chr6</td><td>48299032</td><td>+</td><td>17</td><td>AACCAGGCAAATGGGGA</td><td>CAGGC*AATGG*GA
||||||||||||||
AACCAGGCAAATGGGGA</td></tr>
<tr><td>chr6</td><td>92480065</td><td>-</td><td>17</td><td>TCTCCATTAGCCTGCTT</td><td>TC*CCATT*GCCTG
||||||||||||||
TCTCCATTAGCCTGCTT</td></tr>
<tr><td>chr11</td><td>30166045</td><td>+</td><td>17</td><td>AGGCAGGCCAATGGGGA</td><td>CAGGC*AATGG*GA
||||||||||||||
AGGCAGGCCAATGGGGA</td></tr>
<tr><td>chr11</td><td>40647755</td><td>-</td><td>17</td><td>TCACCATTTGCCTGCCT</td><td>TC*CCATT*GCCTG
||||||||||||||
TCACCATTTGCCTGCCT</td></tr>
<tr><td>chr11</td><td>73215873</td><td>-</td><td>17</td><td>TCACCATTAGCCTGCCT</td><td>TC*CCATT*GCCTG
||||||||||||||
TCACCATTAGCCTGCCT</td></tr>
<tr><td>chr14</td><td>70117996</td><td>-</td><td>17</td><td>TCCCCATTGGCCTGCAT</td><td>TC*CCATT*GCCTG
||||||||||||||
TCCCCATTGGCCTGCAT</td></tr>
<tr><td>chr18</td><td>27837509</td><td>+</td><td>17</td><td>AGGCAGGCGAATGGAGA</td><td>CAGGC*AATGG*GA
||||||||||||||
AGGCAGGCGAATGGAGA</td></tr>
<tr><td>chr18</td><td>30217418</td><td>-</td><td>17</td><td>TCCCCATTAGCCTGGTT</td><td>TC*CCATT*GCCTG
||||||||||||||
TCCCCATTAGCCTGGTT</td></tr>
<tr><td>chr22</td><td>19386186</td><td>-</td><td>17</td><td>TCCCCATTGGCCTGCTT</td><td>TC*CCATT*GCCTG
||||||||||||||
TCCCCATTGGCCTGCTT</td></tr>
<tr><td>chrX</td><td>85683796</td><td>-</td><td>17</td><td>TCTCCATTGGCCTGGTT</td><td>TC*CCATT*GCCTG
||||||||||||||
TCTCCATTGGCCTGGTT</td></tr>
<tr><td>chrX</td><td>1.04E+08</td><td>+</td><td>17</td><td>TAGCAGGCCAATGGGGA</td><td>CAGGC*AATGG*GA
||||||||||||||
TAGCAGGCCAATGGGGA</td></tr>
</table>

Supplementary Table 6. List of potential off-target sites by perfect match detection with the on-target *CCR5* site.

<table>
<tr><th>chromosome</th><th>position</th><th>strand</th><th># of match</th><th>off-target sequence</th><th>alignment</th></tr>
<tr><td>chr3 (On-target)</td><td>46372920</td><td>+</td><td>35</td><td>AAGTCCAATCTATGACATCAATTATTATACATCGG</td><td>TCCAA*CTATG*CATCA*TTATT*TACAT*GG
||||||||||||||||||||||||||||||||
AAGTCCAATCTATGACATCAATTATTATACATCGG</td></tr>
<tr><td>chr2</td><td>74151726</td><td>-</td><td>18</td><td>ATGGCATAGTTTGGACAT</td><td>ATG*CATAG*TTGGA
|||||||||||||||
ATGGCATAGTTTGGACAT</td></tr>
<tr><td>chr2</td><td>2.32E+08</td><td>-</td><td>18</td><td>ATGGCATAGATTGGAGTT</td><td>ATG*CATAG*TTGGA
|||||||||||||||
ATGGCATAGATTGGAGTT</td></tr>
<tr><td>chr3</td><td>59420177</td><td>+</td><td>18</td><td>AACTCCAAACTATGTCAT</td><td>TCCAA*CTATG*CAT
|||||||||||||||
AACTCCAAACTATGTCAT</td></tr>
<tr><td>chr3</td><td>1.03E+08</td><td>+</td><td>18</td><td>TAGTCCAACCTATGGCAT</td><td>TCCAA*CTATG*CAT
|||||||||||||||
TAGTCCAACCTATGGCAT</td></tr>
<tr><td>chr9</td><td>21485987</td><td>+</td><td>18</td><td>AACTCCAAACTATGACAT</td><td>TCCAA*CTATG*CAT
|||||||||||||||
AACTCCAAACTATGACAT</td></tr>
<tr><td>chr11</td><td>1.22E+08</td><td>-</td><td>18</td><td>ATGACATAGCTTGGACAT</td><td>ATG*CATAG*TTGGA
|||||||||||||||
ATGACATAGCTTGGACAT</td></tr>
<tr><td>chrX</td><td>39589780</td><td>+</td><td>18</td><td>TAGTCCAAGCTATGACAT</td><td>TCCAA*CTATG*CAT
|||||||||||||||
TAGTCCAAGCTATGACAT</td></tr>
<tr><td>chrX</td><td>1.48E+08</td><td>-</td><td>18</td><td>ATGACATAGTTTGGACAT</td><td>ATG*CATAG*TTGGA
|||||||||||||||
ATGACATAGTTTGGACAT</td></tr>
<tr><td>chrX</td><td>1.53E+08</td><td>+</td><td>18</td><td>ATGTCCAAACTATGTCAT</td><td>TCCAA*CTATG*CAT
|||||||||||||||
ATGTCCAAACTATGTCAT</td></tr>
<tr><td>chr1</td><td>89936327</td><td>-</td><td>17</td><td>TGACATAGTTTGGAGTT</td><td>TG*CATAG*TTGGA
||||||||||||||
TGACATAGTTTGGAGTT</td></tr>
<tr><td>chr2</td><td>2452198</td><td>+</td><td>17</td><td>ATGTCCAAACTATGGCA</td><td>TCCAA*CTATG*CA
||||||||||||||
ATGTCCAAACTATGGCA</td></tr>
<tr><td>chr3</td><td>13584260</td><td>-</td><td>17</td><td>TGCCATAGCTTGGACTC</td><td>TG*CATAG*TTGGA
||||||||||||||
TGCCATAGCTTGGACTC</td></tr>
<tr><td>chr3</td><td>22065347</td><td>+</td><td>17</td><td>TAGTCCAATCTATGCCA</td><td>TCCAA*CTATG*CA
||||||||||||||
TAGTCCAATCTATGCCA</td></tr>
<tr><td>chr5</td><td>21833772</td><td>+</td><td>17</td><td>AGGTCCAAGCTATGTCA</td><td>TCCAA*CTATG*CA
||||||||||||||
AGGTCCAAGCTATGTCA</td></tr>
<tr><td>chr6</td><td>94750894</td><td>-</td><td>17</td><td>TGACATAGTTTGGACAT</td><td>TG*CATAG*TTGGA
||||||||||||||
TGACATAGTTTGGACAT</td></tr>
<tr><td>chr6</td><td>1.22E+08</td><td>-</td><td>17</td><td>TGGCATAGATTGGAGTT</td><td>TG*CATAG*TTGGA
||||||||||||||
TGGCATAGATTGGAGTT</td></tr>
<tr><td>chr6</td><td>1.68E+08</td><td>+</td><td>17</td><td>AACTCCAACCTATGCCA</td><td>TCCAA*CTATG*CA
||||||||||||||
AACTCCAACCTATGCCA</td></tr>
<tr><td>chr7</td><td>10390779</td><td>-</td><td>17</td><td>TGCCATAGCTTGGAGTT</td><td>TG*CATAG*TTGGA
||||||||||||||
TGCCATAGCTTGGAGTT</td></tr>
<tr><td>chr8</td><td>80930276</td><td>+</td><td>17</td><td>ATGTCCAAACTATGCCA</td><td>TCCAA*CTATG*CA
||||||||||||||
ATGTCCAAACTATGCCA</td></tr>
<tr><td>chr9</td><td>1.35E+08</td><td>-</td><td>17</td><td>TGGCATAGATTGGACAT</td><td>TG*CATAG*TTGGA
||||||||||||||
TGGCATAGATTGGACAT</td></tr>
<tr><td>chr10</td><td>25217660</td><td>+</td><td>17</td><td>GAGTCCAACCTATGGCA</td><td>TCCAA*CTATG*CA
||||||||||||||
GAGTCCAACCTATGGCA</td></tr>
<tr><td>chr10</td><td>1.16E+08</td><td>-</td><td>17</td><td>TGACATAGTTTGGACCT</td><td>TG*CATAG*TTGGA
||||||||||||||
TGACATAGTTTGGACCT</td></tr>
<tr><td>chr11</td><td>45705713</td><td>+</td><td>17</td><td>GAGTCCAAGCTATGTCA</td><td>TCCAA*CTATG*CA
||||||||||||||
GAGTCCAAGCTATGTCA</td></tr>
<tr><td>chr12</td><td>1.07E+08</td><td>-</td><td>17</td><td>TGCCATAGTTTGGAGTT</td><td>TG*CATAG*TTGGA
||||||||||||||
TGCCATAGTTTGGAGTT</td></tr>
<tr><td>chr16</td><td>52919021</td><td>+</td><td>17</td><td>AAGTCCAAGCTATGTCA</td><td>TCCAA*CTATG*CA
||||||||||||||
AAGTCCAAGCTATGTCA</td></tr>
<tr><td>chr17</td><td>26857085</td><td>-</td><td>17</td><td>TGACATAGATTGGACTC</td><td>TG*CATAG*TTGGA
||||||||||||||
TGACATAGATTGGACTC</td></tr>
<tr><td>chr18</td><td>66220529</td><td>-</td><td>17</td><td>TGCCATAGTTTGGAGTT</td><td>TG*CATAG*TTGGA
||||||||||||||
TGCCATAGTTTGGAGTT</td></tr>
<tr><td>chr20</td><td>2020422</td><td>+</td><td>17</td><td>AACTCCAAACTATGTCA</td><td>TCCAA*CTATG*CA
||||||||||||||
AACTCCAAACTATGTCA</td></tr>
<tr><td>chr20</td><td>57831923</td><td>+</td><td>17</td><td>TAGTCCAAACTATGTCA</td><td>TCCAA*CTATG*CA
||||||||||||||
TAGTCCAAACTATGTCA</td></tr>
<tr><td>chrX</td><td>1.26E+08</td><td>+</td><td>17</td><td>AACTCCAAGCTATGTCA</td><td>TCCAA*CTATG*CA
||||||||||||||
AACTCCAAGCTATGTCA</td></tr>
</table>

Supplementary Table 7. List of potential off-target sites by mismatch detection with the on-target *EMX1* site.

chromosome	position	strand	# of mismatch	off-target sequence	alignment
chr2 (On-target)	72933910	+	0	AAGCAGGCCAATGGGGAGGACATCGATGTCACCTC	CAGGC*AATGG*GAGGA*ATCGA*GTCAC*TC<br>|||||||||||||||||||||||||||||||| AAGCAGGCCAATGGGGAGGACATCGATGTCACCTC
chr2	28684889	-	5	GAAATGACCTCGATCTCCTCTGACTTCTCCTGCAT	GA*GTGAC*TCGAT*TCCTC*CCATT*GCCTG<br>||| ||||||||||||||||| ||| |||| GAAATGACCTCGATCTCCTCTGACTTCTCCTGCAT
chr10	1.18E+08	+	5	AAGCAGGCGAGTCCGGAGGAAATAGACGTGACCTC	CAGGC*AATGG*GAGGA*ATCGA*GTCAC*TC<br>||||||| | ||||||||| ||||| ||||| AAGCAGGCGAGTCCGGAGGAAATAGACGTGACCTC
chr2	2.28E+08	-	6	AAGGTGTCCTTGATGTCCTTGCCATTGTCCTTGTT	GA*GTGAC*TCGAT*TCCTC*CCATT*GCCTG<br>||||| ||| |||||||| ||||||| ||| AAGGTGTCCTTGATGTCCTTGCCATTGTCCTTGTT
chr11	65018398	+	6	AAGAAGTTCAATGGGGAGGACATCGACACCATCTC	CAGGC*AATGG*GAGGA*ATCGA*GTCAC*TC<br>|| ||||||||||||||||||| || ||| AAGAAGTTCAATGGGGAGGACATCGACACCATCTC
chr12	39315192	-	6	GAAATGAAATTAATTTCCTCTCCCTTCCCCTGCCT	GA*GTGAC*TCGAT*TCCTC*CCATT*GCCTG<br>||| ||| || ||||||||||| ||| |||| GAAATGAAATTAATTTCCTCTCCCTTCCCCTGCCT
chr18	24467416	+	6	AACCAGGAAAGTGCAGAGGACAGAGGGGTCACTTC	CAGGC*AATGG*GAGGA*ATCGA*GTCAC*TC<br>|||| || || |||||||| | ||||||||| AACCAGGAAAGTGCAGAGGACAGAGGGGTCACTTC
chr18	76982682	-	6	GCTATGAAGTCCCTGTCCTCTCCATCTGCCTGCCT	GA*GTGAC*TCGAT*TCCTC*CCATT*GCCTG<br>| | ||| ||| |||||||||||| |||||| GCTATGAAGTCCCTGTCCTCTCCATCTGCCTGCCT
chr1	10321585	+	7	AAGCAGCTGAATGGAGAGAAACTGGAAAACACCTC	CAGGC*AATGG*GAGGA*ATCGA*GTCAC*TC<br>||| |||||||||| || | ||| |||||| AAGCAGCTGAATGGAGAGAAACTGGAAAACACCTC
chr1	76379920	+	7	AGGAAGAGGAAGGGGGAGGATATGGAGGTTACCTG	CAGGC*AATGG*GAGGA*ATCGA*GTCAC*TC<br>|| ||| ||||||||||| ||||| |||| AGGAAGAGGAAGGGGGAGGATATGGAGGTTACCTG
chr1	2.05E+08	-	7	GAAGGGACCTCGGTATCCACCTCATTCTGCTTCCT	GA*GTGAC*TCGAT*TCCTC*CCATT*GCCTG<br>|||| ||||||| ||||| || ||||| || GAAGGGACCTCGGTATCCACCTCATTCTGCTTCCT
chr2	3545252	+	7	AAGCAGGCACATGGGGCTGAGACCCAGCACACATC	CAGGC*AATGG*GAGGA*ATCGA*GTCAC*TC<br>|||||| |||||| |||| | || |||||| AAGCAGGCACATGGGGCTGAGACCCAGCACACATC
chr2	28624549	+	7	GAGATGGGGAATGGTGCGGAAATCTAAGTAACCAC	CAGGC*AATGG*GAGGA*ATCGA*GTCAC*TC<br>|| |||||||| ||||||| |||| ||| | GAGATGGGGAATGGTGCGGAAATCTAAGTAACCAC
chr2	2.17E+08	+	7	GAGCATGCCAAGGGAGAAGCCATGGAGATCACATG	CAGGC*AATGG*GAGGA*ATCGA*GTCAC*TC<br>|| ||||| ||||| | ||| ||| |||||| GAGCATGCCAAGGGAGAAGCCATGGAGATCACATG
chr3	12357503	-	7	CAGGGGAATTACATATCCTCCCCATTGCCCTTCCT	GA*GTGAC*TCGAT*TCCTC*CCATT*GCCTG<br>||| || || ||||||||||||||| ||| CAGGGGAATTACATATCCTCCCCATTGCCCTTCCT
chr3	30707775	+	7	TAGAAGGATAATGGTGATGATATTTAGGTGAAATC	CAGGC*AATGG*GAGGA*ATCGA*GTCAC*TC<br>||| ||||||||| ||||| |||| | ||| TAGAAGGATAATGGTGATGATATTTAGGTGAAATC
chr3	37961206	-	7	GAAATGACATCTTTATCCTTACCATTAGTATCGTT	GA*GTGAC*TCGAT*TCCTC*CCATT*GCCTG<br>||| ||||||| |||||| |||||||| | GAAATGACATCTTTATCCTTACCATTAGTATCGTT
chr4	1.51E+08	+	7	AGGCAGGCAGATGGTCAGGAGATCGAGACCATCCT	CAGGC*AATGG*GAGGA*ATCGA*GTCAC*TC<br>|||||| ||||| ||||||||||| || | AGGCAGGCAGATGGTCAGGAGATCGAGACCATCCT
chr4	1.8E+08	-	7	GAAGTAGCATCGGTCTTCTCTCCCATGGCCTTCAT	GA*GTGAC*TCGAT*TCCTC*CCATT*GCCTG<br>||||| ||||| ||| |||||| |||||| GAAGTAGCATCGGTCTTCTCTCCCATGGCCTTCAT
chr6	3850343	+	7	GAGAAAGTGAAGGACGAGGAGATGGAGGTCACCTT	CAGGC*AATGG*GAGGA*ATCGA*GTCAC*TC<br>| | ||| | ||||||||| |||||||||| GAGAAAGTGAAGGACGAGGAGATGGAGGTCACCTT
chr6_GL000252v2_alt	3097109	-	7	GCTCTGACTTCCAGGTTCACTCCATTCTCCTGCTT	GA*GTGAC*TCGAT*TCCTC*CCATT*GCCTG<br>| | ||||||| | || | |||||||| |||| GCTCTGACTTCCAGGTTCACTCCATTCTCCTGCTT
chr7	34364205	+	7	AAGCAGGCAAATAGAAACTACCACGATGGCACCTC	CAGGC*AATGG*GAGGA*ATCGA*GTCAC*TC<br>||||||||| || | || ||||| |||||| AAGCAGGCAAATAGAAACTACCACGATGGCACCTC
chr7	1.27E+08	+	7	AGGCAGGCGAATCACGAGGAGATCGAGACCATCCT	CAGGC*AATGG*GAGGA*ATCGA*GTCAC*TC<br>||||||||| ||||||||||||| || | AGGCAGGCGAATCACGAGGAGATCGAGACCATCCT
chr8	60203828	-	7	AAATTCACTTCATTTTCCTCCTCATTTACCTGCTT	GA*GTGAC*TCGAT*TCCTC*CCATT*GCCTG<br>|| | ||||| |||||||| ||||| |||| AAATTCACTTCATTTTCCTCCTCATTTACCTGCTT
chr8	62122630	+	7	AACCAGGTCAAAACTGAGGAGATCTAGGTAACACC	CAGGC*AATGG*GAGGA*ATCGA*GTCAC*TC<br>|||| ||| |||||||||| |||| ||| | AACCAGGTCAAAACTGAGGAGATCTAGGTAACACC
chr8	1.36E+08	-	7	GCTCTGCCTTCCAGGTCCACGCCATTCTCCTGCCT	GA*GTGAC*TCGAT*TCCTC*CCATT*GCCTG<br>| | || |||| | |||| |||||||| |||| GCTCTGCCTTCCAGGTCCACGCCATTCTCCTGCCT
chr9	1.29E+08	-	7	GCTCCGAGTTCCATGTTCTCGCCATTCTCCTGCCT	GA*GTGAC*TCGAT*TCCTC*CCATT*GCCTG<br>| | || ||| |||| |||||||||| |||| GCTCCGAGTTCCATGTTCTCGCCATTCTCCTGCCT
chr10	49015638	-	7	GGAGTGGGGTCAATGTCCCCGCCATGTGGCTGCCT	GA*GTGAC*TCGAT*TCCTC*CCATT*GCCTG<br>| |||| ||| |||||| |||||| || ||| GGAGTGGGGTCAATGTCCCCGCCATGTGGCTGCCT
chr10	83776992	+	7	TAGGAGGCCAAGGGGGAGGATCACGAGGTCAGGAG	CAGGC*AATGG*GAGGA*ATCGA*GTCAC*TC<br>||||||| ||||||||| |||||||| | TAGGAGGCCAAGGGGGAGGATCACGAGGTCAGGAG
chr10	1.22E+08	-	7	GATGTGGCATGGATATCCTGTGGCTTTGCCTACCT	GA*GTGAC*TCGAT*TCCTC*CCATT*GCCTG<br>|||||| ||| |||||||| | ||||||| GATGTGGCATGGATATCCTGTGGCTTTGCCTACCT
chr11	64452563	+	7	AGGCAGGAGAATGGTGTGAACCTGGAAGGCAGATC	CAGGC*AATGG*GAGGA*ATCGA*GTCAC*TC<br>|||| |||||||| | || | |||| || ||| AGGCAGGAGAATGGTGTGAACCTGGAAGGCAGATC
chr11	67298143	-	7	GCTCTGCCTCCGATGTTCACGCCATTCTCCTGCCT	GA*GTGAC*TCGAT*TCCTC*CCATT*GCCTG<br>| | || || |||||| | |||||||| |||| GCTCTGCCTCCGATGTTCACGCCATTCTCCTGCCT
chr12	79723293	-	7	GCTCTGCCTTCCATGTTCACGCCATTCTCCTGCCT	GA*GTGAC*TCGAT*TCCTC*CCATT*GCCTG<br>| | || |||| |||| | |||||||| |||| GCTCTGCCTTCCATGTTCACGCCATTCTCCTGCCT
chr14	60874375	-	7	CTGGTAATATCGCTGTCCTCCCCATTGCCTTGCTT	GA*GTGAC*TCGAT*TCCTC*CCATT*GCCTG<br>||| | |||| |||||||||||||| | || CTGGTAATATCGCTGTCCTCCCCATTGCCTTGCTT
chr15	37276144	+	7	AGGCAGGAAAACAGGGAAGAGATACTAGTCACATC	CAGGC*AATGG*GAGGA*ATCGA*GTCAC*TC<br>|||| ||| |||| ||||| ||||||||| AGGCAGGAAAACAGGGAAGAGATACTAGTCACATC
chr15	49852972	+	7	ATGCAGGGTAATGATGGGGAAACAGACCTCAGGTC	CAGGC*AATGG*GAGGA*ATCGA*GTCAC*TC<br>|||| ||||| || ||||| ||| ||| ||| ATGCAGGGTAATGATGGGGAAACAGACCTCAGGTC
chr15	96162125	-	7	AGAGTGACACCCATTTCCTGTCCTTTTGCATGGTT	GA*GTGAC*TCGAT*TCCTC*CCATT*GCCTG<br>||||||| | ||||||| ||| ||||| || AGAGTGACACCCATTTCCTGTCCTTTTGCATGGTT
chr15	97108135	+	7	TAGCAGGCAAATAGAGAGAAAGGCCAGTTCACCTA	CAGGC*AATGG*GAGGA*ATCGA*GTCAC*TC<br>||||||||| ||||| || | || |||||| TAGCAGGCAAATAGAGAGAAAGGCCAGTTCACCTA
chr17	16860619	-	7	AATGTGACCCAGATAGCTTCCCCCTTACCCTGCTC	GA*GTGAC*TCGAT*TCCTC*CCATT*GCCTG<br>|||||||| |||| | ||||| ||| |||| AATGTGACCCAGATAGCTTCCCCCTTACCCTGCTC
chr17	32192881	-	7	AGGGTGGACTCGATCTCCTCGTGATCCGCCTGCCT	GA*GTGAC*TCGAT*TCCTC*CCATT*GCCTG<br>|||| ||||||||||||| || |||||| AGGGTGGACTCGATCTCCTCGTGATCCGCCTGCCT
chr18	68427012	+	7	AACCAGGAGAATGTCGAGAAAATTGTTGTCAGATA	CAGGC*AATGG*GAGGA*ATCGA*GTCAC*TC<br>|||| ||||| |||| |||| | ||||| || AACCAGGAGAATGTCGAGAAAATTGTTGTCAGATA
chr22	48541378	-	7	GCAGGGACTTGGAGACCCTCCCCATTCCCATGCTT	GA*GTGAC*TCGAT*TCCTC*CCATT*GCCTG<br>| || ||||| || | ||||||||||| | || GCAGGGACTTGGAGACCCTCCCCATTCCCATGCTT
chrX	16884110	-	7	AAACTAACATTGATGTTCCCACCATTGGCCAGCTT	GA*GTGAC*TCGAT*TCCTC*CCATT*GCCTG<br>|| | |||| ||||| | ||||||||||| | AAACTAACATTGATGTTCCCACCATTGGCCAGCTT

Supplementary Table 8. List of potential off-target sites by mismatch detection with the on-target *CCR5* site.

<table>
<tr><th>chromosome</th><th>position</th><th>strand</th><th># of mismatch</th><th>off-target sequence</th><th>alignment</th></tr>
<tr><td>chr3 (On-target)</td><td>46372920</td><td>+</td><td>0</td><td>AAGTCCAATCTATGACATCAATTATTATACATCGG</td><td>   TCCAA*CTATG*CATCA*TTATT*TACAT*GG
   ||||||||||||||||||||||||||||||||
AAGTCCAATCTATGACATCAATTATTATACATCGG</td></tr>
<tr><td>chr6</td><td>1.36E+08</td><td>+</td><td>5</td><td>AGGTCCAAAGTTTTCCATCATTTTTTCTACATGAG</td><td>   TCCAA*CTATG*CATCA*TTATT*TACAT*GG
   |||||| | | ||||||||| ||||||||| |
AGGTCCAAAGTTTTCCATCATTTTTTCTACATGAG</td></tr>
<tr><td>chr1</td><td>768669</td><td>-</td><td>6</td><td>CTAACGTATAATGACTGATGTCATATATTTTACAT</td><td>CC*ATGTA*AATAA*TGATG*CATAG*TTGGA
| || ||||||| |||||||||||| ||| |
CTAACGTATAATGACTGATGTCATATATTTTACAT</td></tr>
<tr><td>chr2</td><td>24156702</td><td>+</td><td>6</td><td>ATGTCCAATCTATAATAACATTTATTATTTATTGA</td><td>   TCCAA*CTATG*CATCA*TTATT*TACAT*GG
   |||||||||| | | |||||||||| ||||
ATGTCCAATCTATAATAACATTTATTATTTATTGA</td></tr>
<tr><td>chr3</td><td>42026877</td><td>-</td><td>6</td><td>CATATTTATAGTAACCCATGTCTTAGCTTGGAGTT</td><td>CC*ATGTA*AATAA*TGATG*CATAG*TTGGA
| ||| |||| |||| ||||| |||||||||
CATATTTATAGTAACCCATGTCTTAGCTTGGAGTT</td></tr>
<tr><td>chr3</td><td>1.23E+08</td><td>+</td><td>6</td><td>TAGTCTAACTGATGACATCATTTCTTCTACAGATG</td><td>   TCCAA*CTATG*CATCA*TTATT*TACAT*GG
   || ||| |||||||||||| ||||||| | |
TAGTCTAACTGATGACATCATTTCTTCTACAGATG</td></tr>
<tr><td>chr9</td><td>1.31E+08</td><td>+</td><td>6</td><td>TAGTCCCAGCTCTGCCATCAGTCATGTGACCTTGG</td><td>   TCCAA*CTATG*CATCA*TTATT*TACAT*GG
   ||| |||| |||||||||| || | || ||||
TAGTCCCAGCTCTGCCATCAGTCATGTGACCTTGG</td></tr>
<tr><td>chr10</td><td>35327369</td><td>+</td><td>6</td><td>TAGTCCACTGTATGTAATTAGTTATCATTCATGGG</td><td>   TCCAA*CTATG*CATCA*TTATT*TACAT*GG
   |||| | ||||| || |||||| || ||||||
TAGTCCACTGTATGTAATTAGTTATCATTCATGGG</td></tr>
<tr><td>chr17</td><td>12236253</td><td>-</td><td>6</td><td>GTTATTTACAATAAGTGATGAAATAGTTTGTCCTT</td><td>CC*ATGTA*AATAA*TGATG*CATAG*TTGGA
  ||| ||||||||||||||| ||||||||
GTTATTTACAATAAGTGATGAAATAGTTTGTCCTT</td></tr>
<tr><td>chr17</td><td>64580400</td><td>-</td><td>6</td><td>TATATGTCTAAAAAATTATGTCATAGGTTGAACTT</td><td>CC*ATGTA*AATAA*TGATG*CATAG*TTGGA
  ||||| ||| |||| ||||||||||||| |
TATATGTCTAAAAAATTATGTCATAGGTTGAACTT</td></tr>
<tr><td>chr19</td><td>233598</td><td>-</td><td>6</td><td>CTAACGTATAATGACTGATGTCATATATTTTACAT</td><td>CC*ATGTA*AATAA*TGATG*CATAG*TTGGA
| || ||||||| |||||||||||| ||| |
CTAACGTATAATGACTGATGTCATATATTTTACAT</td></tr>
<tr><td>chrX</td><td>42492191</td><td>-</td><td>6</td><td>AGAATGTGAAATAAGAGATGCCATATGTTTGACAT</td><td>CC*ATGTA*AATAA*TGATG*CATAG*TTGGA
  ||||| ||||||| ||||||||| ||| ||
AGAATGTGAAATAAGAGATGCCATATGTTTGACAT</td></tr>
<tr><td>chrX</td><td>1.31E+08</td><td>+</td><td>6</td><td>AACTACAACCTATGGTATGAATTATAGTAGTTTGG</td><td>   TCCAA*CTATG*CATCA*TTATT*TACAT*GG
   | |||||||||| || |||||| ||| ||||
AACTACAACCTATGGTATGAATTATAGTAGTTTGG</td></tr>
<tr><td>chr1</td><td>52940684</td><td>-</td><td>7</td><td>CCAATAAATATTAGTTGTTATCATTGTTTGGACCT</td><td>CC*ATGTA*AATAA*TGATG*CATAG*TTGGA
||||| ||| || ||| | |||| |||||||
CCAATAAATATTAGTTGTTATCATTGTTTGGACCT</td></tr>
<tr><td>chr1</td><td>95451089</td><td>+</td><td>7</td><td>AAGCCAAAAATATGCTAATAGTTATTATAAATGGG</td><td>   TCCAA*CTATG*CATCA*TTATT*TACAT*GG
    | ||| ||||| | |||||||||| |||||
AAGCCAAAAATATGCTAATAGTTATTATAAATGGG</td></tr>
<tr><td>chr1</td><td>1.75E+08</td><td>-</td><td>7</td><td>CCCATCTAAAACAAGGTTTGAAGTAGTTTGGACTT</td><td>CC*ATGTA*AATAA*TGATG*CATAG*TTGGA
||||| ||||| ||| ||| |||||||||
CCCATCTAAAACAAGGTTTGAAGTAGTTTGGACTT</td></tr>
<tr><td>chr1</td><td>2.17E+08</td><td>+</td><td>7</td><td>AAGTGCTAACTATTGCTTCTATTATATTACATTGA</td><td>   TCCAA*CTATG*CATCA*TTATT*TACAT*GG
   | | |||||| || || ||||| ||||||||
AAGTGCTAACTATTGCTTCTATTATATTACATTGA</td></tr>
<tr><td>chr1</td><td>2.24E+08</td><td>-</td><td>7</td><td>CTAACGTATAATGACTGATTTCATATATTTTACAT</td><td>CC*ATGTA*AATAA*TGATG*CATAG*TTGGA
| || ||||||| |||||| ||||| ||| |
CTAACGTATAATGACTGATTTCATATATTTTACAT</td></tr>
<tr><td>chr2</td><td>29076913</td><td>+</td><td>7</td><td>AAGACCAACTTTTGTCTTTCTTTATTTTAAATTGG</td><td>   TCCAA*CTATG*CATCA*TTATT*TACAT*GG
    ||||| | |||| | ||||||||| |||||
AAGACCAACTTTTGTCTTTCTTTATTTTAAATTGG</td></tr>
<tr><td>chr2</td><td>31432083</td><td>-</td><td>7</td><td>CACTTGTGGACTAAGTGGTCTCAGAGATTGGACTA</td><td>CC*ATGTA*AATAA*TGATG*CATAG*TTGGA
| | ||| || |||||| | ||| ||||||||
CACTTGTGGACTAAGTGGTCTCAGAGATTGGACTA</td></tr>
<tr><td>chr2</td><td>39778213</td><td>+</td><td>7</td><td>ATGTTCAATAAATGTCAGTAGTTATTATATATTGA</td><td>   TCCAA*CTATG*CATCA*TTATT*TACAT*GG
   | |||| |||||| |||||||||| ||||
ATGTTCAATAAATGTCAGTAGTTATTATATATTGA</td></tr>
<tr><td>chr2</td><td>1.85E+08</td><td>-</td><td>7</td><td>CAGTTGGAAAATAATTGTTTATATAGATAGGACTC</td><td>CC*ATGTA*AATAA*TGATG*CATAG*TTGGA
| | || |||||||||| | | |||||| |||
CAGTTGGAAAATAATTGTTTATATAGATAGGACTC</td></tr>
<tr><td>chr3</td><td>18389527</td><td>-</td><td>7</td><td>CCCATATCCTATAATTGAAGACATAGTACAGACTT</td><td>CC*ATGTA*AATAA*TGATG*CATAG*TTGGA
||||| | | |||||||| |||||||| ||
CCCATATCCTATAATTGAAGACATAGTACAGACTT</td></tr>
<tr><td>chr3</td><td>37825552</td><td>+</td><td>7</td><td>AAGTCCAAATTATTTTATATTTTATTTTAAATCAG</td><td>   TCCAA*CTATG*CATCA*TTATT*TACAT*GG
   |||||| ||| | || ||||||||| ||| |
AAGTCCAAATTATTTTATATTTTATTTTAAATCAG</td></tr>
<tr><td>chr3</td><td>64232991</td><td>+</td><td>7</td><td>ATGTGCTATCTATGGCTGCATTCATGATACAATGG</td><td>   TCCAA*CTATG*CATCA*TTATT*TACAT*GG
   | | ||||||||| |||| || ||||| |||
ATGTGCTATCTATGGCTGCATTCATGATACAATGG</td></tr>
<tr><td>chr3</td><td>70316563</td><td>+</td><td>7</td><td>ATGACCAATATATACCATCAAATATTGCACATTAA</td><td>   TCCAA*CTATG*CATCA*TTATT*TACAT*GG
    ||||| ||| ||||||| ||||| |||||
ATGACCAATATATACCATCAAATATTGCACATTAA</td></tr>
<tr><td>chr3</td><td>86522143</td><td>-</td><td>7</td><td>ATAAAGTTAAAACACTGATTACATAGATTGGACCT</td><td>CC*ATGTA*AATAA*TGATG*CATAG*TTGGA
  || || ||| |||||| ||||||||||||
ATAAAGTTAAAACACTGATTACATAGATTGGACCT</td></tr>
<tr><td>chr3</td><td>88693070</td><td>-</td><td>7</td><td>CCAATGTAAAAGAACTGAAATCATAGCAAACAGTT</td><td>CC*ATGTA*AATAA*TGATG*CATAG*TTGGA
||||||||||| |||||| ||||||| |
CCAATGTAAAAGAACTGAAATCATAGCAAACAGTT</td></tr>
<tr><td>chr3</td><td>1.41E+08</td><td>-</td><td>7</td><td>CCATTGAGTAATATTTCATGGTATAGATTAGACAT</td><td>CC*ATGTA*AATAA*TGATG*CATAG*TTGGA
||| || ||||| || |||| ||||||| ||
CCATTGAGTAATATTTCATGGTATAGATTAGACAT</td></tr>
<tr><td>chr3</td><td>1.51E+08</td><td>-</td><td>7</td><td>CCCAGCTCTAATATATGCTTGCATAAGTTGGACCT</td><td>CC*ATGTA*AATAA*TGATG*CATAG*TTGGA
|||| | ||||| ||| | ||||| ||||||
CCCAGCTCTAATATATGCTTGCATAAGTTGGACCT</td></tr>
<tr><td>chr3</td><td>1.6E+08</td><td>+</td><td>7</td><td>TAGTCCAAACTTCTGAGTAAATTAATTTACATAGG</td><td>   TCCAA*CTATG*CATCA*TTATT*TACAT*GG
   |||||||| | | ||||| ||||||||||
TAGTCCAAACTTCTGAGTAAATTAATTTACATAGG</td></tr>
<tr><td>chr3</td><td>1.72E+08</td><td>+</td><td>7</td><td>AACTCCATTCTATGTCTTCCTTTATTCCATACCAG</td><td>   TCCAA*CTATG*CATCA*TTATT*TACAT*GG
   |||| |||||||| || ||||||| | | | |
AACTCCATTCTATGTCTTCCTTTATTCCATACCAG</td></tr>
<tr><td>chr3</td><td>1.73E+08</td><td>+</td><td>7</td><td>AGGTCTAAGGTTTGATCTTATTTATTCTACTTAGG</td><td>   TCCAA*CTATG*CATCA*TTATT*TACAT*GG
   || ||| | ||| | ||||||||||| ||||
AGGTCTAAGGTTTGATCTTATTTATTCTACTTAGG</td></tr>
<tr><td>chr3</td><td>1.82E+08</td><td>+</td><td>7</td><td>AACTCCACTCTATGTATTCACTTATTCACTATTCG</td><td>   TCCAA*CTATG*CATCA*TTATT*TACAT*GG
   |||| ||||||| |||||||||| ||| |
AACTCCACTCTATGTATTCACTTATTCACTATTCG</td></tr>
<tr><td>chr3</td><td>1.98E+08</td><td>+</td><td>7</td><td>ATGTAAAATATATGAAATCAGTCATTATACGTTAG</td><td>   TCCAA*CTATG*CATCA*TTATT*TACAT*GG
   | ||| ||||| |||||| ||||||| || |
ATGTAAAATATATGAAATCAGTCATTATACGTTAG</td></tr>
<tr><td>chr3_KI270784v1_alt</td><td>49167</td><td>-</td><td>7</td><td>CTAACGTATAATGACTGATTTCATATATTTTACAT</td><td>CC*ATGTA*AATAA*TGATG*CATAG*TTGGA
| || ||||||| |||||| ||||| ||| |
CTAACGTATAATGACTGATTTCATATATTTTACAT</td></tr>
<tr><td>chr4</td><td>1912363</td><td>-</td><td>7</td><td>TAAATGAAAAATAAGTGTTTACATAATTTTGACTA</td><td>CC*ATGTA*AATAA*TGATG*CATAG*TTGGA
  |||| |||||||||| | ||||| ||| ||
TAAATGAAAAATAAGTGTTTACATAATTTTGACTA</td></tr>
<tr><td>chr4</td><td>21419195</td><td>+</td><td>7</td><td>TAGTCCAAGCTCTAGCATCAAGCATGGTACTTGGA</td><td>   TCCAA*CTATG*CATCA*TTATT*TACAT*GG
   |||||||| | ||||||| || |||| |||
TAGTCCAAGCTCTAGCATCAAGCATGGTACTTGGA</td></tr>
</table>

<table>
<tr><td></td><td></td><td></td><td></td><td></td><td></td></tr>
<tr><td>chr4</td><td>35959440</td><td>+</td><td>7</td><td>AAGTTCATGGTATTACATTAGGTGTTGTACATTGG</td><td>   TCCAA*CTATG*CATCA*TTATT*TACAT*GG
   | || | ||| |||| || | |||||||||||
AAGTTCATGGTATTACATTAGGTGTTGTACATTGG</td></tr>
<tr><td>chr4</td><td>64580238</td><td>-</td><td>7</td><td>CAGATGTATCATAGTTTTTGACATAAAATGGACAT</td><td>CC*ATGTA*AATAA*TGATG*CATAG*TTGGA
| ||||||| ||| || ||||||| | ||||
CAGATGTATCATAGTTTTTGACATAAAATGGACAT</td></tr>
<tr><td>chr4</td><td>1.67E+08</td><td>-</td><td>7</td><td>ATGATGTACTGTAATTGATGGTAAAGTGTGGAGTT</td><td>CC*ATGTA*AATAA*TGATG*CATAG*TTGGA
  ||||||| |||||||||| | ||| ||||
ATGATGTACTGTAATTGATGGTAAAGTGTGGAGTT</td></tr>
<tr><td>chr5</td><td>13292983</td><td>-</td><td>7</td><td>CCCATGTAAATTAAATCATGCAGTATATAGTACAT</td><td>CC*ATGTA*AATAA*TGATG*CATAG*TTGGA
|||||||||| ||||| |||| || || | |
CCCATGTAAATTAAATCATGCAGTATATAGTACAT</td></tr>
<tr><td>chr5</td><td>23200932</td><td>+</td><td>7</td><td>AAGTAAAATGTATGACATCACTGATAATACAAGGA</td><td>   TCCAA*CTATG*CATCA*TTATT*TACAT*GG
   | ||| |||||||||||| || ||||| ||
AAGTAAAATGTATGACATCACTGATAATACAAGGA</td></tr>
<tr><td>chr5</td><td>28896980</td><td>-</td><td>7</td><td>CAATTGAAAAATAATGATTGCCATAGACTGGAGTT</td><td>CC*ATGTA*AATAA*TGATG*CATAG*TTGGA
| | || |||||||| ||||||||| ||||
CAATTGAAAAATAATGATTGCCATAGACTGGAGTT</td></tr>
<tr><td>chr5</td><td>1.23E+08</td><td>-</td><td>7</td><td>TTAATGTAGAATAATTGTGGCTTTACCTTGGACTT</td><td>CC*ATGTA*AATAA*TGATG*CATAG*TTGGA
  ||||||||||||||| || || ||||||
TTAATGTAGAATAATTGTGGCTTTACCTTGGACTT</td></tr>
<tr><td>chr5</td><td>1.33E+08</td><td>+</td><td>7</td><td>AACTACCACCCATTGCATCACCTATTGTACTTGTG</td><td>   TCCAA*CTATG*CATCA*TTATT*TACAT*GG
   | | ||| || ||||||| |||||||| || |
AACTACCACCCATTGCATCACCTATTGTACTTGTG</td></tr>
<tr><td>chr5</td><td>1.81E+08</td><td>+</td><td>7</td><td>ATGTAAAATATATGAAATCAGTCATTATACGTTAG</td><td>   TCCAA*CTATG*CATCA*TTATT*TACAT*GG
   | ||| ||||| |||||| ||||||| || |
ATGTAAAATATATGAAATCAGTCATTATACGTTAG</td></tr>
<tr><td>chr6</td><td>4723167</td><td>-</td><td>7</td><td>CCCATGTAGTATAACTATTTACATAGCATTTACAT</td><td>CC*ATGTA*AATAA*TGATG*CATAG*TTGGA
||||||||| |||||| | ||||||| | |
CCCATGTAGTATAACTATTTACATAGCATTTACAT</td></tr>
<tr><td>chr6</td><td>33398948</td><td>-</td><td>7</td><td>CCTTTTTTACATAAATGGTGGCATACTTTAGACAT</td><td>CC*ATGTA*AATAA*TGATG*CATAG*TTGGA
||| | | | ||||||| ||||||| ||| ||
CCTTTTTTACATAAATGGTGGCATACTTTAGACAT</td></tr>
<tr><td>chr6</td><td>45195172</td><td>+</td><td>7</td><td>ATGGCCAAGTGATGCTATGATATATTATACAATGG</td><td>   TCCAA*CTATG*CATCA*TTATT*TACAT*GG
    ||||| |||| || || ||||||||| |||
ATGGCCAAGTGATGCTATGATATATTATACAATGG</td></tr>
<tr><td>chr6</td><td>1.19E+08</td><td>+</td><td>7</td><td>AACTCCCACCAGTGCCATAACATAGTTTACAAAGG</td><td>   TCCAA*CTATG*CATCA*TTATT*TACAT*GG
   ||| ||| |||||| || || |||||| |||
AACTCCCACCAGTGCCATAACATAGTTTACAAAGG</td></tr>
<tr><td>chr6</td><td>1.34E+08</td><td>+</td><td>7</td><td>AGGTCAAAACTCATCAATAACTAATTATACATTGG</td><td>   TCCAA*CTATG*CATCA*TTATT*TACAT*GG
   || ||||| | || ||| ||||||||||||
AGGTCAAAACTCATCAATAACTAATTATACATTGG</td></tr>
<tr><td>chr6</td><td>1.46E+08</td><td>-</td><td>7</td><td>TCAAACTAAAATAATTGATATCATACATTGCTCTA</td><td>CC*ATGTA*AATAA*TGATG*CATAG*TTGGA
 ||| ||||||||||||| ||||| ||||
TCAAACTAAAATAATTGATATCATACATTGCTCTA</td></tr>
<tr><td>chr6_GL000255v2_alt</td><td>4593349</td><td>-</td><td>7</td><td>CCTTTTTTACATAAATGGTGGCATACTTTAGACAT</td><td>CC*ATGTA*AATAA*TGATG*CATAG*TTGGA
||| | | | ||||||| ||||||| ||| ||
CCTTTTTTACATAAATGGTGGCATACTTTAGACAT</td></tr>
<tr><td>chr6_GL000256v2_alt</td><td>4847647</td><td>-</td><td>7</td><td>CCTTTTTTACATAAATGGTGGCATACTTTAGACAT</td><td>CC*ATGTA*AATAA*TGATG*CATAG*TTGGA
||| | | | ||||||| ||||||| ||| ||
CCTTTTTTACATAAATGGTGGCATACTTTAGACAT</td></tr>
<tr><td>chr7</td><td>87036413</td><td>+</td><td>7</td><td>AGGTCAAAACTATGTGAGCTGTTATTGTACCACTG</td><td>   TCCAA*CTATG*CATCA*TTATT*TACAT*GG
   || ||||||||| | | |||||||||| | |
AGGTCAAAACTATGTGAGCTGTTATTGTACCACTG</td></tr>
<tr><td>chr7</td><td>87549550</td><td>+</td><td>7</td><td>AAGGACAAGCTATCTCAGCTCATATTTTAAATTGG</td><td>   TCCAA*CTATG*CATCA*TTATT*TACAT*GG
     |||||||| ||| | | ||||||| |||||
AAGGACAAGCTATCTCAGCTCATATTTTAAATTGG</td></tr>
<tr><td>chr7</td><td>1.47E+08</td><td>+</td><td>7</td><td>AGGGCACAGTGTTGACATAAATTATTCTACATGGG</td><td>   TCCAA*CTATG*CATCA*TTATT*TACAT*GG
    | || |||||| ||||||||||||||||
AGGGCACAGTGTTGACATAAATTATTCTACATGGG</td></tr>
<tr><td>chr8</td><td>54956134</td><td>-</td><td>7</td><td>CACCTGTAAAATCAATGGTGGCATACTGTGTACCT</td><td>CC*ATGTA*AATAA*TGATG*CATAG*TTGGA
| | |||||||| |||| ||||||| | || |
CACCTGTAAAATCAATGGTGGCATACTGTGTACCT</td></tr>
<tr><td>chr8</td><td>62720416</td><td>+</td><td>7</td><td>ATGCCCAAACAATACCATGAATTATTAAATATCTG</td><td>   TCCAA*CTATG*CATCA*TTATT*TACAT*GG
    |||||| || |||| |||||||| | ||| |
ATGCCCAAACAATACCATGAATTATTAAATATCTG</td></tr>
<tr><td>chr8</td><td>87204376</td><td>+</td><td>7</td><td>AAGGCCAAACTATTTCATCAGACCTTTGCCATAGG</td><td>   TCCAA*CTATG*CATCA*TTATT*TACAT*GG
    ||||||||| ||||||| ||| ||||||
AAGGCCAAACTATTTCATCAGACCTTTGCCATAGG</td></tr>
<tr><td>chr8</td><td>97267469</td><td>+</td><td>7</td><td>AAGTACAAGTCATTACATCAGTTACTGTAGATTGA</td><td>   TCCAA*CTATG*CATCA*TTATT*TACAT*GG
   | |||| || |||||||||| |||| ||||
AAGTACAAGTCATTACATCAGTTACTGTAGATTGA</td></tr>
<tr><td>chr8</td><td>1.08E+08</td><td>-</td><td>7</td><td>CTAAGAAAGAAAAAATGATGTCATAAGTGGGAGTT</td><td>CC*ATGTA*AATAA*TGATG*CATAG*TTGGA
| || |||| ||||||||||||| || |||
CTAAGAAAGAAAAAATGATGTCATAAGTGGGAGTT</td></tr>
<tr><td>chr8_KI270821v1_alt</td><td>557712</td><td>+</td><td>7</td><td>GAGTCCAGAATATGACTGCACTTCTTTTTCATATG</td><td>   TCCAA*CTATG*CATCA*TTATT*TACAT*GG
   |||| | |||||| ||||| |||| |||| |
GAGTCCAGAATATGACTGCACTTCTTTTTCATATG</td></tr>
<tr><td>chr9</td><td>25010452</td><td>+</td><td>7</td><td>ATGTCTCAACAATGACATCATTGATTCTAGATTAT</td><td>   TCCAA*CTATG*CATCA*TTATT*TACAT*GG
   || ||| ||||||||||| |||||| |||
ATGTCTCAACAATGACATCATTGATTCTAGATTAT</td></tr>
<tr><td>chr9</td><td>1.03E+08</td><td>+</td><td>7</td><td>AAGAAAAATCTATGTCGTCATATATTCAACATTTG</td><td>   TCCAA*CTATG*CATCA*TTATT*TACAT*GG
      |||||||||| |||| ||||| ||||| |
AAGAAAAATCTATGTCGTCATATATTCAACATTTG</td></tr>
<tr><td>chr10</td><td>61738697</td><td>-</td><td>7</td><td>CAATTGTACAATAAATGTTGTAGTGGCTTGAACCT</td><td>CC*ATGTA*AATAA*TGATG*CATAG*TTGGA
| | ||||||||||||| ||| | ||||| |
CAATTGTACAATAAATGTTGTAGTGGCTTGAACCT</td></tr>
<tr><td>chr10</td><td>76438142</td><td>-</td><td>7</td><td>CACATGTAGAAGGAAGAATCTCATTGCTTGGACAT</td><td>CC*ATGTA*AATAA*TGATG*CATAG*TTGGA
| ||||||||| || || |||| |||||||
CACATGTAGAAGGAAGAATCTCATTGCTTGGACAT</td></tr>
<tr><td>chr10</td><td>81378913</td><td>+</td><td>7</td><td>AACTCTATACTATGACATTATTTAAAGAGCATGGG</td><td>   TCCAA*CTATG*CATCA*TTATT*TACAT*GG
   || | |||||||||| ||||| | ||||||
AACTCTATACTATGACATTATTTAAAGAGCATGGG</td></tr>
<tr><td>chr11</td><td>163417</td><td>-</td><td>7</td><td>CTAACGTATAATGACTGATTTCATATATTTTACAT</td><td>CC*ATGTA*AATAA*TGATG*CATAG*TTGGA
| || ||||||| |||||| ||||| ||| |
CTAACGTATAATGACTGATTTCATATATTTTACAT</td></tr>
<tr><td>chr11</td><td>24090006</td><td>+</td><td>7</td><td>AACTATAAAATATGGAATCACTTATTCTAGGTTTG</td><td>   TCCAA*CTATG*CATCA*TTATT*TACAT*GG
   | ||| ||||| ||||||||||||| || |
AACTATAAAATATGGAATCACTTATTCTAGGTTTG</td></tr>
<tr><td>chr11</td><td>68510675</td><td>+</td><td>7</td><td>ATGTCCATATTTTGGAGTCAACTATTCTAGATTGG</td><td>   TCCAA*CTATG*CATCA*TTATT*TACAT*GG
   |||| | | ||| |||| ||||||| |||||
ATGTCCATATTTTGGAGTCAACTATTCTAGATTGG</td></tr>
<tr><td>chr11</td><td>70304458</td><td>+</td><td>7</td><td>ATGCTCAGCCTGTGATATCCTTTATAATACATGGG</td><td>   TCCAA*CTATG*CATCA*TTATT*TACAT*GG
     || ||| ||| ||| ||||| |||||||||
ATGCTCAGCCTGTGATATCCTTTATAATACATGGG</td></tr>
<tr><td>chr11</td><td>1E+08</td><td>+</td><td>7</td><td>AAGTCTAACAAATACAATCAGTTATTATAGGTAGG</td><td>   TCCAA*CTATG*CATCA*TTATT*TACAT*GG
   || ||| || | ||||||||||||| ||||
AAGTCTAACAAATACAATCAGTTATTATAGGTAGG</td></tr>
<tr><td>chr12</td><td>31622527</td><td>-</td><td>7</td><td>TGCATGCAAAATAAATGGTTATATAGGTTGAACAT</td><td>CC*ATGTA*AATAA*TGATG*CATAG*TTGGA
  |||| |||||||||| | | |||||||| |
TGCATGCAAAATAAATGGTTATATAGGTTGAACAT</td></tr>
<tr><td>chr12</td><td>1.16E+08</td><td>-</td><td>7</td><td>CCAGTGCAAACAAAGTGATGGAAAAGCTGGGACCT</td><td>CC*ATGTA*AATAA*TGATG*CATAG*TTGGA
||| || ||| ||||||||| | |||| |||
CCAGTGCAAACAAAGTGATGGAAAAGCTGGGACCT</td></tr>
<tr><td>chr12</td><td>1.23E+08</td><td>-</td><td>7</td><td>CCGATGGAAAAGAACAGGTGGCATTTGTTGGGCCT</td><td>CC*ATGTA*AATAA*TGATG*CATAG*TTGGA
|||||| |||| ||| | |||||| |||||
CCGATGGAAAAGAACAGGTGGCATTTGTTGGGCCT</td></tr>
</table>

<table>
<tr><td>chr12</td><td>1.3E+08</td><td>-</td><td>7</td><td>TCATTGTAAGATAACTGATTTCAAATATTGAACTT</td><td>CC*ATGTA*AATAA*TGATG*CATAG*TTGGA
|| ||||| ||||||||| ||| | |||| |
TCATTGTAAGATAACTGATTTCAAATATTGAACTT</td></tr>
<tr><td>chr13</td><td>36556114</td><td>-</td><td>7</td><td>ATCTTGTAGAATAATTGGATTCATAGCTTAGACCT</td><td>CC*ATGTA*AATAA*TGATG*CATAG*TTGGA
| |||||||||||| ||||||||| ||
ATCTTGTAGAATAATTGGATTCATAGCTTAGACCT</td></tr>
<tr><td>chr13</td><td>47333412</td><td>-</td><td>7</td><td>CCCATCTAGAGAAGGTGATGTCAAAGCTAGGCCTA</td><td>CC*ATGTA*AATAA*TGATG*CATAG*TTGGA
||||| |||| | ||||||||| |||| ||
CCCATCTAGAGAAGGTGATGTCAAAGCTAGGCCTA</td></tr>
<tr><td>chr14</td><td>21863872</td><td>-</td><td>7</td><td>CAATAGTATATTATGTGATGCTAGAGTTTGGAGTT</td><td>CC*ATGTA*AATAA*TGATG*CATAG*TTGGA
| | ||||| || ||||||| | ||||||||
CAATAGTATATTATGTGATGCTAGAGTTTGGAGTT</td></tr>
<tr><td>chr14</td><td>28760887</td><td>-</td><td>7</td><td>ACTAAGTAAGAACAATGATGGGATAGTTAGGAGTT</td><td>CC*ATGTA*AATAA*TGATG*CATAG*TTGGA
||| |||| | |||||||| |||||| |||
ACTAAGTAAGAACAATGATGGGATAGTTAGGAGTT</td></tr>
<tr><td>chr14</td><td>83953008</td><td>+</td><td>7</td><td>ATGTATAAATTGTAACATCATTGATAATACATGGG</td><td>TCCAA*CTATG*CATCA*TTATT*TACAT*GG
| ||| | | |||||||| || |||||||||
ATGTATAAATTGTAACATCATTGATAATACATGGG</td></tr>
<tr><td>chr14</td><td>92818514</td><td>-</td><td>7</td><td>CCGATGTAGTCAAATTGATGACAAAGATTAATCTA</td><td>CC*ATGTA*AATAA*TGATG*CATAG*TTGGA
||||||||| ||||||||||| |||||
CCGATGTAGTCAAATTGATGACAAAGATTAATCTA</td></tr>
<tr><td>chr14</td><td>98334845</td><td>-</td><td>7</td><td>CCTAAGTAGAATAAATCATGGCCAAGGATAGCCTC</td><td>CC*ATGTA*AATAA*TGATG*CATAG*TTGGA
|||| ||||||||||| ||||| ||| | |
CCTAAGTAGAATAAATCATGGCCAAGGATAGCCTC</td></tr>
<tr><td>chr15</td><td>89082608</td><td>-</td><td>7</td><td>CCTCTGAATAATTATTGCTGACACTGATTGCACTC</td><td>CC*ATGTA*AATAA*TGATG*CATAG*TTGGA
||| || ||||| |||| ||||| ||||| |
CCTCTGAATAATTATTGCTGACACTGATTGCACTC</td></tr>
<tr><td>chr17</td><td>101230</td><td>-</td><td>7</td><td>CTAACGTATAATGACTGATTTCATATATTTTACAT</td><td>CC*ATGTA*AATAA*TGATG*CATAG*TTGGA
| || ||||||| |||||| ||||| ||| |
CTAACGTATAATGACTGATTTCATATATTTTACAT</td></tr>
<tr><td>chr17</td><td>34158785</td><td>-</td><td>7</td><td>CCAAGTTACAGTTAGTGGTGTCAGAGGTGGGACTT</td><td>CC*ATGTA*AATAA*TGATG*CATAG*TTGGA
|||| |||| | |||| ||||| |||| |||
CCAAGTTACAGTTAGTGGTGTCAGAGGTGGGACTT</td></tr>
<tr><td>chr17</td><td>34346844</td><td>-</td><td>7</td><td>CATATGTAAAATGACTTTTGACATAGCATTAACTA</td><td>CC*ATGTA*AATAA*TGATG*CATAG*TTGGA
| |||||||||| ||| ||||||||| | |
CATATGTAAAATGACTTTTGACATAGCATTAACTA</td></tr>
<tr><td>chr17</td><td>56161126</td><td>-</td><td>7</td><td>CCTCTGTAAAATAAAGATTGTAATAGTATCGACCT</td><td>CC*ATGTA*AATAA*TGATG*CATAG*TTGGA
||| ||||||||||| ||| ||||| | ||
CCTCTGTAAAATAAAGATTGTAATAGTATCGACCT</td></tr>
<tr><td>chr17_GL383563v3_alt</td><td>41230</td><td>-</td><td>7</td><td>CTAACGTATAATGACTGATTTCATATATTTTACAT</td><td>CC*ATGTA*AATAA*TGATG*CATAG*TTGGA
| || ||||||| |||||| ||||| ||| |
CTAACGTATAATGACTGATTTCATATATTTTACAT</td></tr>
<tr><td>chr18</td><td>10613683</td><td>+</td><td>7</td><td>GAGAGCAATCTATGAGATGAGTTATTGAAATTAGG</td><td>TCCAA*CTATG*CATCA*TTATT*TACAT*GG
|||||||||| || |||||||| | ||||
GAGAGCAATCTATGAGATGAGTTATTGAAATTAGG</td></tr>
<tr><td>chr18</td><td>12221941</td><td>-</td><td>7</td><td>CCTAATTTCAATAACTAATCTCATAGATTGCTCTC</td><td>CC*ATGTA*AATAA*TGATG*CATAG*TTGGA
|||| | |||||||| || ||||||||||
CCTAATTTCAATAACTAATCTCATAGATTGCTCTC</td></tr>
<tr><td>chr18</td><td>24724692</td><td>+</td><td>7</td><td>AACTGCAATGTATTTATTCATTTATTGTTCATTGA</td><td>TCCAA*CTATG*CATCA*TTATT*TACAT*GG
| |||| ||| | ||||||||||| |||||
AACTGCAATGTATTTATTCATTTATTGTTCATTGA</td></tr>
<tr><td>chr18</td><td>30638615</td><td>+</td><td>7</td><td>ATGTACAATTTATGGCATGATTGATTGTTCAATGT</td><td>TCCAA*CTATG*CATCA*TTATT*TACAT*GG
| |||| |||||||| ||| ||||| || ||
ATGTACAATTTATGGCATGATTGATTGTTCAATGT</td></tr>
<tr><td>chr18</td><td>33015679</td><td>+</td><td>7</td><td>TAGGCAAAAATTTGAAATCAGTTATTATAAATATG</td><td>TCCAA*CTATG*CATCA*TTATT*TACAT*GG
| ||| | ||| ||||||||||||| ||| |
TAGGCAAAAATTTGAAATCAGTTATTATAAATATG</td></tr>
<tr><td>chr19</td><td>22588998</td><td>-</td><td>7</td><td>CTTATGGATAATTAAGGTTGTAATATTTTGGACAT</td><td>CC*ATGTA*AATAA*TGATG*CATAG*TTGGA
| |||| ||||| || | ||| ||| ||||||
CTTATGGATAATTAAGGTTGTAATATTTTGGACAT</td></tr>
<tr><td>chr19</td><td>40096786</td><td>+</td><td>7</td><td>AGGTACAAATTATGACATCAATAATGTAAAATATG</td><td>TCCAA*CTATG*CATCA*TTATT*TACAT*GG
| |||| |||||||||||| || | | ||| |
AGGTACAAATTATGACATCAATAATGTAAAATATG</td></tr>
<tr><td>chr19</td><td>49085027</td><td>-</td><td>7</td><td>TCGATGAATAGTTATTGATTGAATAGATTAGACAT</td><td>CC*ATGTA*AATAA*TGATG*CATAG*TTGGA
||||| ||| | |||||| | ||||||| ||
TCGATGAATAGTTATTGATTGAATAGATTAGACAT</td></tr>
<tr><td>chr20</td><td>18622303</td><td>+</td><td>7</td><td>AACTCCCACTTATGACATCTTTTTAAATACATTGA</td><td>TCCAA*CTATG*CATCA*TTATT*TACAT*GG
||| || ||||||||| ||| ||||||||
AACTCCCACTTATGACATCTTTTTAAATACATTGA</td></tr>
<tr><td>chr21</td><td>36040442</td><td>+</td><td>7</td><td>AACTCCAATGTATGACATCCCTTTTTCCTCCCTGG</td><td>TCCAA*CTATG*CATCA*TTATT*TACAT*GG
|||||| ||||||||| ||| ||| | |||
AACTCCAATGTATGACATCCCTTTTTCCTCCCTGG</td></tr>
<tr><td>chr21</td><td>42814200</td><td>-</td><td>7</td><td>ACTAAATAAACCAAATTATGTCATTGTTTGGACTT</td><td>CC*ATGTA*AATAA*TGATG*CATAG*TTGGA
||| |||| |||| ||||||| |||||||
ACTAAATAAACCAAATTATGTCATTGTTTGGACTT</td></tr>
<tr><td>chr22</td><td>45739215</td><td>-</td><td>7</td><td>CCCATTTACATTTAGTGAACACATAGAATGGTGTT</td><td>CC*ATGTA*AATAA*TGATG*CATAG*TTGGA
||||| |||| | ||||| ||||||| |||
CCCATTTACATTTAGTGAACACATAGAATGGTGTT</td></tr>
<tr><td>chr22</td><td>47790803</td><td>-</td><td>7</td><td>CTTATTTGAAATAATTGATGAAATAGATATCACTC</td><td>CC*ATGTA*AATAA*TGATG*CATAG*TTGGA
| ||| | ||||||||||||| |||||| |
CTTATTTGAAATAATTGATGAAATAGATATCACTC</td></tr>
<tr><td>chrX</td><td>5121860</td><td>-</td><td>7</td><td>TTTCTATGAAATAATTAATGGCATAAATTGGACTA</td><td>CC*ATGTA*AATAA*TGATG*CATAG*TTGGA
| | | |||||||| |||||||| ||||||
TTTCTATGAAATAATTAATGGCATAAATTGGACTA</td></tr>
<tr><td>chrX</td><td>14015352</td><td>+</td><td>7</td><td>AACTGTAAACTTTCTCATAAGTTATTTTCCATTGA</td><td>TCCAA*CTATG*CATCA*TTATT*TACAT*GG
| ||||| | |||| ||||||||| |||||
AACTGTAAACTTTCTCATAAGTTATTTTCCATTGA</td></tr>
<tr><td>chrX</td><td>26690203</td><td>-</td><td>7</td><td>AAAGAGTAAAATATATGATGGCACAGCTTGGCCCT</td><td>CC*ATGTA*AATAA*TGATG*CATAG*TTGGA
| |||||||| ||||||||| |||||||
AAAGAGTAAAATATATGATGGCACAGCTTGGCCCT</td></tr>
<tr><td>chrX</td><td>41885531</td><td>-</td><td>7</td><td>CATATATAAAATAAGTGTTAACAAACTTTGAACCT</td><td>CC*ATGTA*AATAA*TGATG*CATAG*TTGGA
| ||| ||||||||||| | ||| | |||| |
CATATATAAAATAAGTGTTAACAAACTTTGAACCT</td></tr>
<tr><td>chrX</td><td>84430491</td><td>+</td><td>7</td><td>ATGTCTCTTCTATGAAATGATTTATTTTCCTTTGG</td><td>TCCAA*CTATG*CATCA*TTATT*TACAT*GG
|| ||||||| || ||||||||| | ||||
ATGTCTCTTCTATGAAATGATTTATTTTCCTTTGG</td></tr>
<tr><td>chrX</td><td>87053322</td><td>+</td><td>7</td><td>TAGACAAAGATATTGAATCAGTCATTTTACATATG</td><td>TCCAA*CTATG*CATCA*TTATT*TACAT*GG
| ||| ||| | |||||| |||||||||| |
TAGACAAAGATATTGAATCAGTCATTTTACATATG</td></tr>
<tr><td>chrX</td><td>1.21E+08</td><td>-</td><td>7</td><td>CCTATGCAGAATAATATATTGCCTAGCTTTGGCAT</td><td>CC*ATGTA*AATAA*TGATG*CATAG*TTGGA
|||||| |||||||| || || |||||| |
CCTATGCAGAATAATATATTGCCTAGCTTTGGCAT</td></tr>
<tr><td>chrX</td><td>1.22E+08</td><td>+</td><td>7</td><td>ATGACCAAACCAGGACATTGCTTATTATAAATGTG</td><td>TCCAA*CTATG*CATCA*TTATT*TACAT*GG
|||||| | ||||| ||||||||| ||| |
ATGACCAAACCAGGACATTGCTTATTATAAATGTG</td></tr>
<tr><td>chrX</td><td>1.56E+08</td><td>-</td><td>7</td><td>CCGAGGAAGAAAAAAATGGTTCCATGGGTTGGGCCT</td><td>CC*ATGTA*AATAA*TGATG*CATAG*TTGGA
|||| | |||| ||||| | |||| ||||||
CCGAGGAAGAAAAAAATGGTTCCATGGGTTGGGCCT</td></tr>
<tr><td>chrY</td><td>57170003</td><td>-</td><td>7</td><td>CCGAGGAAGAAAAAAATGGTTCCATGGGTTGGGCCT</td><td>CC*ATGTA*AATAA*TGATG*CATAG*TTGGA
|||| | |||| ||||| | |||| ||||||
CCGAGGAAGAAAAAAATGGTTCCATGGGTTGGGCCT</td></tr>
</table>

Supplementary Table 9. Target sequences for Type 1-E CRISPR and CRISPR-Cas9

Name	PAM (5' to 3')	Sequence (5' to 3')	Chromosomal loci (hg19)
1-E CRISPR-Cas3			
EMX1-AAG-1	AAG	CAGGCCAATGGGGAGGACATCGATGTCACCTC	chr2:73161042-73161073
EMX1-ATG-2	ATG	GGGAGGACATCGATGTCACCTCCAATGACTAG	chr2:73161052-73161083
EMX1-TTT-3	TTT	GGGGAGGCCTGGAGTCATGGCCCCACAGGGCT	chr2:73161185-73161216
CCR5-AAG-1	AAG	TCCAATCTATGACATCAATTATTATACATCGG	chr3:46414414-46414445
CCR5-ATG-2	ATG	GATTATCAAGTGTCAAGTCCAATCTATGACAT	chr3:46414397-46414428
CCR5-TTT-3	TTT	GGTTTTGTGGGCAACATGCTGGTCATCCTCAT	chr3:46414523-46414554
EMX1-Amp	AAG	CCCATAGCCTGCACATCTCACCTAGGCCGGCC	chr2:73158802-73158834
CCR5-Amp	AAG	AGAGTCAGCCTGTCCTGTGGAGCTTTGCAGCT	chr3:46405702-46405734
TET2-Amp	AAG	AGTGCCACTTGGTGTCTCCATTTACTTCTGGA	chr4:106155233-106155265
DMD-Amp	AAG	GAAGACTTGGCCTTTATTTACCAAATGAGACT	chrX:31985736-31985768
B2M-Amp	AAG	GCCACGGAGCGAGACATCTCGGCCCGAATGCT	chr15:45003729-45003761
AAVS1-Amp	AAG	GGAAGAATGAGAAACGGTGGCCCGTGTCAGCC	chr19:55624832-55624864
VEGFA-Amp	AAG	GAATTAGGCCATCCACCCATCCCCTGAGAGGA	chr6:43742163-43742195
ERCC4-Amp	AAG	ATGATAAACCCTTGCTCGAGCATTTATAAACA	chr16:14026043-14026075
EMX1-capture-2	AAG	CTGGACTCTGGCCACTCCCTGGCCAGGCTTTG	chr2:73161153-73161185
EMX1-capture-3	AAG	CGGATCCGCACGGCCTTCTCGCCCTCGCAGCT	chr2:73151496-73151528
EMX1-capture-4	AAG	TTAGCTGCTACCCGCGTGTATCCCCATGAACC	chr2:73060167-73060199
EMX1-capture-5	AAG	TCCTGCGGGCTCAGCAGGCTGCACGCAGTCTC	chr2:73151791-73151823
EMX1-capture-6	AAG	AGGAGAGATTGGCAATGTGGGTATGGTGGAAG	chr2:73060251-73060283
CCR5-capture-2	AAG	GCTGAAGAGCATGACTGACATCTACCTGCTCA	chr3:46414572-46414604
CCR5-capture-3	AAG	TAGGACCCAATGTGACAGTTCCTGCCCAGTTC	chr3:46407278-46407310
CCR5-capture-4	AAG	GCACAGTGGCACAAAAGTGACCCACAGATGCC	chr3:46312482-46312514
CCR5-capture-5	AAG	TGGGCAGGATTGGTGGGGAAGCGAGTGAGGGC	chr3:46407530-46407562
CCR5-capture-6	AAG	AATGGTGACCCTAACTACACTGGGGATTTACC	chr3:46312795-46312827
GFP-1	AAG	GACGACGGCAACTAGAAGACCCGCGCCGAGGT	-
GFP-2	AAG	GAGCCCGCGTGGTTCCTGGCCACCGTCGGCGT	-
GFP-3	AAG	ACCTTGATCTTAACCTGGGTGATGAGGTCTCG	-
B2M-1	AAG	AGGAAGTGGACCATGGCGATGGGGAGGGGCAG	chr15:45,002,511-45,002,543
B2M-2	AAG	CCAGCGACGCAGTGCCAGGTTAGAGAGAGGGA	chr15:45,003,595-45,003,627
B2M-2'	AAG	TCCCTCTCTCTAACCTGGCACTGCGTCGCTGG	chr15:45,003,595-45,003,627
B2M-3	AAG	GCCACGGAGCGAGACATCTCGGCCCGAATGCT	chr15:45,003,729-45,003,761
B2M-4	AAG	AGAGAGTAGCGCGAGCACAGCTAAGGCCACGG	chr15:45,003,754-45,003,786
B2M-5	AAG	GGGGTGCGCACCCGGGACGCGCGCTACTTGCC	chr15:45,003,991-45,004,023
B2M-6	AAG	TATCTTGGGGCCAAATCATGTAGACTCTTGAG	chr15:45,007,220-45,007,252
B2M-6'	AAG	AGTCTACATGATTTGGCCCCAAGATACTTTTC	chr15:45,007,214-45,007,246
B2M-7	AAG	ATAGTTAAGTGGGGTAAGTCTTACATTCTTTT	chr15:45,007,886-45,007,918
B2M-8	AAG	AATCCTACAGGGTCATGTTCCCTTCTCCTGTG	chr15:45,008,682-45,008,714
B2M-9	AAG	TTAAATGGCATAGTTGGGGTGACACAGCTGTC	chr15:45,010,519-45,010,551
B2M-9'	AAG	GCTGGCCTCCCACTAGACAGCTGTGTCACCCC	chr15:45,010,534-45,010,566
B2M-10	AAG	CAATCCTTCCGCCTCAGGCTCTAGAGTAGCTG	chr15:45,012,255-45,012,287
Cas9-B2M-1	CCC	AGGAAGTGGACCATGGCGAT	chr15:45,002,523-45,002,543
Cas9-B2M-2	CCC	GCAGTGCCAGGTTAGAGAGA	chr15:45,003,599-45,003,619
Cas9-B2M-3	CCG	GGCCACGGAGCGAGACATCT	chr15:45,003,742-45,003,762
Cas9-B2M-4	CCT	GAGTAGCGCGAGCACAGCTA	chr15:45,003,763-45,003,783
Cas9-B2M-5	CCC	GCTACTTGCCCCTTTCGGCG	chr15:45,004,013-45,004,023
Cas9-B2M-6	CCA	ACTCAAGAGTCTACATGATT	chr15:45,007,233-45,007,253
Cas9-B2M-7	CCC	CAGCCCAAGATAGTTAAGTG	chr15:45,007,877-45,007,897
Cas9-B2M-8	CCC	AGGGAAAGAAGAATCCTACA	chr15:45,008,671-45,008,691
Cas9-B2M-9	CCC	GAAGTTAAATGGCATAGTTG	chr15:45,010,515-45,010,535
Cas9-B2M-10	CCT	TCAAGCAATCCTTCCGCCTC	chr15:45,012,250-45,012,270
SLC35A2-1	AAG	CTCAATGTATGAATAGCCCCGGGATTTCCAAA	chrX:48769055-48769087
SLC35A2-2	AAG	GAAGGATCCCACAATCACTCTCAGAATGTTCTCTT	chrX:48768633-48768668
SLC35A2-3	AAG	GGTCTGACTCCTACCGGCTTACTGCCTCAGCC	chrX:48767423-48767455
SLC35A2-4	AAG	GAAGCTAAATCACATGTGAGAGATCCCTGCTACTT	chrX:48766836-48766871
SLC35A2-5	AAG	AATCAGCCATCCGTGTCTGCTAATCACAGAGC	chrX:48764032-48764064
SLC35A2-6	AAG	CACCAATCAAGGGCTCTTCACAGGTATTATTTCTT	chrX:48763425-48763460
DMD-1	AAG	CTCCCTTAGACTCTCGTTTCACATACATTTCT	chrX:31,987,767-31,987,798
DMD-2	AAG	GAAGACTTGGCCTTTATTTACCAAATGAGACT	chrX:31,985,737-31,985,768
DMD-3(Nanopore-F)	AAG	CTTGTCAGCTAGAGGATGTTATAATGTCCTAC	chrX:31,986,875-31,986,906
DMD-4(Nanopore-R)	AAG	GTGAATATCTTCAATATATTTTAACTTCAACA	chrX:31,986,165-31,986,196
II-A CRISPR-Cas9			
Cas9- EMX1	CCA	CCCTAGTCATTGGAGGTGAC	chr2:73,161,066-73,161,085
Cas9- CCR5	CCG	ATGTATAATAATTGATGTCA	chr3:46,414,423-46,414,442
Cas9-EMX1-Amp	CCA	TAGCCTGCACATCTCACCTA	chr2:73158810-73158830
Cas9-CCR5-Amp	CCT	GTCCTGTGGAGCTTTGCAGC	chr3:46405703-46405723
Cas9-TET2-Amp	CCA	CTTGGTGTCTCCATTTACTT	chr4:106155238-106155258
Cas9-DMD-Amp	CCT	TTATTTACCAAATGAGACTA	chrX:31985735-31985755
Cas9-B2M-Amp	CCA	CGGAGCGAGACATCTCGGCC	chr15:45003737-45003757
Cas9-AAVS1-Amp	CCT	AAGGGAAGAATGAGAAACGG	chr19:55624847-55624867
Cas9-VEGFA-Amp	CCC	TGAGAGGACAGGGAACCCCA	chr6:43742151-43742171
Cas9-ERCC4-Amp	CCC	TTGCTCGAGCATTTATAAAC	chr16:14026044-14026064
Cas9-GFP-1	CCG	TCGTCCTTGAAGAAGATGGT	-
Cas9-GFP-2	CCA	AGGAGCCCGCGTGGTTCCTG	-
Cas9-GFP-3	CCT	GGGTGATGAGGTCTCGGTTA	-
Cas9-DMD-1	CCT	GGAGTTCCTGTAAGATACC	chrX:31,986,625-31,986,643
Cas9-DMD-2	CCT	GCTGTCAGACAGAAAAAAG	chrX:31,986,458-31,986,476
Cas9-SLC35A2-1	CCA	ATGCACCCGCGGAAACCGCC	chrX:48768846-48768866
Cas9-SLC35A2-2	CCC	GGGCGCCGCGGTGGAACCAC	chrX:48768874-48768894
Cas9-SLC35A2-3	CCC	CTGGCAACGTGCGGGCGTAG	chrX:48767180-48767200
Cas9-SLC35A2-4	CCA	CCACTGCTGTGGTCATGGCG	chrX:48767142-48767162
Cas9-SLC35A2-5	CCT	GGTGCAGTATGTGGACACGC	chrX:48763757-48763777
Cas9-SLC35A2-6	CCT	TGCAGAATAACCTCCAGTAT	chrX:48763704-48763724

Supplementary Table 10. Primers used for cloning and genotyping.

Name	Sequence (5' to 3')	Note
EMX1-Small-F	GGGCCTCCTGAGTTTCTCAT	Genotyping of EMX1
EMX1-Small-R	AGGGAGTGGCCAGAGTCC	
EMX1-3.8k-F	ATGTCACTCTCATCTCCTAATACAC	
EMX1-1.7k-F	GGGCTTCTCCTGACTGTTCCTTGTGTGACC	
EMX1-1.7k&3.8k-R	TGGCCCTTCCCTATGTCTAGCCTGT	
hCCR5-Small-F	TTGGTGAGATGGTGCTTTCA	Genotyping of CCR5
hCCR5-Small-R	GCTCTTCAGCCTTTTGCAGT	
hCCR5-0.9k-F	GGCTTGCTCATAGTGCATGTTCTTTGTGGG	
hCCR5-0.9k-R	AAACAGGTCAGAGATGGCCAGGTTGAGCAG	
hCCR5-9.7k-F	AAGTCAAGGCCAAAAGCTCA	
hCCR5-9.7k-R	TAAAGATGATTCCTGGGAGAGACGC	
HiDi-HA1k_F	GTGGACCATCCTCTAGACTGCCG	Confirmation of SNP-KI
HiDi-EGFP-CmutG-R	TTCACCTCGGCGCGGGTCTTC	
HiDi-EGFP-Original-R	TTCACCTCGGCGCGGGTCTTG	
EGFP-KI-seq_F	GTGGTGCCCATCCTGGTC	Sequencing of SNP-KI
EGFP-KI-seq_R	GGCCTTCCATCTGTTGCTG	
GFP-up-inv-F	TAGGGCCAGACTGTTACCAC	Detection of inversion
GFP-up-inv-R	GGGCTGGATAAAGGGAGGAT	
GFP-down-inv-F	AAGAACTCTTCCTCACGCGC	
GFP-down-inv-R	TCGTAGAAGGGGAGGTTGC	
EMX1-OT-1-F1	TGCTGGTGTGTTTCCACTGT	checking for OT mutation
EMX1-OT-1-R1	TGTACGTGTGAGGGCATGTT	
EMX1-OT-3-1-F1	GGATTGATTCACAACCACCA	
EMX1-OT-3-1-R1	AGACCGACTCCTGTCTGGAA	
EMX1-OT-3-1-F2	TCACTCCCACGCACATACAT	
EMX1-OT-3-1-R2	TTCTGTTGACATCAATTGACCA	
EMX1-OT-3-2-F1	CCCAACAGACCCCAGTGA	
EMX1-OT-3-2-R1	CGTTTATCCACCACGATGAA	
EMX1-OT-3-3-F1	ATTCCCAGTGCAGGATTCAC	
EMX1-OT-3-3-R1	GGTTGGGAATAAAGATAGCTGAA	
EMX1-OT-3-3-F2	TCTCCCTCCAGAAGAAACCA	
EMX1-OT-3-3-R2	TTACGTGCAGGGCTGGATAC	
EMX1-OT-5-1-F1	CACTAAAATCCTAATGCTTTCATTGT	
EMX1-OT-5-1-R1	CAAAGGCTTCCTCAGCTTACA	
EMX1-OT-5-1-F2	TACACATAGGCCCAAAAGGAA	
EMX1-OT-5-1-R2	TGGTTTGGGGAAATTTCTGA	
EMX1-OT-5-1-F3	ATGCCAGTTAGGCTGCAAGT	
EMX1-OT-5-1-R3	CCCAGTGGCTGGTCTTTATC	
EMX1-OT-5-1-F4	GTAGTGCCTGGCAAACAGGA	
EMX1-OT-5-1-R4	CTCCCATGACATGTGGGAAT	
EMX1-OT-5-2-F1	TGTGCTTTGGAAGTGTGAAGA	
EMX1-OT-5-2-R1	TGATTCAGCTTGCTGCAGTT	
EMX1-OT-5-2-F2	ACAGAGGGTGCGACAACAAT	
EMX1-OT-5-2-R2	TTTCCAGTCAGGGAAACACA	
EMX1-OT-5-2-F3	ACTCGATCCCCATTTGTGAG	
EMX1-OT-5-2-R3	CATGCAGCTGAAGCCATTTA	
EMX1-OT-6-1-F1	AAAATTAGCTGGGCATCGTG	
EMX1-OT-6-1-R1	CACCAGGCAAGAATCGTTTT	
EMX1-OT-6-1-F2	TGTGCAAACACTTCCAGAGG	
EMX1-OT-6-1-R2	AGAACCTGGCACAAGAGAGG	
EMX1-OT-6-2-F1	GCCACAGGGGAAGATTGTAA	
EMX1-OT-6-2-R1	TCAGCTGAATAAGGCACACG	
EMX1-OT-6-2-F2	TGGTCTGTGGGTGAAAATGA	
EMX1-OT-6-2-R2	TGACTTCCCCTTAAGCAAATC	
EMX1-OT-7-1-F1	TTGCCAAAGAAAACCAGAGC	
EMX1-OT-7-1-R1	CTTGCTTTCTTTGCCCTTTG	
EMX1-OT-7-1-F2	ATCCATTCCAGAAGGTTCCA	
EMX1-OT-7-1-R2	CACCTCATCACGAAAGCAGA	
EMX1-OT-7-2-F1	CAAGAAAAGCGGTAAATAACCA	

EMX1-OT-7-2-R1	TGACAAGTTCTGTTTTCCACTCA	
EMX1-OT-10-1-F1	AGCAGAGTTCTGGCCAGTGT	
EMX1-OT-10-1-R1	GCATTCTAAATCCAAGGTGCT	
EMX1-OT-18-1-F1	CCATTCCCACCATCCAATAG	
EMX1-OT-18-1-R1	AGGCTGAAGCCTCTCTCCTG	
EMX1-OT-18-1-F2	GAGACAGGATGCCATCTCCA	
EMX1-OT-18-1-R2	AGATCTCCAGCCCAGACTCA	
EMX1-OT-18-2-F1	TGGAAAGAGCTTGTGTACCG	
EMX1-OT-18-2-R1	TCCCAATGCTTTTGAAGGAC	
EMX1-OT-18-3-F1	GCAGCAAGTGGAAGTTAGGC	
EMX1-OT-18-3-R1	GCAAGTTCACTCTGGCATCA	
EMX1-OT-18-3-F2	AGGCTCAGCAAAGTGGAAAA	
EMX1-OT-18-3-R2	TGTTATATCAGCCTCATGATCTCAA	
EMX1-OT-X-1-F1	CAGTGATTTTGCGCTTTCAA	
EMX1-OT-X-1-R1	GACCCGCTCTCGATTTCATA	
EMX1-OT-X-1-F2	GGAACTGCATCCCTTCACAT	
EMX1-OT-X-1-R2	AGACGGTAGCAAGGGAGGAT	
B2M_genotyping_F	TAACCTGGCACTGCGTCG	Genotyping of B2M
B2M_genotyping_R	TGGGATGGGACTCATTCAGG	
DMD_genotyping_F	CCTCAGGTGCTCCCACAAGGTTATC	Genotyping of DMD in iPSCs
DMD_genotyping_R	TTCCTAATTACAGCCAAATCCCTAGCTGTA	
DMD_RT-PCR_F	ACAAAGCTCAGGTCGGATTG	RT-PCR of DMD
DMD_RT-PCR_R	AGTTGCTGCTCTTTTCCAGGT	
EMX1-F1-nanopore	TTTCTGTTGGTGCTGATATTGCATGTCACTCTCATCTCCTAATACAC	Nanopore-seq
EMX1-R1-nanopore	ACTTGCCTGTCGCTCTATCTTCCAGGATGGCCCTTCCCTATGTCTAGCCTGT	
DMD-F1-nanopore	TTTCTGTTGGTGCTGATATTGCGGACCAGCAGTATTGGCATTTTTTT	
DMD-R1-nanopore	ACTTGCCTGTCGCTCTATCTTCAGTTTTGGTGGCTACGCCATC	
TET2-Amp-F	ACCAACCATGTTGAGGGCAA	Genotyping of TET2
TET2-Amp-R	CTGCCTAGCTGTCTCTCCAG	
AAVS1-Amp-F	CGCCTCTAAAGCTCTCAGCC	Genotyping of AAVS1
AAVS1-Amp-R	CCCCTATGTCCACTTCAGGA	
VEGFA-Amp-F	TCCAGGAGTGGTGGGCATAT	Genotyping of VEGFA
VEGFA-Amp-R	GTCACAGCAGCCACCAAAAG	
ERCC4-Amp-F	AACCACTGGTCCAGCTGAAC	Genotyping of ERCC4
ERCC4-Amp-R	TGCTGAGATCTACACTGGAGGA	
Cas3-qPCR-F	CAGCACATCGAGGAGCTGAAC	Copy number check
Cas3-qPCR-R	CATCCATCTCGGCATCGTCA	

Supplementary Table 11. Sequences for the knock-in with Type 1-E CRISPR.

Name	Sequence of homology arms (5' to 3')
Donor plasmid	
pCR2.1-HA1k-GFP	GCAAGGGCGAGGAGGATAACATGGCCATCATCAAGGAGTTCATGCGCTTCAAGGTGCACATGGAGGGCTCCGTGAACGGCCACGAGTTCGAGATCGAG<br>GGCGAGGGCGAGGGCCGCCCCTACGAGGGCACCCAGACCGCCAAGCTGAAGGTGACCAAGGGTGGCCCCCTGCCCTTCGCCTGGGACATCCTGTCCCC<br>TCAGTTCATGTACGGCTCCAAGGCCTACGTGAAGCACCCCGCCGACATCCCCGACTACTTGAAGCTGTCCTTCCCCGAGGGCTTCAAGTGGGAGCGCGT<br>GATGAACTTCGAGGACGGCGGCGTGGTGACCGTGACCCAGGACTCCTCCCTCCAGGACGGCGAGTTCATCTACAAGGTGAAGCTGCGCGGCACCAACT<br>TCCCCTCCGACGGCCCCGTAATGCAGAAGAAGACCATGGGCTGGGAGGCCTCCTCCGAGCGGATGTACCCCGAGGACGGCGCCCTGAAGGGCGAGATC<br>AAGCAGAGGCTGAAGCTGAAGGACGGCGGCCACTACGACGCTGAGGTCAAGACCACCTACAAGGCCAAGAAGCCCGTGCAGCTGCCCGGCGCCTACA<br>ACGTCAACATCAAGTTGGACATCACCTCCCACAACGAGGACTACACCATCGTGGAACAGTACGAACGCGCCGAGGGCCGCCACTCCACCGGCGGCATG<br>GACGAGCTGTACAAGGGAAGCGGAGCTACTAACTTCAGCCTGCTGAAGCAGGCTGGAGACGTGGAGGAGAACCCTGGACCTATGGTGAGCAAGGGCG<br>AGGAGCTGTTCACCGGGGTGGTGCCCATCCTGGTCGAGCTGGACGGCGACGTAAACGGCCACAAGTTCAGCGTGTCCGGCGAGGGCGAGGGCGATGCC<br>ACCTACGGCAAGCTGACCCTGAAGTTCATCTGCACCACCGGCAAGCTGCCCGTGCCCTGGCCCACCCTCGTGACCACCCTGACCTACGGCGTGCAGTGC<br>TTCAGCCGCTACCCCGACCACATGAAGCAGCACGACTTCTTCAAGTCCGCCATGCCCGAAGGCTACGTCCAGGAGCGCACCATCTTCTTCAAAGATGAC<br>GGAAACTA**C**AAGACCCGCGCCGAGGTGAAGTTCGAGGGCGACACCCTGGTGAACCGCATCGAGCTGAAGGGCATCGACTTCAAGGAGGACGGCAACA<br>TCCTGGGGCACAAGCTGGAGTACAACTACAACAGCCACAACGTCTATATCATGGCCGACAAGCAGAAGAACGGCATCAAGGTGAACTTCAAGATCCGC<br>CACAACATCGAGGACGGCAGCGTGCAGCTCGCCGACCACTACCAGCAGAACACCCCCATCGGCGACGGCCCCGTGCTGCTGCCCGACAACCACTACCT<br>GAGCACCCAGTCCGCCCTGAGCAAAGACCCCAACGAGAAGCGCGATCACATGGTCCTGCTGGAGTTCGTGACCGCCGCCGGGATCACTCTCGGCATGG<br>ACGAGCTGTACAAGTAACTCGAGCGGCCGCCAGCACAGTGGTCGACAGCTGTGGAATGTGTGTCAGTTAGGGTGTGGAAAGTCCCCAGGCTCCCCAGC<br>AGGCAGAAGTATGCAAAGCATGCATCTCAATTAGTCAGCAACCAGGTGTGGAAAGTCCCCAGGCTCCCCAGCAGGCAGAAGTATGCAAAGCATGCATC<br>TCAATTAGTCAGCAACCATAGTCCCGCCCCTAACTCCGCCCATCCCGCCCCTAACTCCGCCCAGTTCCGCCCATTCTCCGCCCCATGGCTGACTAATTTTT<br>TTTATTTATGCAGAGGCCGAGGCCGCCTCGGCCTCTGAGCTATTCCAGAAGTAGTGAGGAGGCTTTTTTGGAGGCCTAGGCTTTTGCAAAAAGCTTACC<br>ATGACCGAGTACAAGCCCACGGTGCGCCTCGCCACCCGCGACGACGTCCCCAGGGCCGTACGCACCCTCGCCGCCGCGTTCGCCGACTACCCCGCCACG<br>CGCCACACCGTCGATCCGGACCGCCACATCGAGCGGGTCACCGAGCTGCAAGAACTCTTCCTCACGCGCGTCGGGCTCGACATCGGCAAGGTGTGGGTC<br>GCGGACGACGGCGCCGCGGTGGCGGTCTGGACCACGCCGGAGAGCGTCGAAGCGGGGGCGGTGTTCGCCGAGATCGGCCCGCGCATGGCCGAGTTGA<br>GCGGTTCCCGGCTGGCCGCGCAGCAACAGATGGAAGGCC
