# Supplementary material for: CRISPR-Cas3 induces broad and unidirectional genome editing in human cells
Source: Nat Commun. 2019 Dec 6;10:5302. doi: 10.1038/s41467-019-13226-x (PMC6897959; doi:10.1038/s41467-019-13226-x)
Supplement: Supplementary file 2 — Reporting Summary [file 41467_2019_13226_MOESM2_ESM.pdf]

nature research

Corresponding author(s): Tomoji Mashimo, Akitsu Hotta, Junji Takeda

Last updated by author(s): Oct 5, 2019

Reporting Summary

Nature Research wishes to improve the reproducibility of the work that we publish. This form provides structure for consistency and transparency in reporting. For further information on Nature Research policies, see Authors & Referees and the Editorial Policy Checklist.

Statistics

For all statistical analyses, confirm that the following items are present in the figure legend, table legend, main text, or Methods section.

n/a	Confirmed	
[ ]	[x]	The exact sample size (*n*) for each experimental group/condition, given as a discrete number and unit of measurement
[ ]	[x]	A statement on whether measurements were taken from distinct samples or whether the same sample was measured repeatedly
[ ]	[x]	The statistical test(s) used AND whether they are one- or two-sided<br>*Only common tests should be described solely by name; describe more complex techniques in the Methods section.*
[x]	[ ]	A description of all covariates tested
[ ]	[x]	A description of any assumptions or corrections, such as tests of normality and adjustment for multiple comparisons
[ ]	[x]	A full description of the statistical parameters including central tendency (e.g. means) or other basic estimates (e.g. regression coefficient) AND variation (e.g. standard deviation) or associated estimates of uncertainty (e.g. confidence intervals)
[x]	[ ]	For null hypothesis testing, the test statistic (e.g. *F*, *t*, *r*) with confidence intervals, effect sizes, degrees of freedom and *P* value noted<br>*Give P values as exact values whenever suitable.*
[x]	[ ]	For Bayesian analysis, information on the choice of priors and Markov chain Monte Carlo settings
[x]	[ ]	For hierarchical and complex designs, identification of the appropriate level for tests and full reporting of outcomes
[ ]	[x]	Estimates of effect sizes (e.g. Cohen's *d*, Pearson's *r*), indicating how they were calculated

Our web collection on statistics for biologists contains articles on many of the points above.

Software and code

Policy information about availability of computer code

Data collection	GGGenome computer program (https://GGGenome.dbcls.jp/en/)
Data analysis	CrispRVariantsLite (http://imlspenticton.uzh.ch:3838/CrispRVariantsLite/), R (https://cran.r-project.org/index.html) , Samtools (http://samtools.sourceforge.net/), Bedtools (https://bedtools.readthedocs.io/en/latest/), Microsoft Excel.

For manuscripts utilizing custom algorithms or software that are central to the research but not yet described in published literature, software must be made available to editors/reviewers. We strongly encourage code deposition in a community repository (e.g. GitHub). See the Nature Research guidelines for submitting code & software for further information.

Data

Policy information about availability of data

All manuscripts must include a data availability statement. This statement should provide the following information, where applicable:

- Accession codes, unique identifiers, or web links for publicly available datasets
- A list of figures that have associated raw data
- A description of any restrictions on data availability

The data that support the findings of this study are available from the corresponding authors upon reasonable request.

Field-specific reporting

Please select the one below that is the best fit for your research. If you are not sure, read the appropriate sections before making your selection.

[x] Life sciences   [ ] Behavioural & social sciences   [ ] Ecological, evolutionary & environmental sciences

Life sciences study design

All studies must disclose on these points even when the disclosure is negative.

Sample size	The most experiments run in triplicate.
Data exclusions	No data was excluded from analysis.
Replication	The most experiments were repeated and we confirmed the reproducibility of data.
Randomization	The experiments were not randomized.
Blinding	Investigators were not blinded to allocation during experiments and outcome assessment.

Reporting for specific materials, systems and methods

We require information from authors about some types of materials, experimental systems and methods used in many studies. Here, indicate whether each material, system or method listed is relevant to your study. If you are not sure if a list item applies to your research, read the appropriate section before selecting a response.

Materials & experimental systems

n/a	Involved in the study	
[ ]	[x]	Antibodies
[ ]	[x]	Eukaryotic cell lines
[ ]	[x]	Palaeontology
[x]	[ ]	Animals and other organisms
[x]	[ ]	Human research participants
[ ]	[ ]	Clinical data

Methods

n/a	Involved in the study	
[x]	[ ]	ChIP-seq
[ ]	[x]	Flow cytometry
[x]	[ ]	MRI-based neuroimaging

Antibodies

Antibodies used	anti-Dystrophin (applicable, Abcam, ab15277, polyclonal,GR139183-1)<br>anti-MHC (applicable, R&D Systems, MAB4470, MF20, CAEI0818031)<br>anti-rabbit IgG HRP-linked antibody (applicable, ProteinSimple, 042-206, n/a, #82554)<br>anti-mouse IgG HRP-linked antibody (applicable, ProteinSimple, 042-205, n/a, #83095)<br>anti-HLA-A2-BD Horizon™ BV421 conjugated (applicable, Becton Dickinson, #740082, BB7.2, 8243917)
Validation	anti-Dystrophin (human, Wes™ Simple Western system)<br>anti-MHC (human, Wes™ Simple Western system)<br>anti-HLA-A2-BD Horizon™ BV421 conjugated (human, flow cytometry)

Eukaryotic cell lines

Policy information about cell lines

Cell line source(s)	HEK293T, iPSC
Authentication	The human iPS cell line 1383D2
Mycoplasma contamination	No contamination.
Commonly misidentified lines<br>(See ICLAC register)	No cell lines are on the database of commonly misidentified lines

Palaeontology

Specimen provenance	n/a
Specimen deposition	n/a

Dating methods	n/a

[x] Tick this box to confirm that the raw and calibrated dates are available in the paper or in Supplementary Information.

Clinical data

Policy information about clinical studies

All manuscripts should comply with the ICMJE guidelines for publication of clinical research and a completed CONSORT checklist must be included with all submissions.

Clinical trial registration	n/a
Study protocol	n/a
Data collection	n/a
Outcomes	n/a

Flow Cytometry

Plots

Confirm that:

[x] The axis labels state the marker and fluorochrome used (e.g. CD4-FITC).

[x] The axis scales are clearly visible. Include numbers along axes only for bottom left plot of group (a 'group' is an analysis of identical markers).

[x] All plots are contour plots with outliers or pseudocolor plots.

[x] A numerical value for number of cells or percentage (with statistics) is provided.

Methodology

Sample preparation	Cultured cell lines were collected from dishes.
Instrument	FACS Aria IIIu (Becton Dickinson) were used.
Software	FACS data were analyzed with FACS Diva and Flowjo 10.4.
Cell population abundance	All collected cells from a well were sorted and more than 10,000 cells were tested.
Gating strategy	FSC vs SSC gating was used for detecting a single cell and the other gating strategies were described in methods.

[x] Tick this box to confirm that a figure exemplifying the gating strategy is provided in the Supplementary Information.
