# Supplementary material for: CRISPR-Cas3 induces broad and unidirectional genome editing in human cells
Source: Nat Commun. 2019 Dec 6;10:5302. doi: 10.1038/s41467-019-13226-x (PMC6897959; doi:10.1038/s41467-019-13226-x)
Supplement: Supplementary file 3 — Peer Review File [file 41467_2019_13226_MOESM3_ESM.pdf]

Editorial Note: This manuscript has been previously reviewed at another journal that is not operating a transparent peer review scheme. The manuscript was considered suitable for publication without further review at *Nature Communications*.
